# Vinyl copolymers with faster hydrolytic degradation than aliphatic polyesters and tunable upper critical solution temperatures

**DOI:** 10.1038/s41467-022-30220-y

**Published:** 2022-05-24

**Authors:** Amaury Bossion, Chen Zhu, Léa Guerassimoff, Julie Mougin, Julien Nicolas

**Affiliations:** Université Paris-Saclay, CNRS, Institut Galien Paris-Saclay, 92296 Châtenay-Malabry, France

**Keywords:** Polymer synthesis, Biopolymers

## Abstract

Vinyl polymers are the focus of intensive research due to their ease of synthesis and the possibility of making well-defined, functional materials. However, their non-degradability leads to environmental problems and limits their use in biomedical applications, allowing aliphatic polyesters to still be considered as the gold standards. Radical ring-opening polymerization of cyclic ketene acetals is considered the most promising approach to impart degradability to vinyl polymers. However, these materials still exhibit poor hydrolytic degradation and thus cannot yet compete with traditional polyesters. Here we show that a simple copolymerization system based on acrylamide and cyclic ketene acetals leads to well-defined and cytocompatible copolymers with faster hydrolytic degradation than that of polylactide and poly(lactide-*co*-glycolide). Moreover, by changing the nature of the cyclic ketene acetal, the copolymers can be either water-soluble or can exhibit tunable upper critical solution temperatures relevant for mild hyperthermia-triggered drug release. Amphiphilic diblock copolymers deriving from this system can also be formulated into degradable, thermosensitive nanoparticles by an all-water nanoprecipitation process.

## Abstract (web summary)

The non-degradability of vinyl polymers has long limited their use in biomedical applications. In this article, the authors demonstrate a system based on acrylamide and cyclic ketene acetals to obtain copolymers with faster degradation rates for potential drug release and environmental applications.

## Introduction

Vinyl polymers are attractive materials owing to their ease of synthesis and their broad diversity in terms of architectures, compositions, and functionalities, especially since the advent of reversible deactivation radical polymerization (RDRP)^1–3^. However, they are not degradable because of their carbon backbone, which creates environmental issues and severely limits their use for biomedical applications. Therefore, aliphatic polyesters are still the gold standards, especially for biomedical applications given their biocompatibility and degradability. Yet, the possibility to easily functionalize them and tune their structure and composition to obtain advanced materials are rather restricted. Consequently, combining the advantages of both polymer families to produce next-generation degradable materials is still an unmet need.

In this context, a lot of effort has been made to synthesize degradable vinyl polymers^4^. One of the most potent approaches relies on introducing ester groups in the polymer backbone by radical ring-opening polymerization (rROP) of cyclic ketene acetals (CKAs)^5,6^. Among them, 2-methylene-1,3-dioxepane (MDO), 5,6-benzo-2-methylene-1,3-dioxepane (BMDO) or 2-methylene-4-phenyl-1,3-dioxolane (MPDL) are by far the most used ones^7,8^. For example, copolymerization of common vinyl monomers with CKAs by conventional free-radical polymerization or RDRP has received tremendous attention^8^ to design degradable materials for applications in drug delivery^9–11^, marine antibiofouling technologies^12–14^, gene/DNA transfection^15^, tissue engineering^16^ and others^17,18^.

Yet, despite promising proofs of concept, the poor hydrolytic degradation of CKA-containing copolymers under physiological conditions remains a significant limitation. Indeed, even though their hydrolytic degradation under accelerated conditions is rapid, their degradation under physiological conditions usually takes from several months to a year to achieve at least a 50% decrease in molar mass, even with oligo(ethylene glycol) methyl ether methacrylate (OEGMA) as the main vinyl monomer^19–21^, which may be problematic for some (bio-related) applications. Therefore, such materials still cannot compete with the most popular polyesters like poly(lactic-*co*-glycolic acid) (PLGA) or even poly(lactic acid) (PLA). Their enzymatic degradation is also very limited as only copolymers comprising polycaprolactone (PCL)-like MDO units showed significant degradation in the presence of specific enzymes (e.g., lipases)^21^, whereas the bulky aromatic ring and/or the too high hydrophobicity of BMDO and MPDL drastically obstruct enzyme access^20,22^. In addition, for the design of materials with advanced physico-chemical, self-assembly, or stimuli-responsive properties^8^, the CKA is often used at least as a third comonomer and for a single goal; that is conferring degradability. This makes the synthesis more complex and, due to the high hydrophobicity of CKAs and/or side reactions during polymerizations, often has a detrimental impact on the targeted properties (e.g., the solubility of water-soluble copolymers^11^, colloidal stability, and particle size distribution of nanoparticles^23^, stimuli-responsiveness of materials^24,25^). Therefore, allowing the CKA to combine two different properties would be important progress.

All these limitations are of utmost importance and represent serious obstacles to the development of advanced vinyl polymers for biomedical applications, such as thermoresponsive vinyl polymers which hold great promise for potential applications in bioengineering and nanomedicine^26^. They can exhibit either a lower critical solution temperature or an upper critical solution temperature (UCST); the latter being considered a very attractive feature for mild hyperthermia-triggered drug release. However, not only degradable, thermoresponsive vinyl polymers have received very little attention^17,22,27–32^, especially UCST polymers^33,34^, but they all exhibit poor hydrolytic degradation and the synthesis of well-defined, UCST vinyl copolymers that can degrade in water has never been reported.

**Fig. 1:**
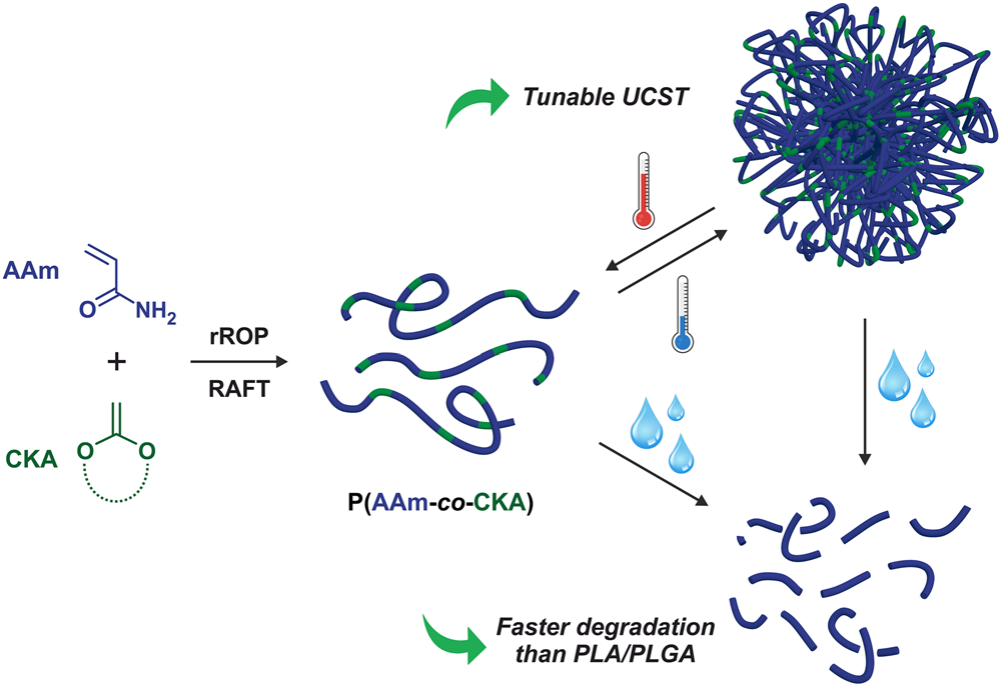
Design of hydrolytically degradable, thermosensitive vinyl copolymers. Synthesis of well-defined poly(acrylamide-*co*-cyclic ketene acetal) (P(AAm-*co*-CKA)) copolymers by reversible addition−fragmentation chain transfer (RAFT) copolymerization between AAm and CKAs, that exhibit tunable and biologically relevant upper critical solution temperature (UCST), and faster hydrolytic degradation than polylactide (PLA) and poly(lactic-*co*-glycolic acid) (PLGA).

Herein, we report on a copolymerization system based on acrylamide (AAm) that circumvents the previously mentioned limitations associated with the rROP of CKAs. It enabled the synthesis of well-defined and cytocompatible vinyl copolymers exhibiting: (i) a rapid hydrolytic degradation in water or PBS, faster than that of PLA and even PLGA, which is unprecedented in the field of vinyl materials, and (ii) complete water-solubility or a tunable and sharp UCST in practically relevant conditions for which the CKA is an integral part of the thermosensitivity mechanism, thus greatly simplifying the system (Fig. 1). To demonstrate the great interest in these building blocks for biomedical applications, we also synthesized amphiphilic PEG-based diblock copolymers that were formulated into nanoparticles (NPs) by an all-water nanoprecipitation process owing to their UCST. It, therefore, avoided the use of organic solvents that are often problematic for pharmaceutical development, which is also unprecedented in the pharmaceutical field. These nanoparticles exhibited both UCST and LCST transitions, thus leading to doubly thermosensitive, degradable nanoparticles.

## Results

### Design rationale

**Fig. 2:**
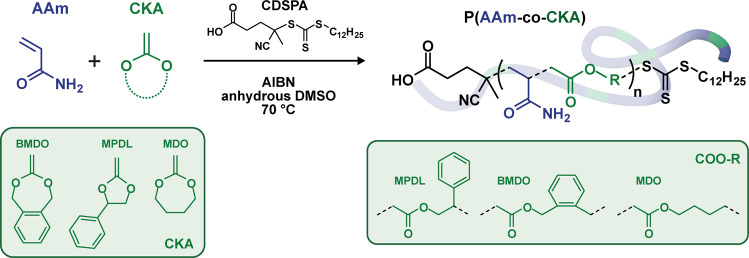
Synthesis of poly(acrylamide-*co*-cyclic ketene acetal) (P(AAm-*co*-CKA)) copolymers. RAFT-mediated copolymerization between acrylamide (AAm) and cyclic ketene acetals (CKA), such as 2-methylene-4-phenyl-1,3-dioxolane (MPDL), 5,6-benzo-2-methylene-1,3-dioxepane (BMDO) and 2-methylene-1,3-dioxepane (MDO).

The design of the copolymerization system relied on a simple structural analogy with poly(acrylamide-*co*-styrene) (P(AAm-*co*-S)) copolymers which are known to exhibit a UCST in aqueous solution in the 50–62 °C range, owing to reversible hydrogen bonds between the copolymer chains below the UCST and with water molecules above^35^. These copolymers however, received little attention compared to their acrylonitrile-containing UCST counterparts; namely poly(acrylamide-*co*-acrylonitrile) (P(AAm-*co*-AN)) copolymers^26,36^. Nonetheless, we postulated that substituting styrene with aromatic ring-containing CKA units, such as MPDL or BMDO, could be a straightforward route toward degradable, UCST copolymers in which the CKA would both confer degradability and govern the thermoresponsiveness, ideally in a much broader temperature range. It was also postulated that this copolymerization system would be compatible with RDRP to give access to well-defined architectures and that these would be more prone to hydrolytic degradation than any other CKA-containing vinyl copolymers synthesized so far given the very high water-solubility of AAm moieties that would promote efficient solvation of ester groups (Fig. 2).

### Proof of concept with P(AAm-*co*-MPDL) and P(AAm-*co*-MDO) copolymers

**Fig. 3:**
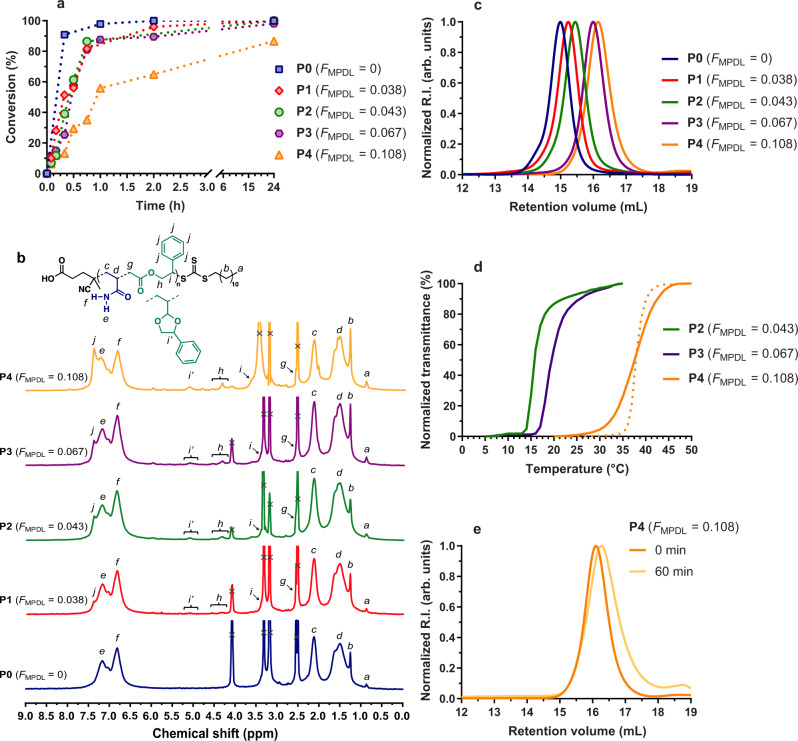
Copolymerization kinetics and characterization of P(AAm-*co*-MPDL) copolymers. **a** Conversion vs. time kinetic plot from the RAFT copolymerization of AAm with MPDL (Table 1, **P0**–**P4**) in anhydrous DMSO initiated by AIBN at 70 °C. Conversion = AAm conversion as determined by ^1^H NMR; **b** ^1^H NMR spectrum (300 MHz, DMSO-d_6_) in the 0–9 ppm region of P(AAm-*co*-MPDL) copolymers **P0**–**P4** (Table 1); **c** evolution of the SEC chromatograms of P(AAm-*co*-MPDL) copolymers **P0**–**P4** as function of *F*_MPDL_; **d** variation of the solution transmittance vs. temperature of P(AAm-*co*-MPDL) copolymers **P2**–**P4** solution in water at 10 mg mL^−1^ (1 °C min^−1^, solid and dotted lines for cooling and heating, respectively); **e** evolution of the SEC chromatograms at a different time during the hydrolytic degradation under accelerated conditions (5 wt.% KOH) of P(AAm-*co*-MPDL) copolymer **P4**.

To determine the most suitable aromatic ring-containing CKA to confer degradability and govern the thermosensitivity, the first copolymerizations were made with MPDL because of its open radical structure very close to that of styrene. P(AAm-*co*-MPDL) copolymers (**P0**–**P4**, Table 1) were obtained by RAFT-mediated copolymerization of AAm and variable initial molar fractions of MPDL (*f*_MPDL,0_ = 0–0.8) at 8 M in anhydrous dimethyl-sulfoxide (DMSO) for 16 h using 4-cyano-4-[(dodecylsulfanylthiocarbonyl)sulfanyl]pentanoic acid (CDSPA) as chain transfer agent (CTA) and azobisisobutyronitrile (AIBN) as the initiator. High monomer conversions were obtained and the copolymers exhibited molecular weights in the 8.3–28.3 kg mol^−1^ range with mostly low dispersities (*Đ* = 1.2–1.3) (Fig. 3a, c). By ^1^H nuclear magnetic resonance (NMR), it was shown that the molar fraction of MPDL in the copolymer (*F*_MPDL_) varied from 0.038 to 0.108 with 46% open MPDL units on average (Fig. 3b). Also, the more MPDL in the comonomer feed, the lower the molecular weight, as previously seen with other CKA/vinyl monomer pairs^23,37,38^.

**Table 1:** Experimental conditions and macromolecular characteristics of P(AAm-*co*-MPDL) and P(AAm-*co*-MDO) copolymers synthesized by RAFT-mediated copolymerization of AAm and MPDL (or MDO) in anhydrous DMSO at 70 °C for 16 h.

Entry	CKA	*f*_CKA,0_	*F*_CKA_^a^	Open CKA (%)^a^	AAm conv. (%)^b^	*T*_cp_ from UV (°C)^c^		*M*_n, NMR_^d^ (g mol^−1^)	*M*_n, exp._^e^ (g mol^−1^)	*Đ*_exp._^e^	*M*_n, deg. theo.__._^f^ (g mol^−1^) (% *M*_n_ loss)	*M*_n, deg._^g^ (g mol^−1^) (% *M*_n_ loss)
						Cooling	Heating					
P0	-	0	0	0	>98	-^h^	-^h^	8200	28,300	1.2	-	27,500 (−2.8%)
P1	MPDL	0.2	0.038	51	>98	-^h^	-^h^	6600	22,300	1.3	3800 (−83%)	22,900 (+2%)
P2	MPDL	0.4	0.043	50	>98	15	-^i^	6500	17,800	1.2	3400 (−81%)	15,600 (−12%)
P3	MPDL	0.6	0.067	37	98	18	-^i^	6100	12,500	1.2	3000 (−76%)	10,000 (−20%)
P4	MPDL	0.8	0.108	44	87	38	38	8200	8300	1.2	1600 (−81%)	5700 (−31%)
P5	MDO	0.2	0.095	76	>98	-^h^	-^h^	16,000	29,300	1.7	1000 (−97%)	13,500 (−54%)
P6	MDO	0.4	0.18	43	93	-^h^	-^h^	5600	37,900	2.3	960 (−97%)	7500 (−80%)
P7	MDO	0.6	0.27	53	95	-^h^	-^h^	8500	53,700	3.3	540 (−99%)	6200 (−88%)
P8	MDO	0.8	0.44	43	76	-^j^	-^j^	7100	14,000	4.4	420 (−97%)	1700 (−87%)

^a^Determined by ^1^H NMR after precipitation by: (i) integrating the 2H (–NH_2_) of AAm, the 5H (aromatic protons) of open and closed MPDL (6.5–7.5 ppm), the 2H of open MPDL (4.0–4.4 ppm) and the 1H of closed MPDL (5.0–5.2 ppm) for MPDL and (ii) by integrating the 2H (–NH_2_) of AAm (6.5–7.5 ppm), the 2H of open MDO (3.9–4.1 ppm) and the 4H of closed MDO (3.4–3.8 ppm) for MDO. ^b^Determined by ^1^H NMR by integrating the 2H of AAm (6.02–6.24 ppm) at *t* = 0 and 16 h. ^c^Determined from the maximum of the first derivative of the cooling/heating curves obtained by UV-vis temperature ramp (1 °C min^−1^) at 10 mg mL^−1^ in deionized water. ^d^Determined by ^1^H NMR after precipitation by integrating the 3H from the CH_3_ moiety of the RAFT agent C12 alkyl chain (0.86 ppm), the 1H of AAm (2.1 ppm) and: (i) the 2H of open MPDL (4.0–4.4 ppm) and the 1H of closed MPDL (5.0–5.2 ppm) or (ii) the 2H of open MDO (3.9–4.1 ppm) and the 4H of closed MDO (3.4–3.8 ppm). Note that this method is only accurate for high-living chain fractions. ^e^Determined by SEC in DMSO with 100 mM LiBr. ^f^Determined according to *M*_n, deg. theo_ = ([1/(open CKA × *F*_CKA_)]–1) × MW_AAm_ + MW_CKA_, with MW being the molecular weight of the considered monomer. ^g^Determined by SEC in DMSO with 100 mM LiBr after hydrolytic degradation using KOH 5 wt.% solution. ^h^Samples were water-soluble at any temperature. ^i^Loss of the UCST behavior due to precipitation occurring below the UCST. ^j^Samples were insoluble in water at any temperature.

The solubility of the different copolymers in water (10 mg mL^−1^) was then tested by transmittance measurements performed between 5 and 50 °C (Fig. 3d). While PAAm (**P0**) and P(AAm-*co*-MPDL) with *F*_MPDL_ = 0.038 (**P1**) were fully water-soluble independently of the temperature, the copolymers with higher amounts of MPDL (**P2**–**P4**) showed a sharp UCST-type transition upon cooling. In addition, varying *F*_MPDL_ from 0.043 to 0.108 allowed for fine-tuning the thermosensitivity, as shown by the increase of the cloud point (*T*_cp_) from 15 to 38 °C (Supplementary Fig. 1). P(AAm-*co*-MPDL) copolymers, **P2**, and **P3** gave a UCST upon cooling but not upon heating because of the formation of stable aggregates. However, a reversible transition was observed for the copolymer with the highest MPDL content (**P4**), which gave the same cloud point near body temperature (*T*_cp_ = 38 °C) each time. Degradation of P(AAm-*co*-MPDL) copolymers was then performed under accelerated conditions (i.e., 5 wt.% aqueous potassium hydroxide (KOH) to probe the presence of open ester groups. Whereas P(AAm-*co*-MPDL) copolymer **P1** showed a nearly constant *M*_n_ after degradation because of its too low amount of open MPDL units, P(AAm-*co*-MPDL) copolymers **P2**–**P4** led to a decrease in *M*_n_ up to −31% as shown by size exclusion chromatography (SEC) (Fig. 3e and Supplementary Fig. 2). Although significant, this degradation however appeared lower than expected according to the theoretical *M*_n_ after degradation values (*M*_n, deg. theo_., Table 1). This can be explained not only by the limited amount of MPDL inserted in the copolymer due to the propensity of CKAs to hardly copolymerize with most acrylic monomers, but also by the significant fraction of ring-retained MPDL units.

To confirm the key role of the CKA’s aromatic ring in the establishment of the UCST and therefore the relevance of the structural analogy between P(AAm-*co*-MPDL) and P(AAm-*co*-S) copolymers, we synthesized similar copolymers with MDO as a CKA (Table 1, **P5**–**P8**, Supplementary Figs. 3 and 4). Comparable macromolecular characteristics were obtained despite increasing dispersities when increasing the initial MDO molar fraction (*M*_n, exp_ = 14–53.4 kg mol^−1^, *Đ* = 1.7–4.4, Supplementary Fig. 5). The copolymers were successfully degraded under accelerated conditions with up to 88% decrease in *M*_n_ (Supplementary Fig. 6). However, none of the P(AAm-*co*-MDO) copolymers showed a UCST despite a wide range of compositions (*F*_MDO_ = 0.095–0.44) tested. All copolymers were indeed water-soluble over the 0–100 °C temperature range except the one with the highest MDO content (**P8**), which was insoluble in water (Supplementary Fig. 7). It should be noted that thermoresponsive P(AAm-co-MDO) copolymers can still be obtained, but by a free-radical polymerization process producing ill-defined structures comprising PMDO branches that were responsible for the thermosensitivity, but prevented complete water-solubility of the copolymers, whose degradation has not been demonstrated^33^.

These results confirmed our hypothesis as they showed that a simple RAFT-mediated copolymerization between AAm and an aromatic ring-containing CKA such as MPDL enabled the straightforward synthesis of well-defined, degradable, UCST vinyl copolymers owing to the presence of hydrogen bond donors (NH group of primary amide in AAm units) and acceptors (center of the phenyl rings in MPDL units)^39^.

### Synthesis and evaluation of P(AAm-*co*-BMDO) copolymers

AAm was then copolymerized with BMDO as a second aromatic ring-containing CKA under similar conditions to: (i) assess the feasibility of well-defined, degradable, and UCST P(AAm-*co*-BMDO) copolymers and (ii) to determine the best copolymerization system before further physico-chemical and bio-related investigations. Beyond potential differences in terms of CKA insertion and amount of ring-opened units between MPDL and BMDO, we also suspected that the difference in position of the aromatic ring between those two monomers may impact the establishment of hydrogen bonds and therefore the thermosensitivity.

**Fig. 4:**
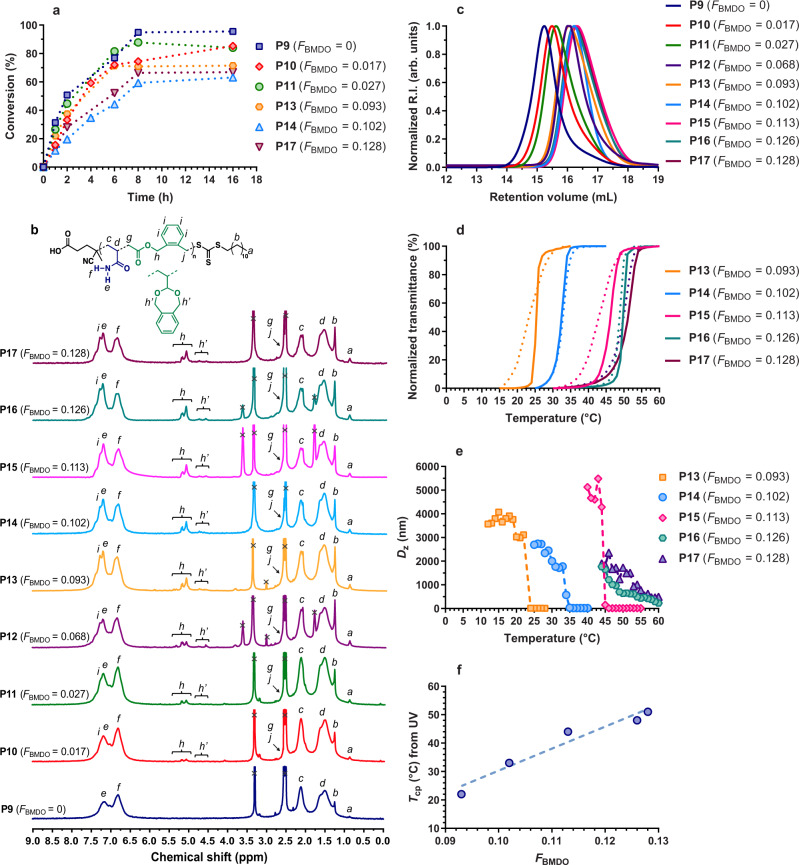
Copolymerization kinetics and characterization of P(AAm-*co*-BMDO) copolymers. **a** Conversion vs. time kinetic plot from the RAFT polymerization of AAm with BMDO in anhydrous DMSO initiated by AIBN at 70 °C. Conversion = AAm conversion as determined by ^1^H NMR; **b** ^1^H NMR spectrum (300 MHz, DMSO-d_6_) in the 0–9 ppm region of P(AAm-*co*-BMDO) copolymers **P9**–**P17** (Table 2); **c** evolution of the SEC chromatograms of P(AAm-*co*-BMDO) copolymers **P9**–**P17** as a function of *F*_BMDO_; **d** variation of the solution transmittance vs. temperature of P(AAm-*co*-BMDO) copolymers **P13**–**P17** solution in water at 10 mg mL^−1^ (1 °C min^−1^, solid and dotted lines for cooling and heating, respectively); **e** variation of the intensity average diameter (*D*_z_) from DLS vs. temperature of P(AAm-*co*-BMDO) copolymers **P13**–**P17** solution in water (10 mg mL^−1^) upon cooling (1 °C min^−1^); **f** evolution of *T*_cp_ measured by UV upon heating as a function of *F*_BMDO_ for copolymers **P13**–**P17** (the dashed blue line is a linear regression as guide for the eyes only).

P(AAm-*co*-BMDO) copolymers (**P9**–**P17**, Table 2) were obtained at 0.8 M in anhydrous DMSO as copolymerizations performed between 2 and 8 M gave too highly viscous solutions in <1 h and greater proportions of closed BMDO (Supplementary Table 1 and Supplementary Figs. 8 and 9). By varying *f*_BMDO,0_ from 0 to 0.55, monomer conversions after 16 h were in the 96–67% range, and the more BMDO in the feed, the lower the conversion (Fig. 4a). The copolymers’ molecular weights ranged from 18.6 to 6.1 kg mol^−1^ with rather low dispersities (*Đ* = 1.2–1.5) (Fig. 4c). Complete copolymerization kinetics was also performed for **P13** via sample withdrawing at regular intervals to make sure of the linear growth of the copolymer with conversion (Supplementary Fig. 10). Remarkably, it was shown by ^1^H NMR that not only greater contents in BMDO could be introduced in comparison with MPDL at similar feed ratios (for instance, *f*_BMDO,0_ = 0.50 and *f*_MPDL,0_ = 0.80 led to relatively close *F*_CKA_ of 0.102 and 0.108, respectively), but the average percentage of ring-opened BMDO units was much greater than with MPDL (89 vs. 46%, respectively), which suggests a greater susceptibility to hydrolysis (Fig. 4b). Given this strong benefit, we, therefore, decided to select the AAm/BMDO copolymerization system for further evaluation. Reactivity ratios of AAm/BMDO were determined to be *r*_AAm_ = 13.02 and *r*_BMDO_ = 0.23, using the nonlinear least-squares method (Supplementary Fig. 11)^40,41^. These values are rather similar to those reported for the copolymerization of BMDO with *N*-isopropylacrylamide (NIPAAm) (*r*_NIPAAm_ = 7.31 and *r*_BMDO_ = 0.11 at 120 °C)^42^.

**Table 2:** Experimental conditions and macromolecular characteristics of P(AAm-*co*-BMDO) copolymers synthesized by RAFT-mediated copolymerization of AAm and BMDO in anhydrous DMSO at 70 °C for 16 h.

Entry	*f*_BMDO,0_	*F*_BMDO_^a^	Open BMDO (%)^a^	AAm conv. (%)^b^	*T*_cp_ from UV (°C)^c^		*T*_cp_ from DLS (°C)^d^		*M*_n, NMR_^e^ (g mol^−1^)	*M*_n, exp._^f^ (g. mol^−1^)	*Đ*_exp._^f^	*M*_n, deg. theo._^g^ (g mol^−1^) (% *M*_n_ loss)	*M*_n, deg._^h^ (g mol^−1^) (% *M*_n_ loss)
					cooling	heating	cooling	heating					
P9	0	0	0	96	-^i^	-^i^	-^i^	-^i^	14,500	18,600	1.5	-	20,800
P10	0.2	0.017	98	85	-^i^	-^i^	-^i^	-^i^	6300	13,500	1.5	4300 (−68%)	7400 (−45%)
P11	0.3	0.027	96	84	-^i^	-^i^	-^i^	-^i^	8800	12,200	1.4	2800 (−77%)	5000 (−59%)
P12	0.35	0.068	87	75	-^i^	-^i^	-^i^	-^i^	5100	7700	1.4	1300 (−83%)	3200 (−58%)
P13	0.4	0.093	88	71	25	22	23	23	6900	7600	1.4	960 (−87%)	2100 (−72%)
P14	0.5	0.102	89	86	33	33	34	36	6900	8400	1.2	870 (−90%)	800 (−90%)
P15	0.51	0.113	90	75	46	44	44	45	6200	6600	1.4	790 (−88%)	1600 (−76%)
P16	0.53	0.126	89	71	49	48	46	46	6100	6400	1.4	720 (−89%)	1700 (−73%)
P17	0.55	0.128	87	67	52	51	55	55	6000	6100	1.4	730 (−88%)	1600 (−74%)

^a^Determined by ^1^H NMR after precipitation by integrating the 2H (–NH_2_) of AAm, the 4H (aromatic protons) of open and closed BMDO (6.5–7.5 ppm), the 2H of open BMDO (4.9–5.2 ppm) and the 4H of closed BMDO (4.5–4.8 ppm). ^b^Determined by ^1^H NMR by integrating the 2H of AAm (6.02–6.24 ppm) at *t* = 0 and 16 h. ^c^Determined from the maximum of the first derivative of the cooling/heating curves obtained by UV-vis temperature ramp (1 °C min^−1^) at 10 mg mL^−1^ in deionized water. ^d^Determined from the maximum of the first derivative of the cooling/heating curves obtained by DLS temperature ramp at 10 mg mL^−1^ in deionized water. ^e^Determined by ^1^H NMR after precipitation by integrating the 3H from the CH_3_ moiety of the RAFT agent C12 alkyl chain (0.86 ppm), the 1H of AAm (2.1 ppm), the 2H of open BMDO (4.9–5.2 ppm) and the 4H of closed BMDO (4.5–4.8 ppm). Note that this method is only accurate for high living chain fractions. ^f^Determined by SEC in DMSO with 100 mM LiBr. ^g^Determined according to: *M*_n, deg. theo_ = ([1/(open BMDO × *F*_BMDO_)] –1) × MW_AAm_ + MW_BMDO_, with MW being the molecular weight of the considered monomer. ^h^Determined by SEC in DMSO with 100 mM LiBr after polymer hydrolytic degradation using KOH 5 wt.% solution. ^i^Samples were water-soluble at any temperature.

As expected, P(AAm-*co*-BMDO) copolymers also exhibited sharp UCST transitions upon both cooling and heating providing they contained enough BMDO (Fig. 4d). Indeed, PAAm (**P9**) and P(AAm-*co*-BMDO) with *F*_BMDO_ = 0.017–0.068 (**P10**–**P12**) were fully soluble in water over the 0–100 °C temperature range, whereas copolymers **P13**–**P17** gave an increase of *T*_cp_ from 25 to 52 °C with *F*_BMDO_ (Supplementary Fig. 12). Importantly, this enabled a range of UCST suitable for biomedical applications to be obtained since both room and body temperatures were covered simply by adjusting the BMDO content in the copolymer. Moreover, using BMDO in place of MPDL enabled fully reversible UCST with minimal hysteresis between cooling and heating to be obtained for all thermosensitive copolymers (Fig. 4d). It also permitted a rather linear increase of *T*_cp_ with *F*_BMDO_ and a much broader range of *T*_cp_ to be covered for a narrower range of CKA content (Δ*T*_cp_ = 27 °C for Δ*F*_BMDO_ = 0.035 vs. Δ*T*_cp_ = 23 °C for Δ*F*_MPDL_ = 0.065) (Fig. 4f). The latter point shows that fine-tuning the *T*_cp_ is not at the expense of strong variations in the copolymer composition and its expected degradation. For comparison, non-degradable P(AAm-*co*-S) vinyl copolymers exhibited UCST behavior over a much narrower range of temperature and copolymer composition (Δ*T*_cp_ = 12 °C for Δ*F*_S_ = 0.02)^35^.

Dynamic light scattering (DLS) was used to monitor the intensity average diameter (*D*_z_) changes of the thermosensitive copolymers in water upon cooling and heating, and accounted for *T*_cp_ values in excellent agreement with those obtained by transmittance measurements (Fig. 4e and Supplementary Fig. 13). Above the UCST, the copolymers were fully soluble and characterized by an average *D*_z_ of ~10 nm whereas, upon cooling, the average *D*_z_ abruptly increased to ~2–5 µm, demonstrating the formation of aggregates. Formation of such aggregates was also monitored by optical microscopy (Supplementary Fig. 14).

**Fig. 5:**
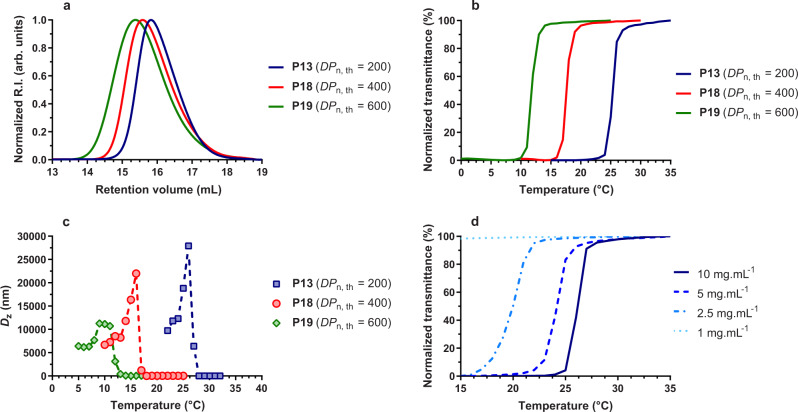
Tuning the UCST of P(AAm-*co*-BMDO) copolymers. **a** Evolution of the SEC chromatograms of P(AAm-*co*-BMDO) copolymers with the targeted average degree of polymerization (*DP*_n, th_); **b** variation of the solution transmittance vs. temperature of P(AAm-*co*-BMDO) copolymers **P13** and **P18**–**P19** solution in water at 10 mg mL^−1^ upon cooling (1 °C min^−1^); **c** Variation of the intensity average diameter (*D*_z_) from DLS vs. temperature of P(AAm-*co*-BMDO) copolymers **P13** and **P18**–**P19** in solution in water (10 mg mL^−1^) upon cooling (1 °C min^−1^) during temperature cycling; **d** variation of the solution transmittance vs. temperature of P(AAm-*co*-BMDO) **P13** at different concentrations in water, i.e., 1, 2.5, 5, and 10 mg mL^−1^ upon cooling (1 °C min^−1^).

To get further insight into the UCST behavior of this copolymerization system, the dependence of the cloud point on the molecular weight for a fixed *f*_BMDO,0_ value and on the copolymer concentration was also investigated. Additional copolymerizations with *f*_BMDO,0_ = 0.4 were first performed by targeting average degrees of polymerization, *DP*_n, th_, of 400 and 600 (**P18** and **P19**, respectively, Supplementary Table 2) to be compared with **P13** (*DP*_n, th_ = 200). The higher *DP*_n, th_, the higher the molecular weight (*M*_n, exp_ = 7.6–10.2 kg mol^−1^, *Đ* = 1.4–1.8), but the lower the conversion and the BMDO content (Fig. 5a and Supplementary Fig. 15). These results are in line with the unfavorable reactivity ratios that give gradient-type copolymers resulting in a gradual enrichment in CKA along the copolymer chain. Therefore, when the conversion is lower, so is the BMDO content. This has direct consequences on the UCST of the copolymers as the *T*_cp_ was shifted toward lower temperatures (from ~23 to ~12 °C as measured by transmittance and DLS), as the result of longer AAm sequences that promote polymer–water interactions (Fig. 5b and c).

The dependence of the UCST on the copolymer concentration was then studied by measuring the *T*_cp_ of aqueous solutions of P(AAm-*co*-BMDO) **P13** at different concentrations ranging from 1 to 10 mg mL^−1^ (Fig. 5d). Similar to non-degradable AAm-based UCST copolymers^35,43,44^, the *T*_cp_ gradually decreased with the copolymer dilution (from 25 °C at 10 mg mL^−1^ to 20 °C at 2.5 mg mL^−1^), until the copolymer loses its UCST at 1 mg mL^−1^. At low copolymer concentration, the copolymer-copolymer intra- and intermolecular hydrophobic interactions were less favored compared to copolymer-water hydrophilic interactions resulting in lower *T*_cp_ values.

Overall, targeting different *DP*_n_ at a fixed *f*_BMDO,0_ value, and varying the copolymer concentration also independently offered the possibility to adjust the thermal response of P(AAm-*co*-BMDO) copolymers.

### Hydrolytic and enzymatic degradation of the copolymers

The degradation of P(AAm-*co*-BMDO) copolymers was then evaluated under three different conditions: (i) hydrolytic degradation under accelerated conditions (5 wt.% KOH, r.t.); (ii) hydrolytic degradation under physiological conditions (PBS, pH 7.4, 37 °C) and (iii) enzymatic degradation using lipases (*Candida antartica*, PBS, pH 7.4, 37 °C) (Supplementary Table 3).

Owing to their higher proportions in ring-opened BMDO than their MPDL-containing counterparts at similar compositions, degradation of P(AAm-*co*-BMDO) copolymers under accelerated conditions was much more pronounced, as shown by the significant shifts of the SEC traces toward lower *M*_n_ even for copolymers with low BMDO contents (Table 2 and Supplementary Figs. 16 and 17). Indeed, 1.7 mol.% BMDO already led to 45% decrease in *M*_n_ (**P10**), and very high degradations up to 90% decrease in *M*_n_ were observed for higher *F*_BMDO_ values, with a rather good agreement with the predicted values. ^1^H NMR showed the nearly complete disappearance of the peak characteristics of proton *h* at 4.9–5.2 ppm in the alpha position to the ester group of BMDO, thus confirming the cleavage of the ester groups (Supplementary Fig. 18).

**Fig. 6:**
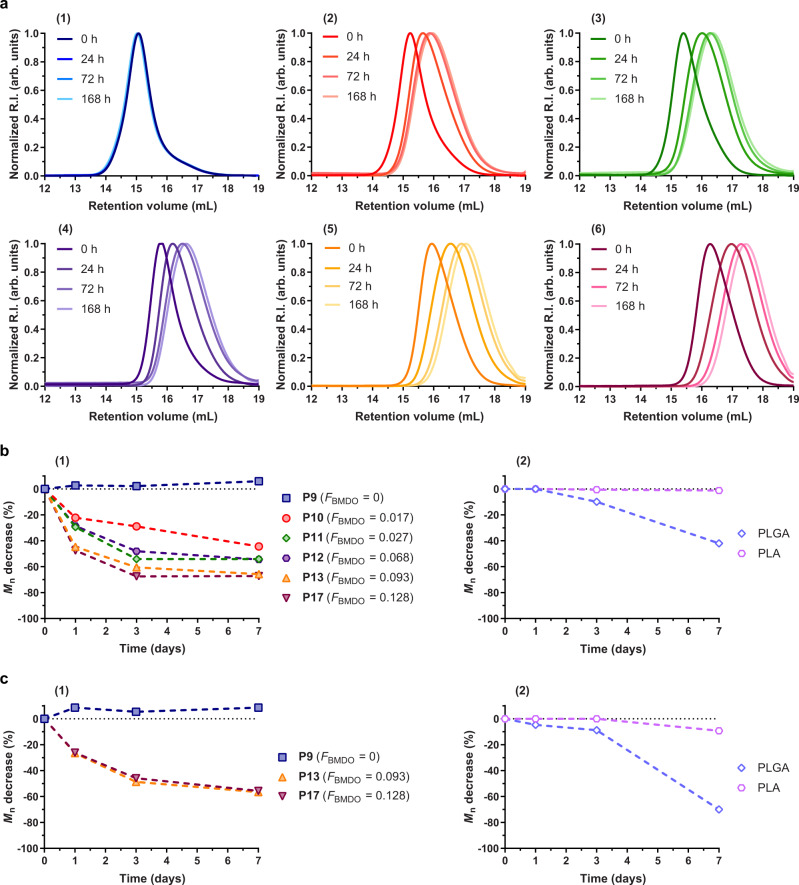
Hydrolytic degradation in physiological conditions of P(AAm-*co*-BMDO) copolymers, PLA, and PLGA. **a** Evolution of the SEC chromatograms at different time during hydrolytic degradation in PBS (pH 7.4, 37 °C) of P(AAm-*co*-BMDO) copolymers (Table 2, **P9**–**P13** and **P17**) as a function *F*_BMDO_: (1) **P9** (*F*_BMDO_ = 0, no *T*_cp_); (2) **P10** (*F*_BMDO_ = 0.017, no *T*_cp_); (3) **P11** (*F*_BMDO_ = 0.027, no *T*_cp_); (4) **P12** (*F*_BMDO_ = 0.068, no *T*_cp_); (5) **P13** (*F*_BMDO_ = 0.093, *T*_cp_ = 25 °C) and (6) **P17** (*F*_BMDO_ = 0.128, *T*_cp_ = 52 °C). **b** Evolution of the number-average molar mass, *M*_n_, with time during hydrolytic degradation in physiological conditions (PBS, pH 7.4, T = 37 °C) of: (1) PAAm (**P9**), P(AAm-*co*-BMDO) copolymers **P10**–**P13** and **P17** and (2) PLA and PLGA; **c** evolution of the *M*_n_ with time during enzymatic degradation with lipases (*Candida antartica*, PBS, pH 7.4, T = 37 °C) of: (1) PAAm (**P9**), P(AAm-*co*-BMDO) copolymers **P13** and **P17** and (2) PLA and PLGA.

Remarkably, P(AAm-*co*-BMDO) copolymers all exhibited extremely fast hydrolytic degradation in physiological conditions, whether they were fully water-soluble (**P10**–**P12**), or thermosensitive with a UCST below (**P13**) or above 37 °C (**P17**). The decrease in *M*_n_, which was only governed by the BMDO content, was in the 21–58% range after only 24 h and in the 43–67% range after 7 days (Fig. 6a, b). Homogeneity of the degradation products was also demonstrated by the homogeneous shifts of the SEC chromatograms and the constant dispersity of the degradation products with time, except for **P8** which exhibits the highest amount of MDO (*F*_MDO_ = 0.44) (Supplementary Fig. 19). Not only these degradation kinetics were much faster than those of any CKA-containing vinyl copolymers reported so far (see Supplementary Fig. 20 for comparison with the literature), but they were also faster than those of traditional aliphatic polyesters like PLA and even PLGA under the same conditions (Fig. 6b and Supplementary Fig. 21), which is unprecedented for vinyl copolymers. Considering that other CKA-containing hydrophilic copolymers (e.g., P(OEGMA-*co*-MPDL), P(GMA-*co*-BMDO))^19,20^ exhibit much slower hydrolytic degradation under physiological conditions than the AAm-counterparts, this highlights the crucial role of AAm in the very fast hydrolytic degradation of P(AAm-*co*-CKA) copolymers. This is likely explained by very high hydrophilicity of AAm moieties leading to optimal water uptake and efficient solvation of ester groups. These results represent an important achievement as, for the first time, a copolymerization system allowed to circumvent the very slow hydrolytic degradation in physiological conditions of CKA-containing vinyl copolymers and to surpass the hydrolytic degradation of traditional aliphatic polyesters. It is also interesting to note that the degradation was also successfully carried out in deionized water (pH ~5.5) and resulted in a decrease in *M*_n_ of almost 40% for **P17** after only 3 days (Supplementary Fig. 22). This fast degradation kinetics was also observed by UV measurements during five consecutive cooling and heating cycles of P(AAm-*co*-BMDO) copolymer **P13** solution in water performed over a period of 7 h (Supplementary Fig. 23). The cloud point measured was notably affected from the first cycle to the fifth, as evidenced by a decrease of ~1–2 °C after each cycle, likely because of the on-going degradation of the copolymer. Interestingly, such tuning of the UCST transition during degradation is an interesting additional feature that had been shown on vinyl copolymers containing disulfide^30^ or thioesters^34^ linkages, but never before on CKA-based polymers.

Interestingly, despite their lack of thermosensitivity, P(AAm-*co*-MDO) copolymers also exhibited rapid hydrolytic degradation in physiological conditions, whatever they were water-soluble (**P6**) or insoluble in water (**P8**) (Supplementary Figs. 24 and 25). Their degradation kinetics were similar to that of P(AAm-*co*-BMDO) copolymers with decrease in *M*_n_ in the 65–75% range after 7 days. These results, therefore, broadened the application scope of the AAm/CKA copolymerization system as depending on the nature of the CKA and of its content, well-defined water-soluble, insoluble, or UCST copolymers with rapid hydrolytic degradation in physiological conditions/water can be readily synthesized.

PAAm, P(AAm-*co*-BMDO) copolymers **P13** and **P17**, and P(AAm-*co*-MDO) copolymers **P6** and **P8** were also subjected to enzymatic degradation in PBS at 37 °C in the presence of lipases from *Candida antartica*, a subclass of esterases. While the *M*_n_ of PAAm stayed constant over time, degradation kinetics of **P13** and **P17** were rather similar to those performed under physiological conditions and reached ~60% after 7 days (Fig. 6c and Supplementary Fig. 26). We nonetheless suspected that, given the rapid hydrolytic degradation in PBS of these copolymers, it was difficult to determine the contribution of the enzymes and that the decrease in *M*_n_ in Fig. 6c mainly reflected the contribution of hydrolytic degradation. As for MDO-containing copolymers, **P8** was more rapidly degraded enzymatically than hydrolytically (especially within the first 3 days), likely owing to its high MDO content, whereas the opposite trend was obtained for **P6** (Supplementary Figs. 27 and 28).

### In vitro cytotoxicity

**Fig. 7:**
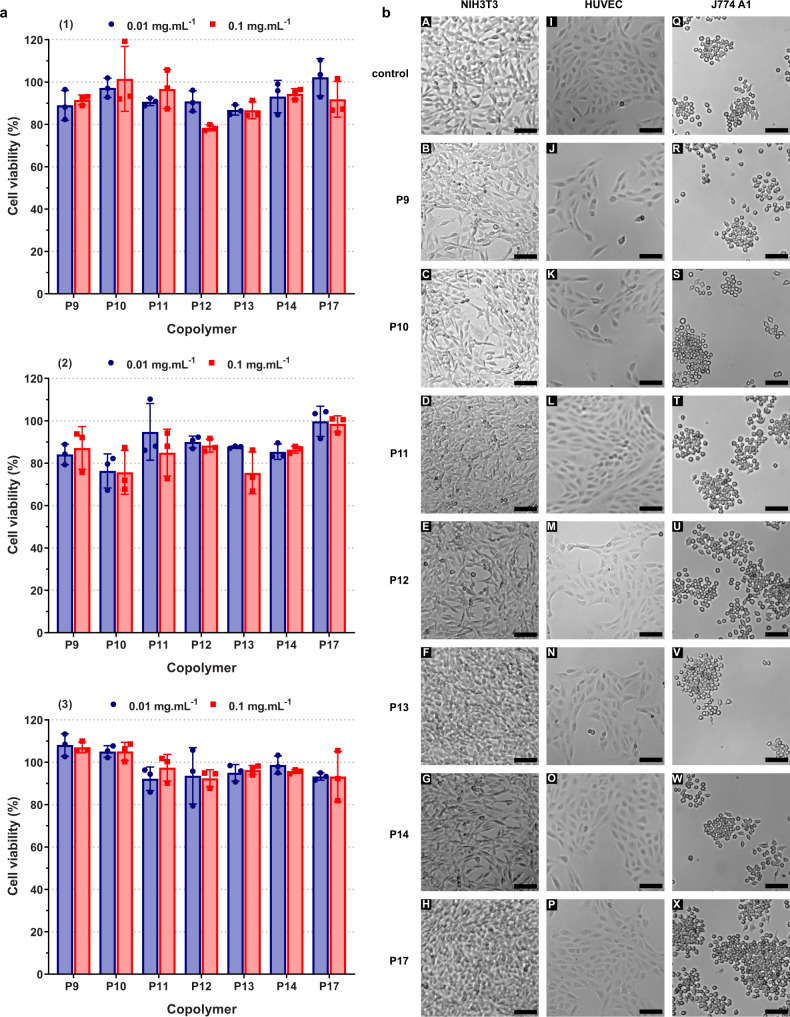
Cytocompatibility of P(AAm-*co*-BMDO) copolymers. **a** Cell viability (MTT assay) after incubation of: (1) NIH/3T3 cells; (2) HUVEC cells; and (3) J774.A1 cells with P(AAm-*co*-BMDO) copolymers as function of the BMDO content (**P9**–**P14** and **P17**, Table 2) at 0.01 and 0.1 mg mL^−1^. Results were expressed as the percentage of absorption of treated cells (*n* = 3 for each condition, error bars represent mean ± SD) in comparison with the values obtained from untreated control cells; **b** optical images of NIH/3T3 cells (first column, **a**–**h**), HUVEC cells (second column, I–P) and J774.A1 cells (third column, Q–X) taken by optical microscopy after treatment for 72 h with P(AAm-*co*-BMDO) copolymers (0.1 mg mL^−1^): Line 1: untreated cells; line 2: **P9**; line 3: **P10**; line 4: **P11**; line 5: **P12**; line 6: **P13**; line 7: **P14;** line 8: **P17**. Scale bar = 100 μm.

Recognizing that it is essential to evaluate the safety of developed materials prior to any biopharmaceutical use, a small library of water-soluble or UCST, BMDO-containing copolymers with variable BMDO content (**P9**–**P14** and **P17**, *F*_BMDO_ = 0–0.128, Table 2) and *M*_n_ (**P13** and **P18**–**P19**, *M*_n, exp_ = 7.6–10.2 kg mol^−1^, Supplementary Table 2) was tested on three representative healthy cell lines to investigate any cytotoxic effects on the basis of cell viability assays and cell morphology observations (Fig. 7 and Supplementary Figs. 29–32). The cell lines tested were: (i) murine fibroblasts (NIH/3T3), which are one of the most commonly used fibroblast cell lines; (ii) human umbilical vein endothelial cells (HUVEC), which are highly sensitive and give rapid response to external stimuli and (iii) murine macrophages (J774.A1), which are typical monocyte cells that play an important role in phagocytosis^45,46^.

Overall, all copolymers tested showed high cell viabilities (75–100%) by MTT(3-[4,5-dimethylthiazol-2-yl]-2,5 diphenyl tetrazolium bromide) assay on the three cell lines at 0.01 and 0.1 mg mL^−1^ (Fig. 7a and Supplementary Fig. 29). Also, no clear trend was observed as neither the presence nor absence of a UCST transition, nor the BMDO content, nor the range of *DP*_n, th_ tested seemed to influence the cell viability. Furthermore, varying the transition temperature over a wide range of values (*T*_cp_ = 12–52 °C) also did not affect the cytotoxicity. It is also worth mentioning that the 72 h-incubation time with cells (to allow at least two cell doubling times to perform relevant MTT assays) combined with the rapid hydrolytic degradation of the copolymers, suggests that neither the starting copolymers nor their degradation products were toxic to the cells. This point is key as it represents an important indication concerning the ultimate biocompatibility of the copolymers.

Cellular morphology observations after a 72 h-incubation with the different copolymers confirmed the cell viability results as no evidence of toxic effects were noticed compared to untreated cells or cells treated with unmodified PAAm (Fig. 7b and Supplementary Figs. 30–32). Indeed, no difference in size, shape, cell density, or cell proliferation was observed whatever the copolymer, its concentration, or the cell line tested.

This cytotoxicity study, therefore, suggests that PAAm chemically modified by introducing BMDO units into its backbone did not adversely affect the cell viability and the morphology of three representative healthy cell lines.

### Synthesis of block copolymers and application to doubly thermosensitive PEGylated nanoparticles

To illustrate the versatility of this copolymerization system, P(AAm-*co*-BMDO) copolymer **P13** was first successfully chain extended with AAm via RAFT polymerization at 70 °C for 2 h to produce a P(AAm-*co*-BMDO)-*b*-PAAm diblock copolymer (*M*_n, exp_ = 18.2 kg mol^−1^, *Đ* = 1.6). Despite slight broadening of the dispersity, gradual shift of the SEC traces toward higher molecular weights was shown. Its sacrificial P(AAm-*co*-BMDO) block was then partially degraded under hydrolytic conditions to yield a lower *M*_n_ copolymer (*M*_n, exp_ = 16.1 kg mol^−1^, *Đ* = 1.5) as shown by SEC (Supplementary Fig. 33).

**Fig. 8:**
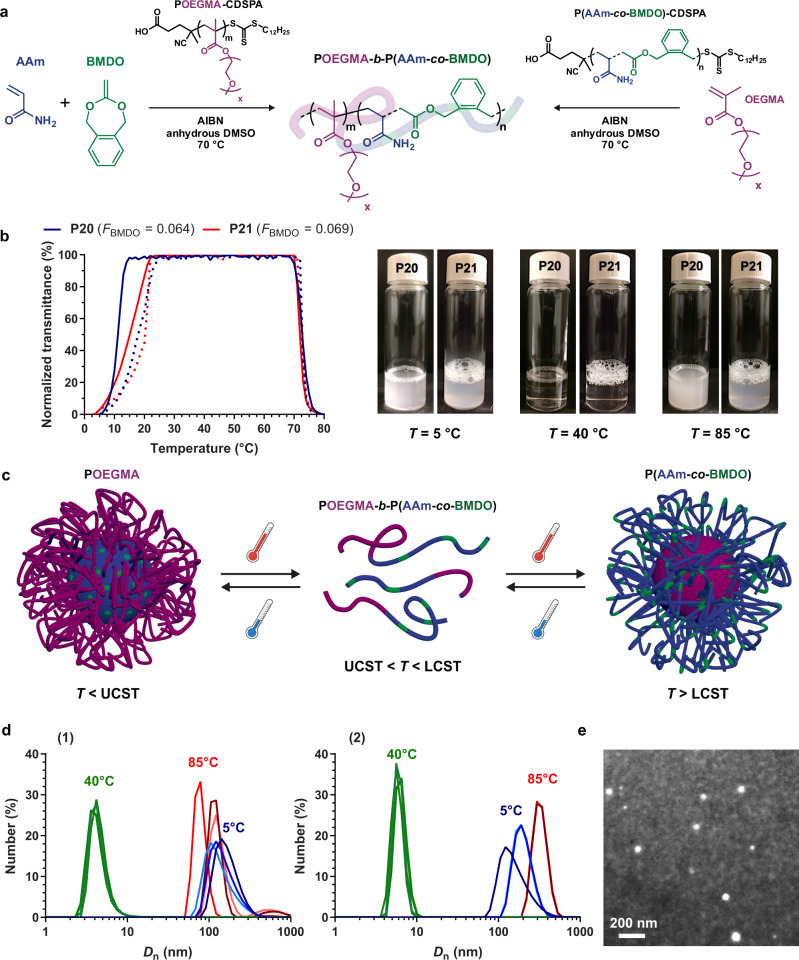
Application to amphiphilic POEGMA-*b*-P(AAm-*co*-BMDO) diblock copolymers. **a** Synthesis of amphiphilic POEGMA-*b*-P(AAm-*co*-BMDO) (**P20**) and P(AAm-*co*-BMDO)-*b*-POEGMA (**P21**) diblock copolymers via RAFT polymerization at 70 °C in anhydrous DMSO using POEGMA or P(AAm-*co*-BMDO) as macro-CTA, respectively; **b** variation of the solution transmittance vs. temperature and associated pictures of the doubly thermoresponsive P(AAm-*co*-BMDO)-*b*-POEGMA diblock copolymers **P20**–**P21** solution in water at 10 mg mL^−1^ (1 °C min^−1^, solid and dotted lines for cooling and heating, respectively); **c** schematic representation of the doubly thermoresponsive P(AAm-*co*-BMDO)-*b*-POEGMA diblock copolymer nanoparticles morphology upon temperature switch; **d** evolution of the number-average diameter (*D*_n_) of the doubly thermoresponsive P(AAm-*co*-BMDO)-*b*-POEGMA diblock copolymer nanoparticles **P20** (1) and **P21** (2) at 1.67 mg mL^−1^ at *T* = 40 °C, 5 °C, and 85 °C. **e** Representative TEM image with negative staining of nanoparticles **P20** (see Supplementary Figs. 39 and 40 for other TEM images and particle size distribution, respectively). This experiment was repeated three times with similar results.

Macromolecular engineering was then further extended to the synthesis of P(AAm-*co*-BMDO)-*b*-POEGMA amphiphilic diblock copolymers for application in the formulation of degradable, PEGylated, UCST nanoparticles for drug delivery purposes. To determine the most efficient synthetic pathway, such copolymers were achieved by RAFT in anhydrous DMSO at 70 °C by either: (i) chain extension of a POEGMA macro RAFT agent (*M*_n, exp_ = 7400 g mol^−1^, *Đ* = 1.1) by a 50:50 mixture of AAm and BMDO (**P20**) or (ii) chain extension of a P(AAm-*co*-BMDO) macro-RAFT agent (*M*_n, exp_ = 4800 g mol^−1^, *Đ* = 1.5, *F*_BMDO_ = 0.069) by OEGMA (**P21**) (Supplementary Table 4 and Fig. 8a). In both cases, the desired diblock copolymers were obtained with *F*_BMDO_ ~0.06 and >86% open BMDO, as assessed by ^1^H NMR and SEC, together with better control (*Đ* = 1.3 vs 1.9) when starting from a P(AAm-*co*-BMDO) macro-RAFT agent (**P21**) (Supplementary Figs. 34 and 35). As observed by transmittance measurements (Supplementary Table 4), both copolymers successfully maintained their UCST properties with a little shift of the *T*_cp_ values toward lower values close to ambient temperature (T_cp_ ~17–20 °C) in comparison with a similar P(AAm-*co*-BMDO) copolymer (**P14**). This is likely due to the influence of the water-soluble POEGMA block, as already seen with other systems^44^. Interestingly, the presence of the POEGMA block also conferred an LCST transition to the copolymer at ~73 °C (Fig. 8b), and the three different solubility states can be reversibly reached upon temperature oscillation (Supplementary Movie 1).

By taking advantage of the UCST feature of the diblock copolymers, their formulation into well-defined, degradable nanoparticles was achieved through an organic-solvent-free, all-water nanoprecipitation process in the presence of 0.1 wt.% Pluronic as a surfactant. It consisted in adding an aqueous solution of P(AAm-*co*-BMDO)-*b*-POEGMA copolymer prepared at 25 °C (*T* > UCST) into an aqueous solution at 5 °C (*T* < UCST) under stirring (Supplementary Fig. 36). This nanoprecipitation procedure can therefore operate entirely in water, which is very advantageous compared to the classical nanoprecipitation technique that requires the use of a water-miscible organic solvent (e.g., acetone, tetrahydrofuran (THF), etc.), whose removal may often be incomplete. DLS measurements revealed that well-defined nanoparticles were obtained in both cases with average diameters of ~200 nm and narrow particle size distributions (Supplementary Table 4). The nanoparticles’ formation and colloidal characteristics were shown to be reversible upon oscillating the temperature between 5 °C (insoluble state, *T* < UCST, nanoparticles with P(AAm-*co*-BMDO) core), 40 °C (soluble state, UCST < *T* < LCST) and 85 °C (insoluble state, *T* > LCST, nanoparticles with POEGMA core) up to at least three consecutive cycles (Fig. 8c, d and Supplementary Figs. 37 and 38), thus leading to degradable, doubly thermosensitive^47,48^ copolymer nanoparticles. These variations in number-, intensity- and volume-average diameters (Supplementary Figs. 37 and 38), confirmed the different transitions observed by UV measurements. Transmission electron microscopy (TEM) analysis confirmed the formation of spherical and narrowly dispersed nanoparticles (Fig. 8e and Supplementary Figs. 39 and 40), exhibiting diameters in the 60-80 nm range, which correspond to the diameters in the dry state.

The evolution of their colloidal properties was monitored by DLS upon storage at 37 °C and 4 °C (Supplementary Fig. 41). A sharp decrease in average diameter from 180 nm down to 50 nm was obtained after 3 days at body temperature, thus attesting for their disintegration (likely via concomitant disassembly and hydrolytic degradation). On the other hand, at 4 °C, their average diameter remained almost constant during storage, due to a slowing down of the degradation process (but not its stopping as shown by the increase of the particle size distribution).

To expand the field of possibilities in terms of applications, the UCST transition was also adjusted close to body temperature simply by tuning the POEGMA chain length and the composition of the P(AAm-*co*-BMDO) block. This was achieved by targeting a shorter POEGMA macro-CTA (*M*_n, exp_ = 3200 g mol^−1^, *Đ* = 1.33), in order to promote higher UCST values, followed by chain extension with AAm/BMDO (*f*_BMDO,0_ = 0.55) to yield a final *M*_n, exp_ = 7700 g mol^−1^; *Đ* = 1.5, with *F*_BMDO_ = 0.126 (Supplementary Table 5). This copolymer (**P22**) exhibited a UCST transition in the 40–42 °C range as shown by transmittance and DLS measurements (Supplementary Fig. 42 and 43), which could be relevant for mild hyperthermia therapy. Interestingly, a progressive shift of its *T*_cp_ to lower values during degradation under physiological conditions was observed until 16 days when it reached 3 °C (Supplementary Fig. 44).

## Discussion

In this study, we discovered that the RAFT-mediated rROP of AAm with different CKAs (MPDL, BMDO and MDO) represented a straightforward route toward the synthesis of a broad range of mostly well-defined P(AAm-*co*-CKA) copolymers that exhibited fast and tunable degradation in physiological conditions (PBS, pH 7.4, 37 °C), that even surpassed that of PLA and even PLGA in the same conditions. With aromatic ring-containing-CKAs, the resultant copolymers even showed tunable and sharp UCST transitions in the ~10–50 °C range, which could be of high interest for drug delivery applications. The UCST transition could be readily and independently adjusted by modifying the CKA content in the copolymer, the copolymer concentration, and the targeted average degree of polymerization. Since the copolymerization was successfully applied to both MDO and MPDL/BMDO, the degradation can therefore be decoupled from the thermosensitivity, which is also advantageous depending on the targeted application. Moreover, preliminary cell viability assays showed that they had a good cytocompatibility on three different representative healthy cell lines, representing a key result considering biomedical applications.

To demonstrate the robustness and the broad applicability of this synthetic approach, POEGMA-*b*-P(AAm-*co*-CKA) diblock copolymers were synthesized, whose UCST allowed their formulation by an innovative all-water nanoprecipitation process, that avoided the use of organic solvent, into narrowly dispersed nanoparticles of ~200 nm in diameter suitable for drug delivery applications. Owing to the LCST of the POEGMA block, these nanoparticles even exhibited both UCST and LCST transitions, and can be considered as the first example of doubly thermosensitive, degradable nanoparticles.

Considering all its important features and advantages, we believe that this two-in-one copolymerization system, in which the CKA confers both efficient degradability and thermosensitivity, could lead to very useful macromolecular building blocks that would greatly expand the current and somewhat limited arsenal of degradable materials. It can therefore offer exciting perspectives for a wide range of different applications ranging from degradation of hydrophilic plastic-based materials such as PAAm which is a key polymer in this field^49^, to hyperthermia-mediated drug delivery and tissue engineering.

## Methods

### Materials

Cyclic ketene acetals MPDL, BMDO, and MDO were prepared using cyclic bromoacetals as intermediates^50^. CDSPA (97%), AAm (≥99%), AIBN (98%), anhydrous DMSO (≥99.9%), anhydrous acetonitrile (≥99.9%), Dubbelco’s Phosphate Buffer Saline (PBS) suitable for cell culture, lipases B from *Candida antartica* immobilized on Immobead 150 (4584 U g^−1^), OEGMA (*M*_n_ = 300 g mol^−1^), poly(d,l-lactide-*co*-glycolide) acid terminated (Resomer^®^ RG 502 H, PLGA, *M*_w_ = 7000–17,000 g mol^−1^), poly(d,l-lactide) acid terminated (Resomer^®^ R 202 S, PLA, *M*_w_ = 10,000–18,000 g mol^−1^) and KOH (90%) were purchased from Sigma-Aldrich. Methanol (HPLC analytical grade) and diethyl ether (HPLC analytical grade) were purchased from Carlo Erba. THF (HPLC grade) was purchased from VWR Chemicals. DMSO-d_6_ was purchased from Eurisotop. All reagents and solvents were used as received except AIBN which was purified by recrystallization in methanol and AAm which was recrystallized in chloroform.

### Analytical methods

#### NMR spectroscopy

NMR spectroscopy was performed in 5 mm diameter tubes in DMSO-d_6_ at 25 °C. ^1^H-NMR spectroscopy was performed on a Bruker Avance 300 spectrometer at 300 MHz. The chemical shift scale was calibrated based on the internal solvent signals (*δ* = 2.50 ppm for DMSO-d_6_). Data were processed with MestReNova 11.0.4 software.

#### SEC

SEC was performed at 60 °C using two columns in series from Agilent Technologies (PL PolarGel-M, 300 × 7.5 mm; bead diameter 8 µm; molar mass range 1000–5,00,000 g mol^−1^) preceded by a guard column from Agilent Technologies (PL PolarGel-M, 7.5 × 50 mm; bead diameter 8 µm) and a triple detection system (Viscotek TDA/GPCmax from Malvern) with a differential refractive index detector, low and right-angle light scattering detectors and a differential viscometer detector. The eluent was DMSO with 100 mM LiBr and 0.36 wt.% of 2,6-di-*tert*-butyl-4-methylphenol (BHT) as a marker at a flow rate of 0.7 mL min^−1^. The system was calibrated using poly(methyl methacrylate) (PMMA) standards (peak molar masses, *M*_p_ = 540–342,900 g mol^−1^) from Agilent Technologies. This allowed the determination of the number-average molar mass (*M*_n_), the weight-average molar mass (*M*_w_) and the dispersity (*Ð* = *M*_w_/*M*_n_). All samples were filtered over 0.22 µm PTFE filters prior to injection. Data were collected and processed with OmniSEC 4.0 software.

#### UV-Vis spectroscopy

Light transmittance (%) of samples was measured using a Lambda 25 UV/VIS spectrometer equipped with a PTP 1 + 1 Peltier system for temperature control (PerkinElmer), at a wavelength of 500 nm, a cell path length of 10 mm and under magnetic stirring. Samples were prepared at 10 mg mL^−1^ in deionized water and placed in a quartz cuvette. The measurements were carried out by first cooling the solution from the used highest temperature (T >> UCST) at a constant rate of 1 °C min^−1^ followed by reheating the solution back to the starting temperature at the same rate. The inflection point of the transmittance curve was considered as the UCST cloud point. It was graphically determined by the maximum of the first derivative of the cooling/heating curves. Data were collected with Winlab 6.0.3.0730 software and processed with Data Processor and Viewer (DPV) 1.00.100.0010 software.

#### DLS

Nanoparticles’ intensity average diameters (*D*_z_) and particle size distributions were measured by DLS with a Nano ZS from Malvern equipped with a 4 mW He−Ne laser (633 nm wavelength) at a fixed scattering angle of 173°. Similar to UV-Vis measurements, samples were prepared at 10 mg mL^−1^ and placed in a quartz cuvette. The cooling/heating measurements were taken at an interval of 1 °C starting from T >> UCST and the solution was equilibrated at each temperature for 60 s prior to the measurements. The inflection point of the intensity average diameters curve was considered the cloud point (UCST). It was graphically determined by the maximum of the first derivative of the cooling/heating curves. Data were processed with Malvern-Zetasizer 7.12 software.

#### Optical microscopy

Optical images of copolymer solution in water were captured with a Leitz Diaplan microscope equipped with a Coolsnap ES camera (Roper Scientific). The samples for optical microscopy were prepared by placing a drop of the copolymer solution (10 mg mL^−1^) at *T* < UCST and *T* > UCST on a glass microscope slide prior to examination. Data were collected and processed with RS Image V1.9.2 software.

#### TEM

Grids were glowing and discharged before use. 5 μL of nanoparticle suspensions (1.67 mg mL^−1^) kept at 4 °C were deposited for 5 min on copper grids covered with formvar-carbon film. The excess solution was blotted off using filter paper. Samples were then stained for 30 s using phosphotungstic acid (2%, w/v) also stored at 4 °C. Then the excess solution was removed using filter paper. The grids were then analyzed using a JEOL JEM-1400 operating at 80 kV. Images were acquired using an Orius camera (Gatan Inc, USA). Data were collected and processed with Zen 2.6 (blue edition) software. Sizes were measured by ImageJ 1.53a.

### Synthetic procedures

#### Synthesis of poly(acrylamide-co-2-methylene-4-phenyl-1,3-dioxolane) (P(AAm-co-MPDL), **P0–P4**)

A typical procedure (**P2**, Table 1) is as follows: in a 40 mL vial, fitted with a rubber septum and a magnetic stirring bar, a mixture of AAm (120 eq., 4.8 mmol, 0.34 g) and MPDL (*f*_MPDL,0_ = 0.4, 80 eq., 3.2 mmol, 0.52 g) (total mole = 8 mmol), CDSPA (1 eq., 0.04 mmol, 16.1 mg) and AIBN (0.6 eq., 0.024 mmol, 3.9 mg) was dissolved in anhydrous DMSO (1 mL). The solution was bubbled with dry argon to remove dissolved oxygen for 15 min at room temperature and then immersed in a preheated oil bath at 70 °C for 16 h. The solution was then rapidly cooled under air. The copolymer was then precipitated thrice in cold methanol as follows: the reaction mixture was first added dropwise in 50 mL cold methanol and centrifuged at 15,000 × *g* at 10 °C for 10 min. After discarding the liquid fraction, 50 mL of cold methanol was added, and the copolymer was suspended in a sonic bath. The suspension was centrifuged again, and the procedure was repeated one more time. The P(AAm-*co*-MPDL) obtained was then dried under high vacuum until constant weight. The same procedure was adapted as follows for **P0** [*f*_MPDL,0_ = 0, AAm (200 eq., 8 mmol, 0.57 g)], **P1** [*f*_MPDL,0_ = 0.2, MPDL (40 eq., 1.6 mmol, 0.26 g), AAm (160 eq., 6.4 mmol, 0.45 g)], **P3** [*f*_MPDL,0_ = 0.6, MPDL (120 eq., 4.8 mmol, 0.78 g), AAm (80 eq., 3.2 mmol, 0.23 g)] and **P4** [*f*_MPDL,0_ = 0.8, MPDL (160 eq., 6.4 mmol, 1.04 g), AAm (40 eq., 1.6 mmol, 0.11 g)].

#### Synthesis of poly(acrylamide-co-2-methylene-1,3-dioxepane) (P(AAm-co-MDO), **P5–P8**)

A typical procedure (**P6**, Table 1) is as follows: in a 40 mL vial, fitted with a rubber septum and a magnetic stirring bar, a mixture of AAm (120 eq., 4.8 mmol, 0.34 g) and MDO (80 eq., 3.2 mmol, 0.37 g) (total mole = 8 mmol), CDSPA (1 eq., 0.04 mmol, 16.1 mg) and AIBN (0.6 eq., 0.024 mmol, 3.9 mg) was dissolved in anhydrous DMSO (1 mL). The solution was bubbled with dry argon to remove dissolved oxygen for 15 min at room temperature and then immersed in a preheated oil bath at 70 °C for 16 h. The solution was then rapidly cooled under air. The copolymer was then precipitated thrice in cold methanol using similar procedure as describe above for the synthesis of **P0**–**P4**. The same procedure was adapted as follows for **P5** [*f*_MDO,0_ = 0.2, MDO (40 eq., 1.6 mmol, 0.18 g), AAm (160 eq., 6.4 mmol, 0.45 g)], **P7** [*f*_MDO,0_ = 0.6, MDO (120 eq., 4.8 mmol, 0.55 g), AAm (80 eq., 3.2 mmol, 0.23 g)] and **P8** [*f*_MDO,0_ = 0.8, MDO (160 eq., 6.4 mmol, 0.73 g), AAm (40 eq., 1.6 mmol, 0.11 g)].

#### Synthesis of poly(acrylamide-co-5,6-benzo-2-methylene-1,3-dioxepane) (P(AAm-co-BMDO), **P9–P19**)

A typical procedure (**P13**, Table 2) is as follows: in a 40 mL vial, fitted with a rubber septum and a magnetic stirring bar, a mixture of AAm (120 eq., 4.8 mmol, 0.34 g) and BMDO (80 eq., 3.2 mmol, 0.52 g) (total mole = 8 mmol), CDSPA (1 eq., 0.04 mmol, 16.1 mg) and AIBN (0.6 eq., 0.024 mmol, 3.9 mg) was dissolved in anhydrous DMSO (10 mL). The solution was bubbled with dry argon to remove dissolved oxygen for 15 min at room temperature and then immersed in a preheated oil bath at 70 °C for 16 h. The solution was then rapidly cooled under air. The copolymer was then precipitated thrice in cold methanol using similar procedure as describe above for the synthesis of **P0**–**P4**. The P(AAm-*co*-BMDO) obtained was then dried under high vacuum until constant weight. The same procedure was adapted as follows for **P9** [*f*_BMDO,0_ = 0, AAm (200 eq., 8 mmol, 0.57 g)], **P10** [*f*_BMDO,0_ = 0.2, BMDO (40 eq., 1.6 mmol, 0.26 g), AAm (160 eq., 6.4 mmol, 0.45 g)], **P11** [*f*_BMDO,0_ = 0.3, BMDO (60 eq., 2.4 mmol, 0.39 g), AAm (140 eq., 5.6 mmol, 0.4 g)], **P12** [*f*_BMDO,0_ = 0.35, BMDO (70 eq., 2.8 mmol, 0.45 g), AAm (130 eq., 5.2 mmol, 0.37 g)], **P14** [*f*_BMDO,0_ = 0.5, BMDO (100 eq., 4 mmol, 0.65 g), AAm (100 eq., 4 mmol, 0.28 g)], **P15** [*f*_BMDO,0_ = 0.51, BMDO (102 eq., 4.1 mmol, 0.66 g), AAm (98 eq., 3.9 mmol, 0.28 g)], **P16** [*f*_BMDO,0_ = 0.53, BMDO (106 eq., 4.2 mmol, 0.69 g), AAm (94 eq., 3.8 mmol, 0.27 g)] and **P17** [*f*_BMDO,0_ = 0.55, BMDO (110 eq., 4.4 mmol, 0.71 g), AAm (90 eq., 3.6 mmol, 0.26 g)]. Note: **P9**–**P13** were precipitated in cold methanol; **P14**–**P16** were precipitated in cold THF and **P17** was precipitated in cold diethyl ether.

The same procedure was also adapted as follows for **P18** [*f*_BMDO,0_ = 0.4, BMDO (160 eq., 3.2 mmol, 0.52 g), AAm (240 eq., 4.8 mmol, 0.34 g), CDSPA (1 eq., 0.02 mmol, 8.1 mg) and AIBN (0.6 eq., 0.012 mmol, 2 mg)] and **P19** [*f*_BMDO,0_ = 0.4, BMDO (240 eq., 3.2 mmol, 0.52 g), AAm (360 eq., 4.8 mmol, 0.34 g), CDSPA (1 eq., 0.013 mmol, 5.4 mg) and AIBN (0.6 eq., 0.008 mmol, 1.3 mg)].

#### Synthesis of poly[(acrylamide-co-5,6-benzo-2-methylene-1,3-dioxepane)]-b-polyacrylamide (P(AAm-co-BMDO)-b-PAAm))

P(AAm-*co*-BMDO) **P13** (Table 2, *M*_n, exp_ = 7600 g mol^−1^, *Đ* = 1.4, *F*_BMDO_ = 0.093) was chain extended with AAm as follows: in a 40 mL vial, fitted with a rubber septum and a magnetic stirring bar, a mixture of AAm (200 eq., 8.0 mmol, 0.57 g), P(AAm-*co*-BMDO) **P13** (1 eq., 0.04 mmol, 0.3 g) and AIBN (0.6 eq., 0.024 mmol, 3.9 mg) was dissolved in anhydrous DMSO (10 mL). The solution was bubbled with dry argon to remove dissolved oxygen for 15 min at room temperature and then immersed in a preheated oil bath at 70 °C for 2 h. The solution was then rapidly cooled under air. The copolymer was then precipitated thrice in cold methanol using similar procedure as describe above for the synthesis of **P0**–**P4**. The P(AAm-*co*-BMDO)-*b*-PAAm obtained was then dried under high vacuum until constant weight.

#### Synthesis of poly[oligo(ethylene glycol) methyl ether methacrylate] (POEGMA) macro-CTA

A typical synthesis of POEGMA_23_ macro-CTA was conducted as follows: in a pre-dried 50 mL round bottom flask, fitted with a rubber septum and a magnetic stir bar, a mixture of OEGMA (3.68 g, 0.012 mol), CDSPA (1 eq., 0.24 mmol, 0.097 g) and AIBN (0.25 eq., 0.059 mmol, 9.6 mg) was dissolved in anhydrous acetonitrile (25 mL). The solution was bubbled with dry argon to remove dissolved oxygen for 15 min at room temperature and then immersed in a preheated oil bath at 70 °C for 5 h. The solution was then rapidly cooled under air. Acetonitrile was removed and the resulting polymer solution was precipitated once in excess of cold mixture of 1:1 of diethyl ether and petroleum spirit. The POEGMA_23_ macro-CTA (*M*_n, exp_ = 7400 g mol^−1^, *Đ* = 1.1) obtained was then dried under a high vacuum until constant weight.

#### Synthesis of poly[(oligo(ethylene glycol) methyl ether methacrylate]-b-poly(acrylamide-co-5,6-benzo-2-methylene-1,3-dioxepane) (POEGMA-b-P(AAm-co-BMDO), **P20**)

POEGMA_23_ (*M*_n, exp_ = 7400 g mol^−1^, *Đ* = 1.1) was used as a macro-RAFT agent to polymerize AAm and BMDO (*f*_BMDO,0_ = 0.5) and yield POEGMA-*b*-P(AAm-*co*-BMDO) diblock copolymer (**P20**, Supplementary Table 4). In a 40 mL vial, fitted with a rubber septum and a magnetic stirring bar, a mixture of AAm (100 eq., 4.0 mmol, 0.28 g) and BMDO (100 eq., 4.0 mmol, 0.65 g) (total mole = 8 mmol), POEGMA_23_ macro-CTA (1 eq., 0.04 mmol, 0.64 g) and AIBN (0.6 eq., 0.024 mmol, 3.9 mg) was dissolved in anhydrous DMSO (10 mL). The solution was bubbled with dry argon to remove dissolved oxygen for 15 min at room temperature and then immersed in a preheated oil bath at 70 °C for 16 h. The solution was then rapidly cooled under air. The copolymer was then precipitated thrice in cold THF using similar procedure as describe above for the synthesis of **P0**–**P4**. POEGMA-*b*-P(AAm-*co*-BMDO) obtained was then dried under high vacuum until constant weight. An identical procedure was followed for the synthesis of **P22** with some modifications: POEGMA (*M*_n, exp_ = 3200 g mol^−1^, Đ = 1.33) was used as a macro-RAFT agent to polymerize AAm and BMDO (*f*_BMDO,0_ = 0.55) and yield POEGMA-*b*-P(AAm-*co*-BMDO) diblock copolymer (**P22**, Supplementary Table 5). In a 40 mL vial, fitted with a rubber septum and a magnetic stirring bar, a mixture of AAm (90 eq., 3.6 mmol, 0.256 g) and BMDO (110 eq., 4.4 mmol, 0.714 g) (total mole = 8 mmol), POEGMA macro-CTA (1 eq., 0.04 mmol, 0.128 g) and AIBN (0.6 eq., 0.024 mmol, 3.9 mg) was dissolved in anhydrous DMSO (10 mL). The solution was bubbled with dry argon to remove dissolved oxygen for 15 min at room temperature and then immersed in a preheated oil bath at 70 °C for 16 h. The solution was then rapidly cooled under air. The copolymer was then precipitated thrice in cold diethyl ether and then dried under high vacuum until constant weight.

#### Synthesis of poly(acrylamide-co-5,6-benzo-2-methylene-1,3-dioxepane) (P(AAm-co-BMDO)) macro-CTA

A typical synthesis of P(AAm-*co*-BMDO) macro-CTA (*f*_BMDO,0_ = 0.5) was conducted as follows: in a 40 mL vial, fitted with a rubber septum and a magnetic stirring bar, a mixture of AAm (100 eq., 4.0 mmol, 0.28 g) and BMDO (100 eq., 4.0 mmol, 0.65 g) (total mole = 8 mmol), CDSPA (1 eq., 0.04 mmol, 16.1 mg) and AIBN (0.6 eq., 0.024 mmol, 3.9 mg) was dissolved in anhydrous DMSO (10 mL). The solution was bubbled with dry argon to remove dissolved oxygen for 15 min at room temperature and then immersed in a preheated oil bath at 70 °C for 8 h. The solution was then rapidly cooled under air. The copolymer was then precipitated thrice in cold THF using similar procedure as describe above for the synthesis of **P0**–**P4**. The P(AAm-*co*-BMDO) macro-CTA obtained (*M*_n, exp_ = 4800 g mol^−1^, *Đ* = 1.5, *F*_BMDO_ = 0.069) was then dried under high vacuum until constant weight.

#### Synthesis of poly(acrylamide-co-5,6-benzo-2-methylene-1,3-dioxepane)-b-poly[oligo(ethylene glycol) methyl ether methacrylate] (P(AAm-co-BMDO)-b-POEGMA, **P21**)

P(AAm-*co*-BMDO) (*M*_n, exp_ = 4800 g mol^−1^, *Đ* = 1.5, *F*_BMDO_ = 0.069) was used as a macro-RAFT agent to polymerize OEGMA and yield P(AAm-*co*-BMDO)-*b*-POEGMA diblock copolymer (**P21**, Supplementary Table 4). In a 40 mL vial, fitted with a rubber septum and a magnetic stirring bar, a mixture of OEGMA (40 eq., 250 µmol, 75 mg), P(AAm-*co*-BMDO) macro-CTA (1 eq., 6.25 µmol, 26 mg) and AIBN (0.6 eq., 3.7 µmol, 0.6 mg) was dissolved in anhydrous DMSO (4.5 mL). The solution was bubbled with dry argon to remove dissolved oxygen for 15 min at room temperature and then immersed in a preheated oil bath at 70 °C for 5 h. The solution was then rapidly cooled under air. The copolymer was then precipitated thrice in cold THF using similar procedure as describe above for the synthesis of **P0**–**P4**. The P(AAm-*co*-BMDO)-*b*-POEGMA obtained was then dried under high vacuum until constant weight.

### Degradation procedures

#### Accelerated degradation

In a 20 mL vial equipped with a magnetic stirrer, 50 mg of the desired copolymer was dissolved/dispersed in 2.5 mL of deionized water at 30 °C. After sonication and complete solubilization/dispersion, 2.5 mL of potassium hydroxide solution (5 wt.%) in deionized water was added. The mixture was then stirred for 1 h at room temperature at which time the solution turned completely transparent. The solution was then quenched by adding an aqueous solution of HCl (1 m). The resulting solution was then freeze-dried overnight to yield a white powder. The degraded product was then analyzed by SEC chromatography.

#### Hydrolytic degradation

In a 7 mL vial, 20 mg of the desired copolymer was dissolved/dispersed in 2 mL of PBS (pH 7.4) or deionized water and the solution was mechanically stirred in an orbital shaker oven (IKA KS4000i control) set at 150 rpm and thermostated at 37 °C. At specific time intervals (i.e., 1, 3, and 7 days), samples of 0.5 mL were withdrawn and freeze-dried. The degradation products were then analyzed by SEC.

#### Enzymatic degradation

In a 7 mL vial, 20 mg of the desired copolymer was dissolved/dispersed in 2 mL of PBS (pH 7.4) containing immobilized lipase from *Candida antartica* (100 U mL^−1^) and the solution was mechanically stirred in an orbital shaker oven (IKA KS4000i control) set at 150 rpm and thermostated at 37 °C. At specific time intervals (i.e., 1, 3, and 7 days), samples of 0.5 mL were withdrawn, filtered to remove the enzyme, and freeze-dried. The degradation products were then analyzed by SEC.

### Nanoparticles preparation

POEGMA-*b*-P(AAm-*co*-BMDO) nanoparticles (1.67 mg mL^−1^) were prepared by nanoprecipitation as follows. POEGMA-*b*-P(AAm-*co*-BMDO) diblock copolymers were first dissolved in deionized water prefiltered over non-sterile hydrophilic 0.22 µm polyethersulfone (PES) filters (10 mg mL^−1^) at 25 °C (*T* > UCST). Then, 1.0 mL of the copolymer solution was injected dropwise into a 20 mL glass vial prefilled with 5 mL of deionized water prefiltered over non-sterile hydrophilic 0.22 µm PES filters containing 0.1 wt.% Pluronic F68 under constant stirring (500 rpm) at 5 °C (*T* < UCST).

### In vitro cytotoxicity

#### Cell culture

HUVEC, embryonic murine fibroblast (NIH/3T3), and murine macrophage-monocyte cells (J774.A1) were purchased from American Type Culture Collection (ATCC) and maintained as recommended. Fetal bovine serum (FBS) was purchased from Gibco, Penicillin-Streptomycin stabilized solution, Dulbecco’s Modified Eagle Medium (DMEM) and RPMI-1640 medium were purchased from Sigma-Aldrich and used as received. J774.A1 cells were grown in Roswell Park Memorial Institute medium (RPMI) 1640 supplemented with 10% FBS, penicillin (50 U mL^−1^), and streptomycin (50 U mL^−1^). NIH/3T3 and HUVEC cells were grown in DMEM high glucose supplemented with 10% FBS, penicillin (50 U mL^−1^) and streptomycin (50 U mL^−1^). Cells were maintained in a humid atmosphere at 37 °C with 5% CO_2_.

#### Cell viability assay

MTT was purchased from Sigma-Aldrich and used as received. The dried copolymers (**P9**–**P14** and **P17**) were weighted and solubilized with the appropriate medium to achieve the desired concentrations in 10 mL sterilized flacon tubes. Samples **P9**–**P14** were warmed up directly in a water bath at 37 °C for at least 10 min whereas the water bath temperature was adjusted to 50 °C for sample **P17**. The solubilized samples were then filtered by sterilized filters (0.22 µm, Minisart®) before use. In 96-well microtiter plates (TPP, Switzerland), cells were seeded (HUVEC: 2 × 10^4^ cells mL^−1^, NIH/3T3: 4 × 10^4^ cells mL^−1^, J774.A1: 2 × 10^4^ cells mL^−1^) in 100 μL of growth medium and preincubated for 24 h in incubator (37 °C and 5% CO_2_). After appropriate dilutions, 100 μL of copolymer solution in cell culture medium (0.01 and 0.1 mg mL^−1^) was added to the cells and incubated for 72 h. An MTT solution (5 mg mL^−1^) was prepared with PBS and filtered with sterile filters (0.2 µm). At the end of the incubation period, 20 µL of MTT solution was added to each well. After incubation (1 h for HUVEC and J774.A1 cells, 1.5 h for NIH/3T3 cells), the medium was removed and 200 μL of DMSO was then added to each well to dissolve the formazan crystals. The absorbance was then measured by a microplate reader (LAB Systems Original Multiscan MS) at 570 nm. Cell viability was calculated as the absorbance ratio between treated and untreated control cells. All experiments were performed in triplicate to determine means and SD.

#### Cell morphology observation

Cells were seeded in 100 μL of growth medium and preincubated for 24 h in an incubator (37 °C and 5% CO_2_) in 96-well microtiter plates. The same procedure was applied for copolymers as in the Cell Viability Assay section. 100 μL of copolymer solution in cell culture medium (0.01 and 0.1 mg mL^−1^) with adjusted concentration was added over the cells. Cells morphology was directly observed after 72 h incubation with an AxioObserver Z1 (Carl Zeiss, Germany) inverted microscope equipped with an XL incubator providing 37 °C, a charge-coupled device (CCD) CoolSnap-HQ2 camera (6.45 µm pixel size; Photometrics, Tucson, USA) and an Achroplan 4×/0.10 NA dry objective lens using a brightfield mode (TL halogen lamp). 12 bits numerical images were done with Zen 2.6 software (blue edition).

### Reporting summary

Further information on research design is available in the Nature Research Reporting Summary linked to this article.

## Supplementary information

Supplementary Information

Description of Additional Supplementary Files

Supplementary Movie 1

Reporting Summary

## Data Availability

All data generated and analyzed during this study are included in this article and its Supplementary Information and are also available from the corresponding author upon request. Source data are provided in this paper.

## Source data

Source Data
