# Supplementary material for: Vinyl copolymers with faster hydrolytic degradation than aliphatic polyesters and tunable upper critical solution temperatures
Source: Nat Commun. 2022 May 24;13:2873. doi: 10.1038/s41467-022-30220-y (PMC9130262; doi:10.1038/s41467-022-30220-y)
Supplement: Supplementary file 1 — Supplementary Information [file 41467_2022_30220_MOESM1_ESM.pdf]

Supplementary Information

Vinyl Copolymers with Faster Hydrolytic Degradation than Aliphatic Polyesters and Tunable Upper Critical Solution Temperatures

Amaury Bossion,[1] Chen Zhu,[1] Léa Guerassimoff,[1] Julie Mougin,[1] Julien Nicolas,[1]*

[1] *Université Paris-Saclay, CNRS, Institut Galien Paris-Saclay, 92296 Châtenay-Malabry, France*

*To whom correspondence should be addressed.

Email: julien.nicolas@u-psud.fr

Tel.: +33 1 46 83 58 53

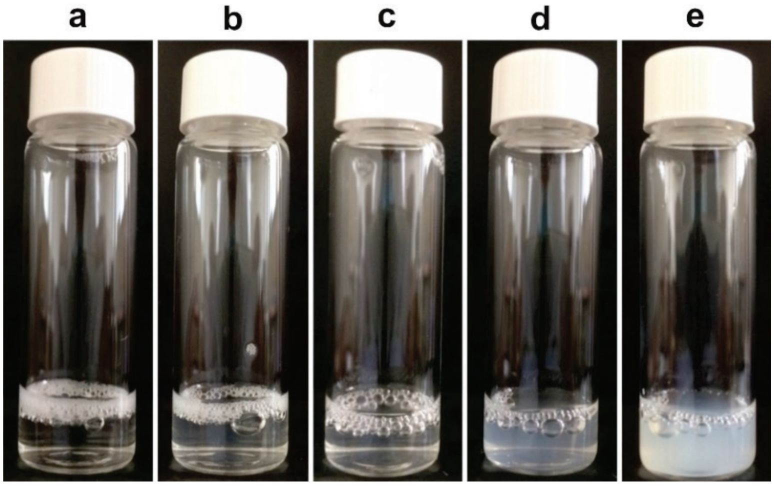

Supplementary Figure 1. Pictures of the P(AAm-*co*-MPDL) copolymer solutions in deionized water (10 mg.mL^{-1}) at room temperature as a function of F_{MPDL}. **a P0** (F_{MPDL} = 0, no T_{cp}); **b P1** (F_{MPDL} = 0.038, no T_{cp}); **c P2** (F_{MPDL} = 0.043, T_{cp} = 15°C); **d P3** (F_{MPDL} = 0.067, T_{cp} = 18°C); **e P4** (F_{MPDL} = 0.108, T_{cp} = 38°C).

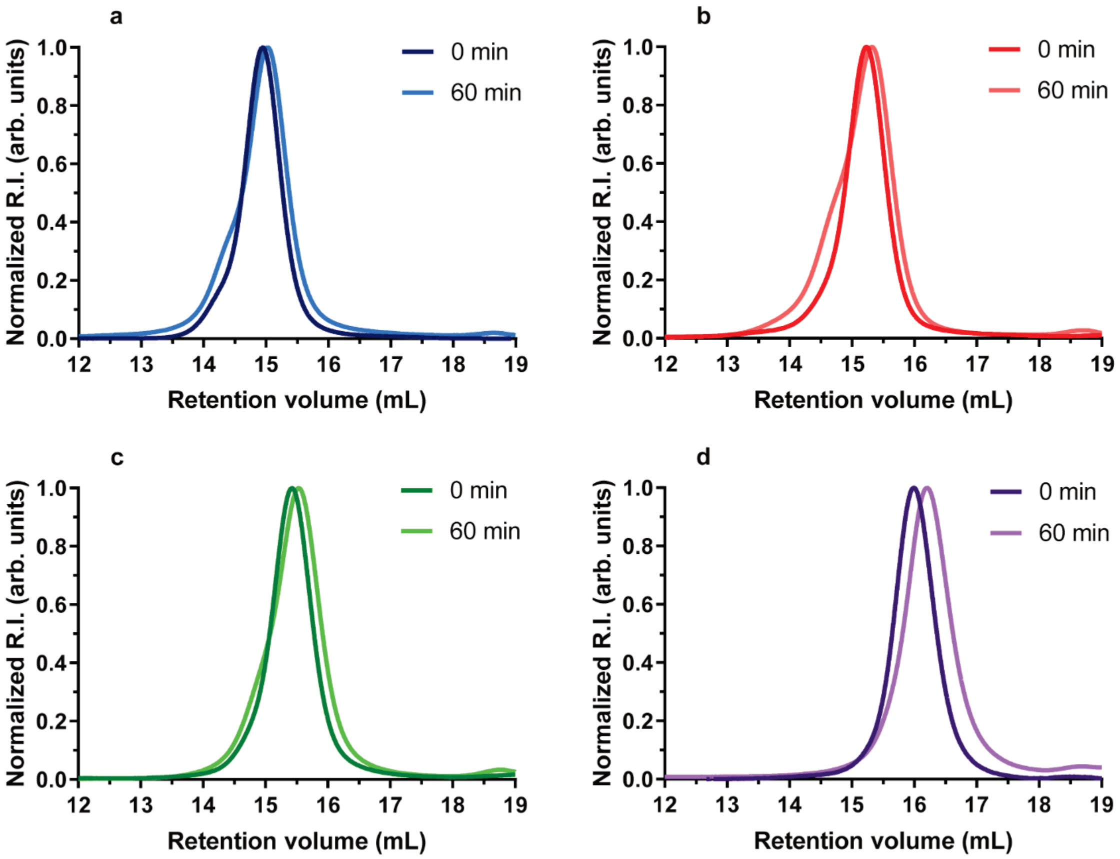

Supplementary Figure 2. Evolution of the SEC chromatograms at different time during the hydrolytic degradation under accelerated conditions (5 wt. % KOH) of P(AAm-*co*-MPDL) copolymers (Table 1, **P0–P3**) as a function of AAm to MPDL molar ratio. **a P0** (F_{MPDL} = 0, no T_{cp}); **b P1** (F_{MPDL} = 0.038, no T_{cp}); **c P2** (F_{MPDL} = 0.043, T_{cp} = 15°C); **d P3** (F_{MPDL} = 0.067, T_{cp} = 18°C).

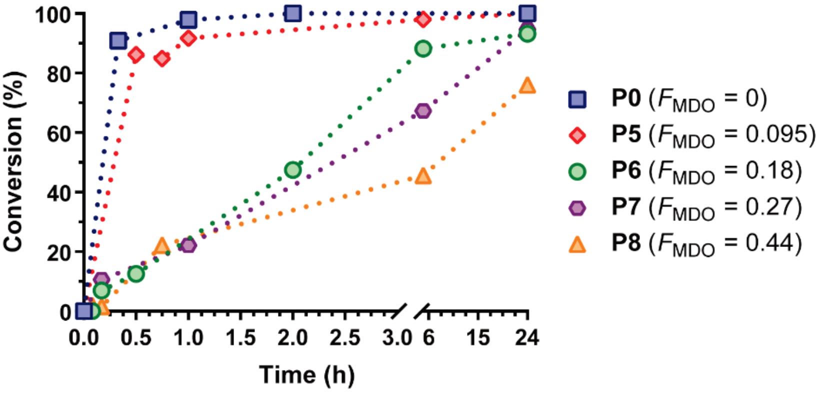

Supplementary Figure 3. Conversion *vs.* time kinetic plot from the RAFT polymerization of AAm with MDO (Table 1, **P0**, **P5 –P8**) in anhydrous DMSO initiated by AIBN at 70°C as a function of F_{MDO}. Conversion = AAm conversion as determined by ^{1}H NMR.

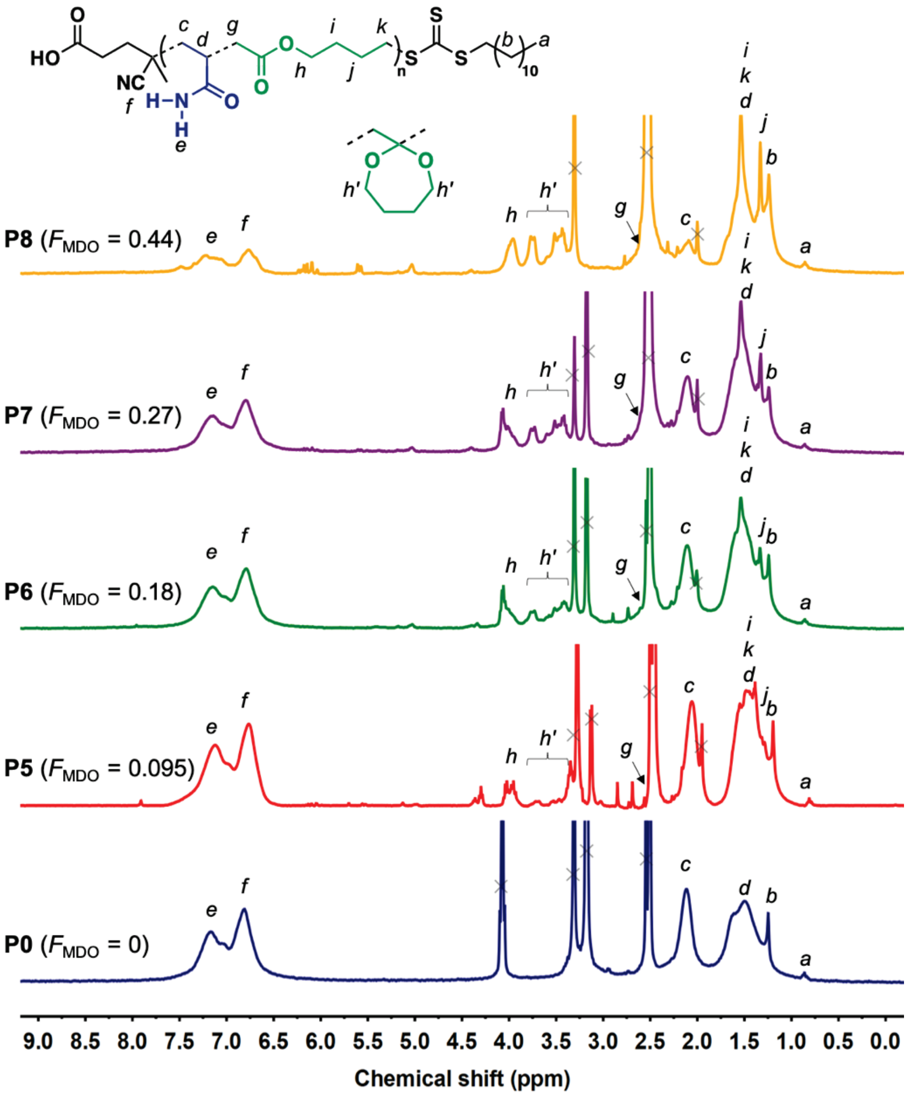

Supplementary Figure 4. ^{1}H NMR spectrum (300 MHz, DMSO-d6) in the 0–9 ppm region of P(AAm-*co*-MDO) copolymers (Table 1, **P0** and **P5–P8**). Note: Precipitation was performed in diethyl ether for **P8** so traces of unreacted AAm monomer can be seen on the spectrum.

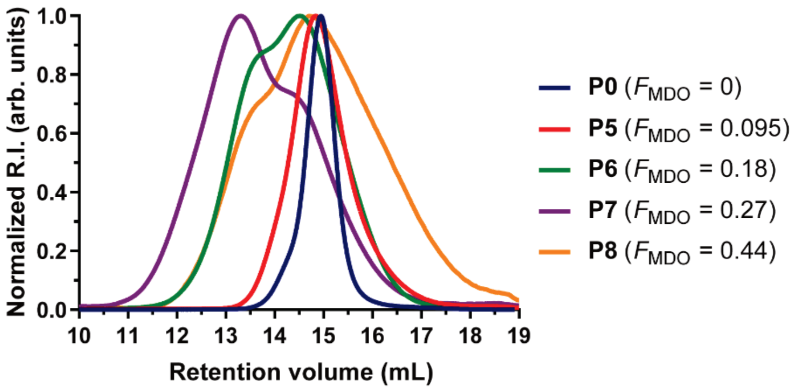

Supplementary Figure 5. Evolution of the SEC chromatograms of P(AAm-*co*-MDO) copolymers (Table 1, **P0** and **P5–P8**) as a function of F_{MDO}.

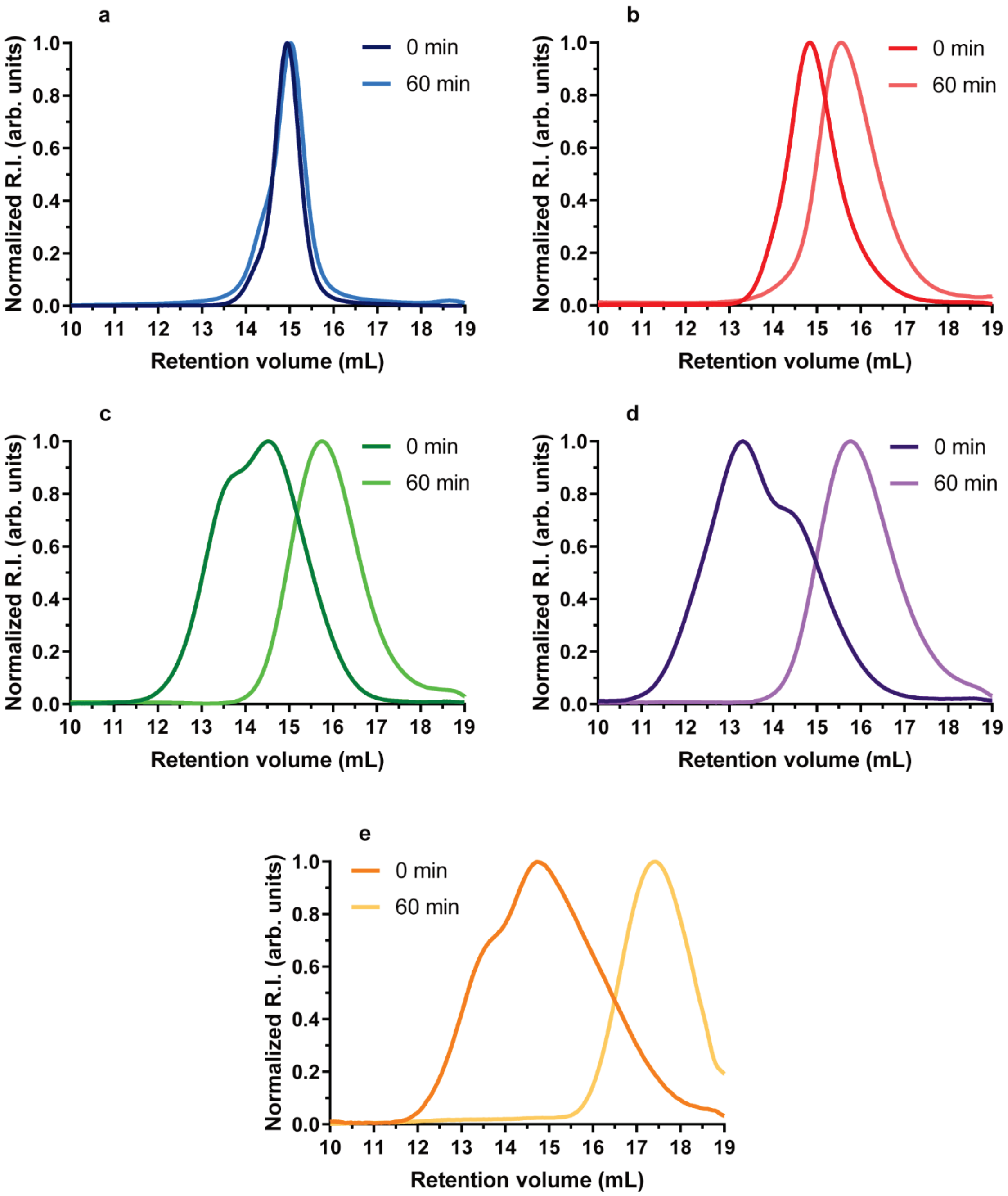

Supplementary Figure 6. Evolution of the SEC chromatograms at different time during the hydrolytic degradation under accelerated conditions (5 wt. % KOH) of P(AAm-*co*-MDO) copolymers (Table 1, **P0** and **P5–P8**) as a function of F_{MDO}. **a P0** (F_{MDO} = 0, no T_{cp}); **b P5** (F_{MDO} = 0.095, no T_{cp}); **c P6** (F_{MDO} = 0.18, no T_{cp}); **d P7** (F_{MDO} = 0.27, no T_{cp}); **e P8** (F_{MDO} = 0.44, no T_{cp}).

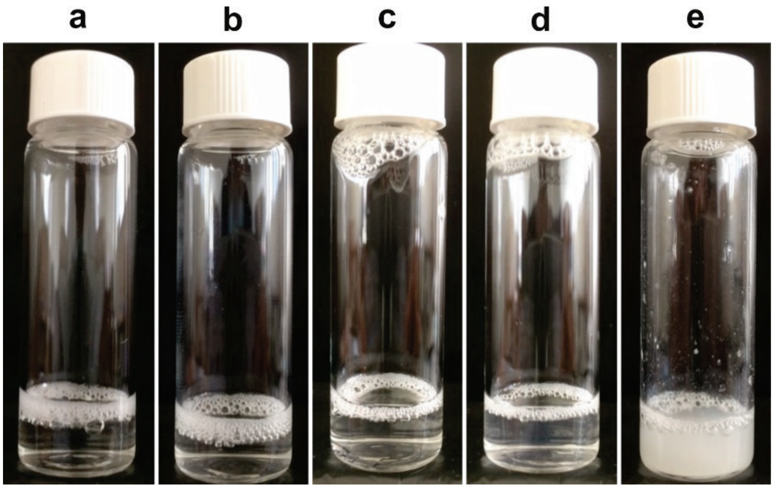

Supplementary Figure 7. Pictures of the P(AAm-*co*-MDO) copolymer (Table 1, **P0** and **P5–P8**) solutions in deionized water (10 mg.mL^{-1}) at room temperature as a function of F_{MDO}. **a P0** (F_{MDO} = 0, no T_{cp}); **b P5** (F_{MDO} = 0.095, no T_{cp}); **c P6** (F_{MDO} = 0.18, no T_{cp}); **d P7** (F_{MDO} = 0.27, no T_{cp}); **e P8** (F_{MDO} = 0.44, no T_{cp}).

Supplementary Table 1. Experimental Conditions and Macromolecular Characteristics of UCST-type P(AAm-*co*-BMDO) ($f_{BMDO,0}$ = 0.4) Copolymers Synthesized by RAFT-Mediated Copolymerization of AAm and BMDO in Anhydrous DMSO at 70°C for 16 h at Different Concentration.

Entry	BMDO concentration	$f_{BMDO,0}$	F_{BMDO}[a]	Open BMDO (%)[a]	AAm conv. (%)[b]	T_{cp} from UV (°C)[c]		$M_{n,\ NMR}$[d] (g.mol^{-1})	$M_{n,\ exp.}$[e] (g.mol^{-1})	$Đ_{exp.}$[e]
						cooling	heating			
**P13-1**	8 M 43.9 wt.%	0.4	0.098	75	> 98	13	-[f]	7,400	11,300	1.5
**P13-2**	4 M 28.1 wt.%	0.4	0.087	80	> 98	17	-[f]	7,300	11,300	1.3
**P13-3**	2 M 16.4 wt.%	0.4	0.12	81	83	38	33	6,900	9,200	1.3

[a] Determined by ^{1}H NMR after precipitation by integrating the 2H (–NH_2) of AAm, the 4H (aromatic protons) of open and closed BMDO (6.5–7.5 ppm), the 2H of open BMDO (4.9–5.2 ppm) and the 4H of closed BMDO (4.5–4.8 ppm). [b] Determined by ^{1}H NMR by integrating the 2H of AAm (6.02–6.24 ppm) at t = 0 and 16 h. [c] Determined from the maximum of the first derivative of the cooling/heating curves obtained by UV-vis temperature ramp (1°C.min^{-1}) at 10 mg.mL^{-1} in deionized water. [d] Determined by ^{1}H NMR after precipitation by integrating the 3H from the CH_3 moiety of the RAFT agent C12 alkyl chain (0.86 ppm), the 1H of AAm (2.1 ppm), the 2H of open BMDO (4.9–5.2 ppm) and the 4H of closed BMDO (4.5–4.8 ppm). Note that this method is only accurate for high living chain fractions. [e] Determined by SEC in DMSO with 100 mM LiBr. [f] Aggregation/precipitation occurred at low temperature. The cloud point upon cooling could be recovered after sonication at room temperature for 2 min.

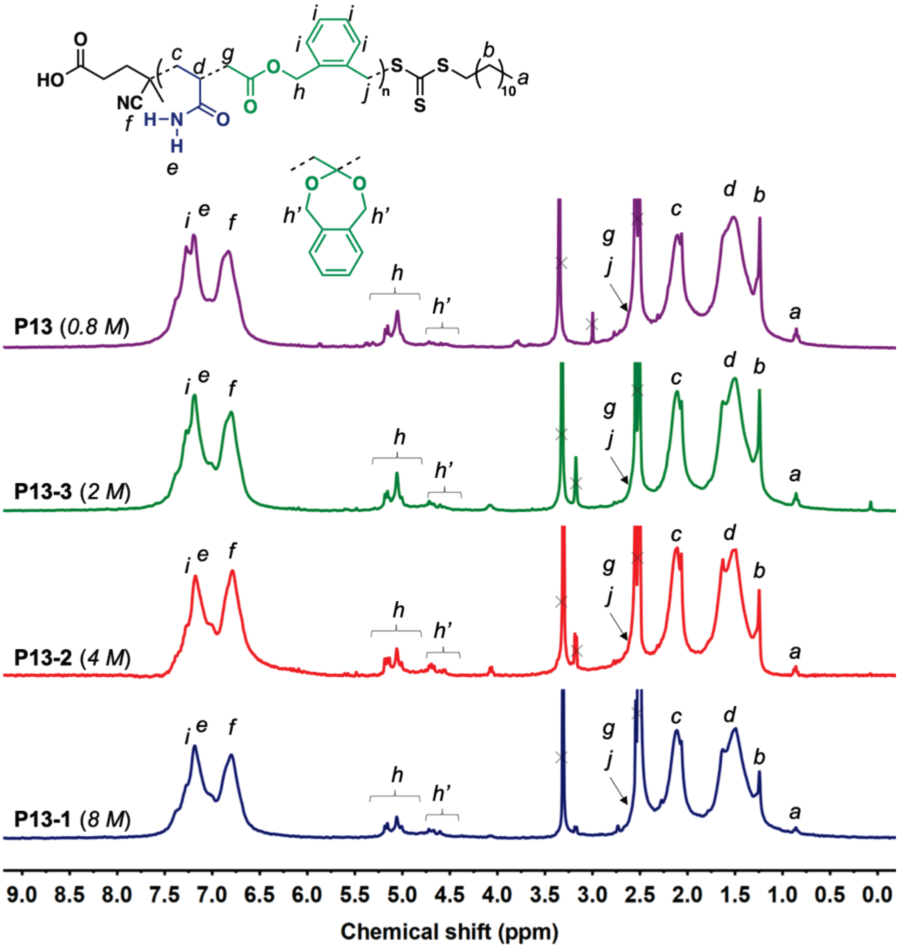

Supplementary Figure 8. ^{1}H NMR spectrum (300 MHz, DMSO-d$_6$) in the 0–9 ppm region of P(AAm-*co*-BMDO) copolymers **P13** ($f_{BMDO,0}$ = 0.4) synthesized at different concentration.

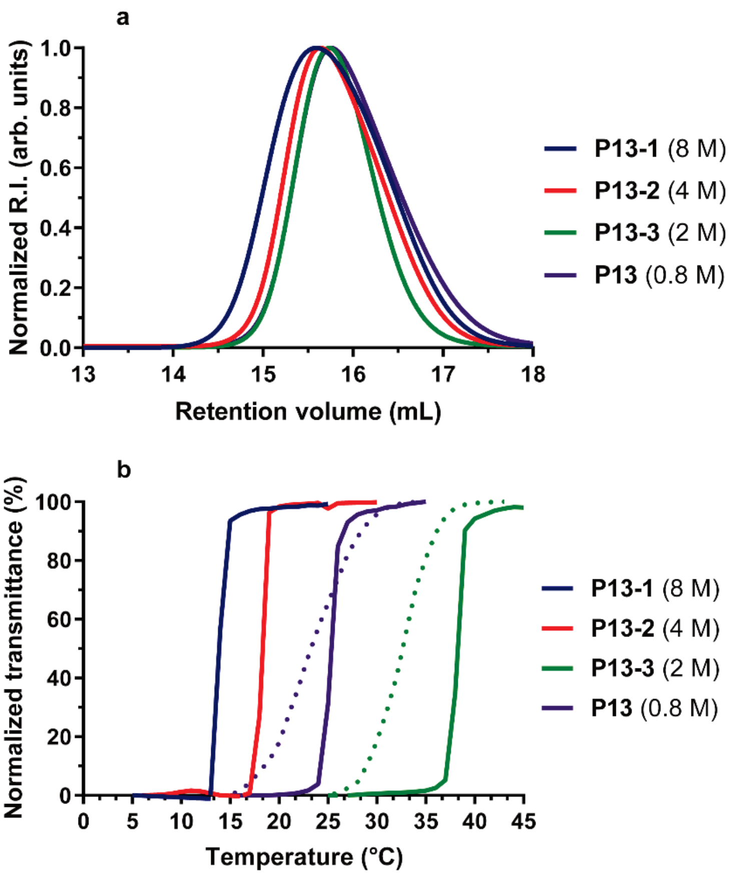

Supplementary Figure 9. a Evolution of the SEC chromatograms of P(AAm-*co*-BMDO) copolymers with $f_{BMDO,0}$ = 0.4 synthesized at different concentration. **b** Variation of the solution transmittance vs. temperature of P(AAm-*co*-BMDO) copolymer solution in water (10 mg.mL^{-1}) with $f_{BMDO,0}$ = 0.4 synthesized at different concentration, subjected to consecutive cooling and heating cycles at 1°C.min^{-1}; solid and dotted lines for cooling and heating scan, respectively.

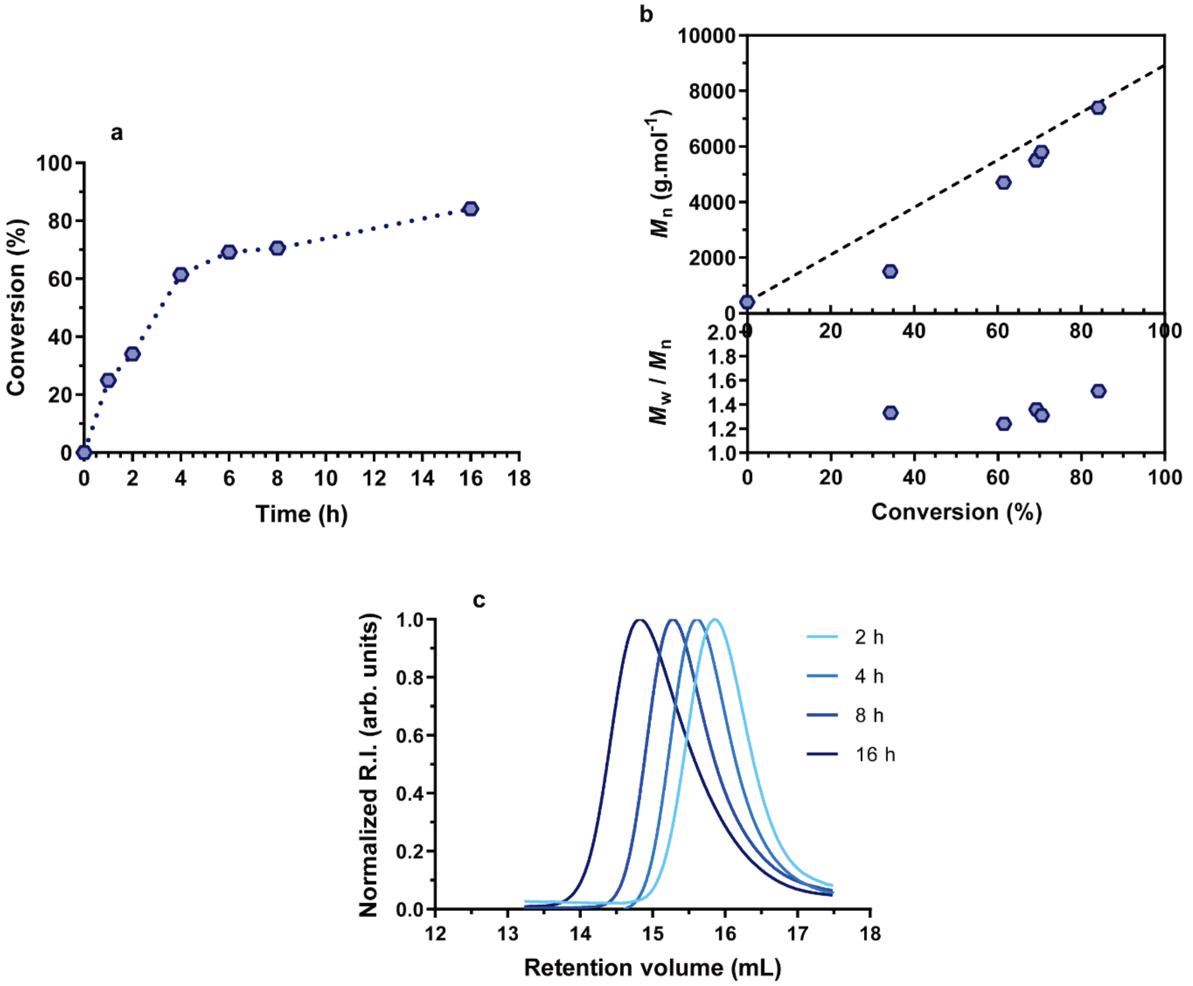

Supplementary Figure 10. Evolution of: **a** the conversion with time; **b** the number-average molar mass (M_n) and dispersity with AAm conversion and **c** the SEC chromatograms during the copolymerization at 0.8 M of AAm and BMDO (**P13**, $f_{BMDO,0}$ = 0.4) in anhydrous DMSO at 70°C for 16 h.

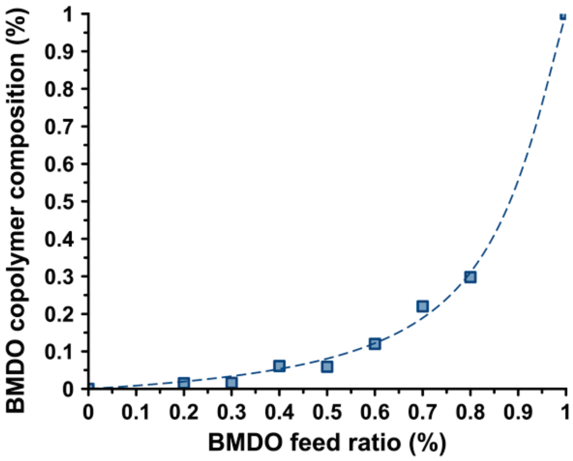

Supplementary Figure 11. Copolymer composition curve for the copolymerization of AAm and BMDO in anhydrous DMSO at 70 °C with [M]/[CTA]/[AIBN] = 200:1:0.6. Polymerizations were terminated at conversion ≤ 20%. The blue dash line corresponds to the nonlinear least-squares fitting curve where $\boldsymbol{r}_{\mathrm{AAm}}$ = 13.02 and $\boldsymbol{r}_{\mathrm{BMDO}}$ = 0.23.

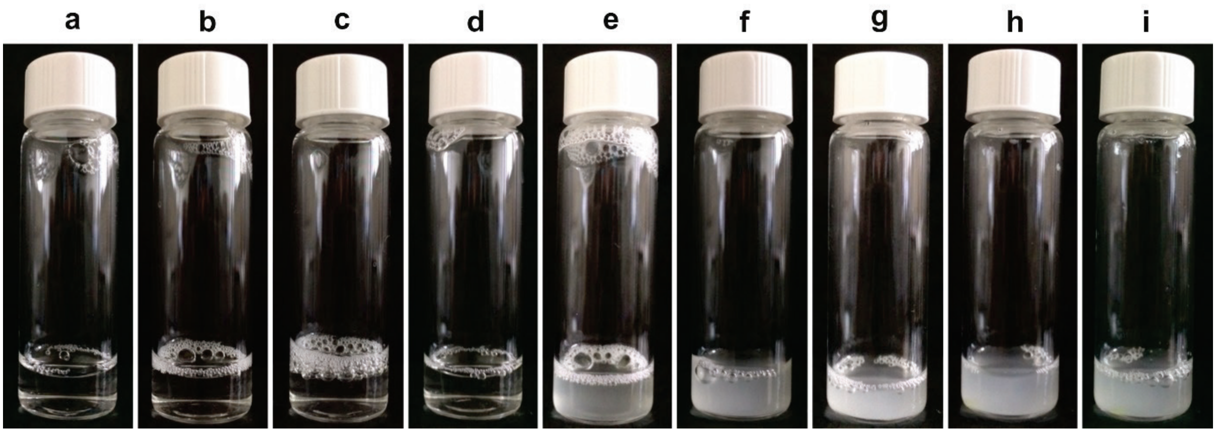

Supplementary Figure 12. Pictures of the P(AAm-*co*-BMDO) copolymer (Table 2, **P9–P17**) solutions in deionized water (10 mg.mL^{-1}) at room temperature as a function F_{BMDO}. **a P9** (F_{BMDO} = 0, no T_{cp}); **b P10** (F_{BMDO} = 0.017, no T_{cp}); **c P11** (F_{BMDO} = 0.027, no T_{cp}); **d P12** (F_{BMDO} = 0.068, no T_{cp}); **e P13** (F_{BMDO} = 0.093, T_{cp} = 25°C); **f P14** (F_{BMDO} = 0.102, T_{cp} = 33°C); **g P15** (F_{BMDO} = 0.113, T_{cp} = 46°C); **h P16** (F_{BMDO} = 0.126 T_{cp} = 49°C); **i P17** (F_{BMDO} = 0.128, T_{cp} = 52°C).

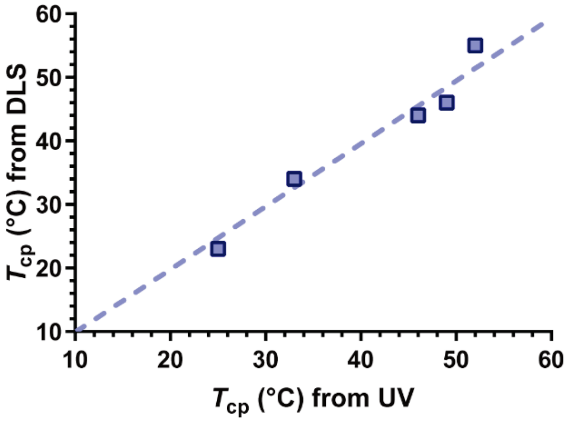

Supplementary Figure 13. Comparison of T_{cp} measured by DLS and UV for copolymers **P13–P17**. The blue line is an identity line (y = x).

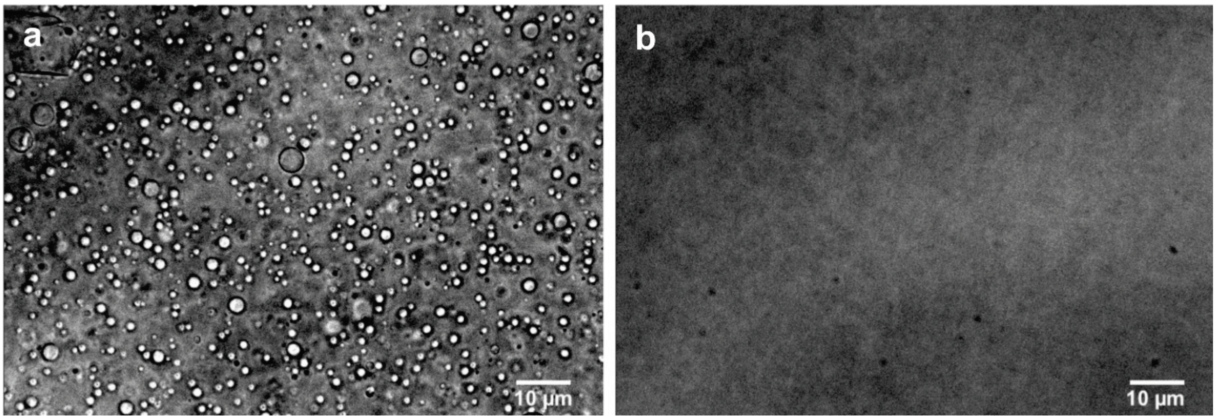

Supplementary Figure 14. Representative images obtained by optical microscopy of P(AAm-*co*-BMDO) **P14** (Table 2) solution in water (10 mg.mL^{-1}) at: **a** T < UCST and **b** T > UCST. This experiment was repeated four times with similar results.

Supplementary Table 2. Experimental Conditions and Macromolecular Characteristics of P(AAm-*co*-BMDO) Copolymers Synthesized by RAFT-Mediated Copolymerization of AAm and BMDO ($f_{BMDO,0}$ = 0.4) in Anhydrous DMSO at 70°C for 16 h as Function of the Targeted Average Degree of Polymerization.

Entry	$f_{BMDO,0}$	F_{BMDO} [a]	Open BMDO (%) [a]	$DP_{n,\ th}$	AAm conv. (%) [b]	T_{cp} from UV (°C) [c]		T_{cp} from DLS (°C) [d]		$M_{n,\ NMR}$ [e] (g.mol^{-1})	$M_{n,\ exp.}$ [f] (g.mol^{-1})	$Đ_{exp.}$ [f]	$M_{n,\ deg.\ theo.}$ [g] (g.mol^{-1}) (% M_n loss)	$M_{n,\ deg.}$ [h] (g.mol^{-1}) (% M_n loss)
						cooling	heating	cooling	heating					
**P13**	0.4	0.093	88	200	71	25	22	23	23	6,900	7,600	1.4	960 (- 87 %)	2,100 (- 72 %)
**P18**	0.4	0.083	89	400	58	18	19	17	17	8,700	8,400	1.6	1,050 (- 88 %)	2,300 (- 73 %)
**P19**	0.4	0.074	90	600	48	12	12	12	13	10,000	10,200	1.8	1,200 (- 88 %)	2,400 (- 76 %)

[a] Determined by ^{1}H NMR after precipitation by integrating the 2H (–NH_2) of AAm, the 4H (aromatic protons) of open and closed BMDO (6.5–7.5 ppm), the 2H of open BMDO (4.9–5.2 ppm) and the 4H of closed BMDO (4.5–4.8 ppm). [b] Determined by ^{1}H NMR by integrating the 2H of AAm (6.02–6.24 ppm) at t = 0 and 16 h. [c] Determined from the maximum of the first derivative of the cooling/heating curves obtained by UV-vis temperature ramp (1°C.min^{-1}) at 10 mg.mL^{-1} in deionized water. [d] Determined from the maximum of the first derivative of the cooling/heating curves obtained by DLS temperature ramp at 10 mg.mL^{-1} in deionized water. [e] Determined by ^{1}H NMR after precipitation by integrating the 3H from the CH_3 moiety of the RAFT agent C12 alkyl chain (0.86 ppm), the 1H of AAm (2.1 ppm), the 2H of open BMDO (4.9–5.2 ppm) and the 4H of closed BMDO (4.5–4.8 ppm). Note that this method is only accurate for high living chain fractions. [f] Determined by SEC in DMSO with 100 mM LiBr. [g] Determined according to: $M_{n,\ deg.\ theo}$ = ([1 / (open BMDO x F_{BMDO})] – 1) × MW_{AAm} + MW_{BMDO}, with MW being the molecular weight of the considered monomer. [h] Determined by SEC in DMSO with 100 mM LiBr after polymer hydrolytic degradation using KOH 5 wt.% solution.

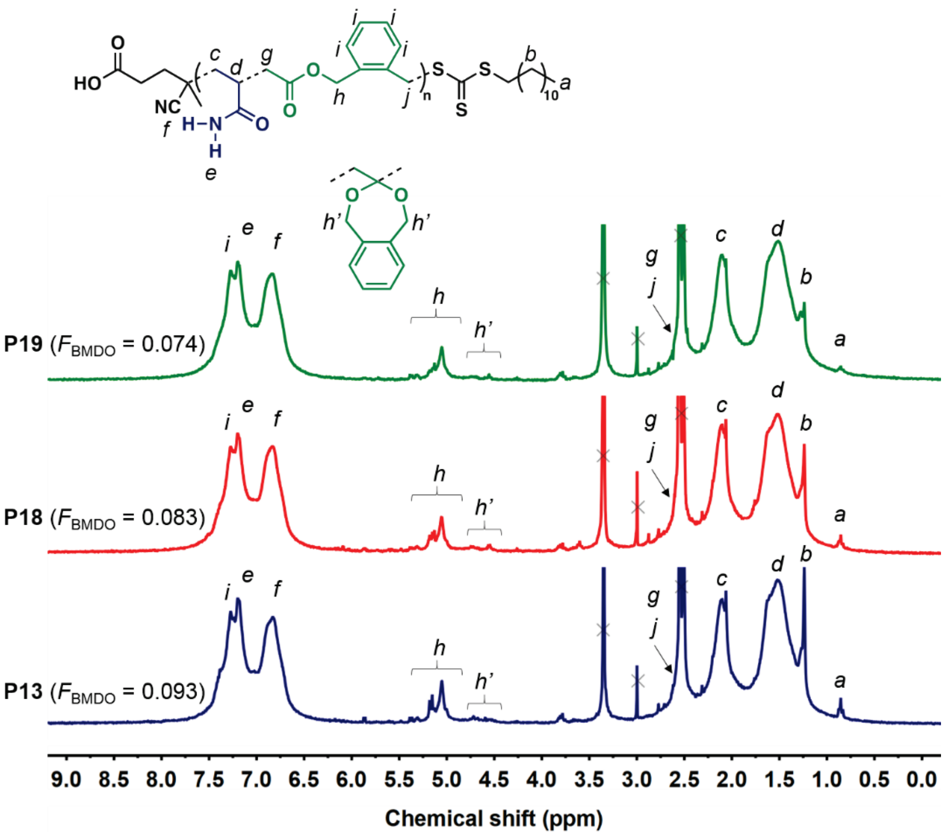

Supplementary Figure 15. ^{1}H NMR spectrum (300 MHz, DMSO-d$_6$) in the 0–9 ppm region of P(AAm-*co*-BMDO) copolymers (Supplementary Table 2, **P13** and **P18–P19**) with $f_{BMDO,0}$ = 0.4 and different degrees of polymerization ($DP_{n,\,th}$): **P13** (F_{BMDO} = 0.093, $DP_{n,\,th}$ = 200); **P18** (F_{BMDO} = 0.083, $DP_{n,\,th}$ = 400) and **P19** (F_{BMDO} = 0.074, $DP_{n,\,th}$ = 600).

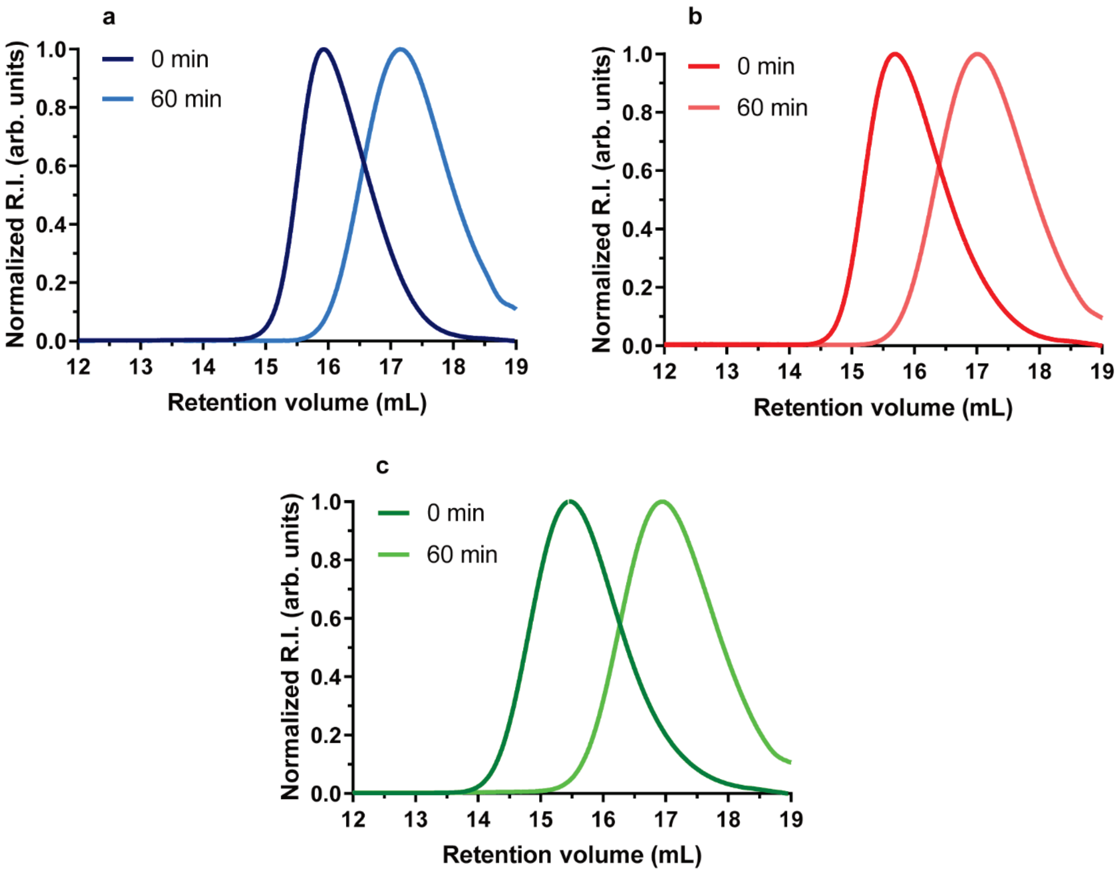

Supplementary Figure 16. Evolution of the SEC chromatograms at different time during the hydrolytic degradation under accelerated conditions (5 wt. % KOH) of P(AAm-*co*-BMDO) copolymers (Supplementary Table 2, **P13–P18**) with $f_{BMDO,0}$ = 0.4 and different degrees of polymerization ($DP_{n,\,th}$). **a P13** (F_{BMDO} = 0.093, $DP_{n,\,th}$ = 200); **b P18** (F_{BMDO} = 0.083, $DP_{n,\,th}$ = 400); **c P19** (F_{BMDO} = 0.074, $DP_{n,\,th}$ = 600).

Supplementary Table 3. Macromolecular Characteristics of P(AAm-*co*-BMDO) and P(AAm-*co*-MDO) Copolymers After Degradation Under Different Conditions: Accelerated Conditions (5 wt.% KOH), Physiological Conditions (PBS, pH 7.4, 37°C) and In Presence of Enzymes (*Candida antartica*, pH 7.4, 37°C).

	Entry	$f_{CKA,0}$	F_{CKA}[a]	Open CKA (%)[a]	AAm conv. (%)[b]	$M_{n, NMR}$[c] (g.mol^{-1})	$M_{n, exp.}$[d] (g.mol^{-1})	$Đ_{exp.}$[d]	$M_{n, deg. theo.}$[e] (g.mol^{-1}) (% M_n loss)	$M_{n, deg. KOH}$[f] (g.mol^{-1}) (% M_n loss)	$M_{n, deg. hydrol.}$[g] (g.mol^{-1}) (% M_n loss)	$M_{n, deg. enzyma.}$[h] (g.mol^{-1}) (% M_n loss)
BMDO	**P9**	0	0	0	96	14,500	18,600	1.5	-	20,800	19,700	20,300
BMDO	**P10**	20	0.017	98	85	6,300	13,500	1.5	4,300 (- 68 %)	7,400 (- 45 %)	7,500 (- 44 %)	-
BMDO	**P11**	30	0.027	96	84	8,800	12,200	1.4	2,800 (- 77 %)	5,000 (- 59 %)	5,600 (- 54 %)	-
BMDO	**P12**	35	0.068	87	75	5,100	7,700	1.4	1,300 (- 83 %)	3,200 (- 58 %)	3,500 (- 55 %)	-
BMDO	**P13**	40	0.093	88	71	6,900	7,600	1.4	960 (- 87 %)	2,100 (- 72 %)	2,600 (- 66 %)	3,300 (- 57 %)
BMDO	**P17**	55	0.128	87	67	6,000	6,100	1.4	730 (- 88 %)	1,600 (- 74 %)	2,100 (- 66 %)	2,700 (- 56 %)
MDO	**P6**	40	0.18	43	93	5,600	37,900	2.3	960 (- 97 %)	7,500 (- 80 %)	13,300 (- 65 %)	23,900 (- 37 %)
MDO	**P8**	80	0.44	43	76	7,100	14,000	4.4	420 (- 97 %)	1,700 (- 87 %)	3,100 (- 75 %)	3,300 (- 74 %)

[a] Determined by ^{1}H NMR after precipitation by: (i) integrating the 2H (–NH_2) of AAm, the 4H (aromatic protons) of open and closed BMDO (6.5–7.5 ppm), the 2H of open BMDO (4.9–5.2 ppm) and the 4H of closed BMDO (4.5–4.8 ppm) for BMDO and (ii) by integrating the 2H (–NH_2) of AAm (6.5–7.5 ppm), the 2H of open MDO (3.9–4.1 ppm) and the 4H of closed MDO (3.4–3.8 ppm) for MDO. [b] Determined by ^{1}H NMR by integrating the 2H of AAm (6.02–6.24 ppm) at t = 0 and 16h. [c] Determined by ^{1}H NMR after precipitation by: (i) integrating the 3H from the CH_3 moiety of the RAFT agent C12 alkyl chain (0.86 ppm), the 1H of AAm (2.1 ppm) and: (i) the 2H of open BMDO (4.9–5.2 ppm) and the 4H of closed BMDO (4.5–4.8 ppm) or (ii) the 2H of open MDO (3.9–4.1 ppm) and the 4H of closed MDO (3.4–3.8 ppm). Note that this method is only accurate for high living chain fractions. [d] Determined by SEC in DMSO with 100 mM LiBr. [e] Determined according to: $M_{n, deg. theo} = ([1 / (\text{open CKA} \times F_{CKA})] - 1) \times MW_{AAm} + MW_{CKA}$, with MW being the molecular weight of the considered monomer. [f] Determined by SEC in DMSO with 100 mM LiBr after polymer hydrolytic degradation using KOH 5 wt.% solution. [g] Determined by SEC in DMSO with 100 mM LiBr after polymer long-term hydrolytic degradation (1 week). [h] Determined by SEC in DMSO with 100 mM LiBr after polymer after long-term enzymatic degradation (1 week).

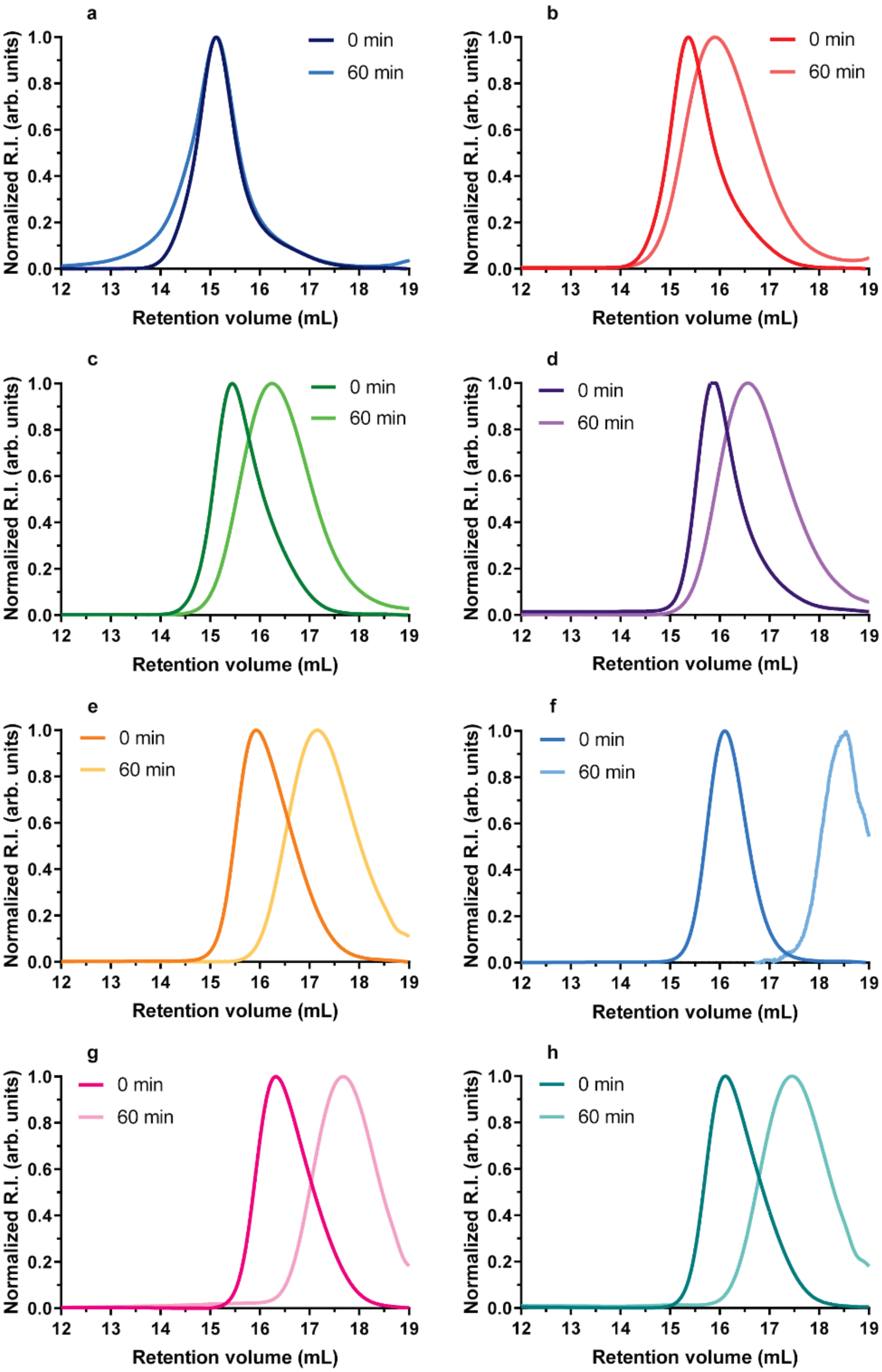
a
b
c
d
e
f
g
h
0 min
60 min
Normalized R.I. (arb. units)
Retention volume (mL)
0.0
0.2
0.4
0.6
0.8
1.0
12
13
14
15
16
17
18
19

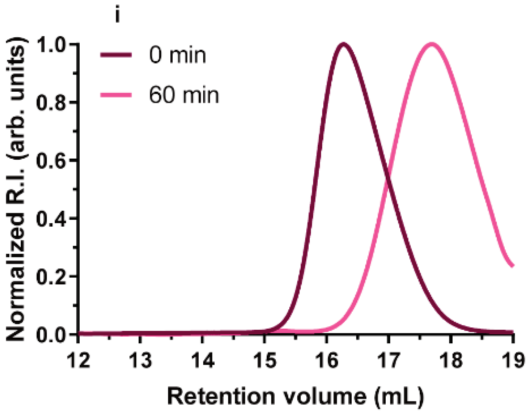

Supplementary Figure 17. Evolution of the SEC chromatograms at different time during the hydrolytic degradation under accelerated conditions (5 wt. % KOH) of P(AAm-*co*-BMDO) copolymers (Table 2, **P9–P17**) as a function F_{BMDO}. **a P9** (F_{BMDO} = 0, no T_{cp}); **b P10** (F_{BMDO} = 0.017, no T_{cp}); **c P11** (F_{BMDO} = 0.027, no T_{cp}); **d P12** (F_{BMDO} = 0.068, no T_{cp}); **e P13** (F_{BMDO} = 0.093, T_{cp} = 25°C); **f P14** (F_{BMDO} = 0.102, T_{cp} = 33°C); **g P15** (F_{BMDO} = 0.113, T_{cp} = 46°C); **h P16** (F_{BMDO} = 0.126, T_{cp} = 49°C); **i P17** (F_{BMDO} = 0.128, T_{cp} = 52°C).

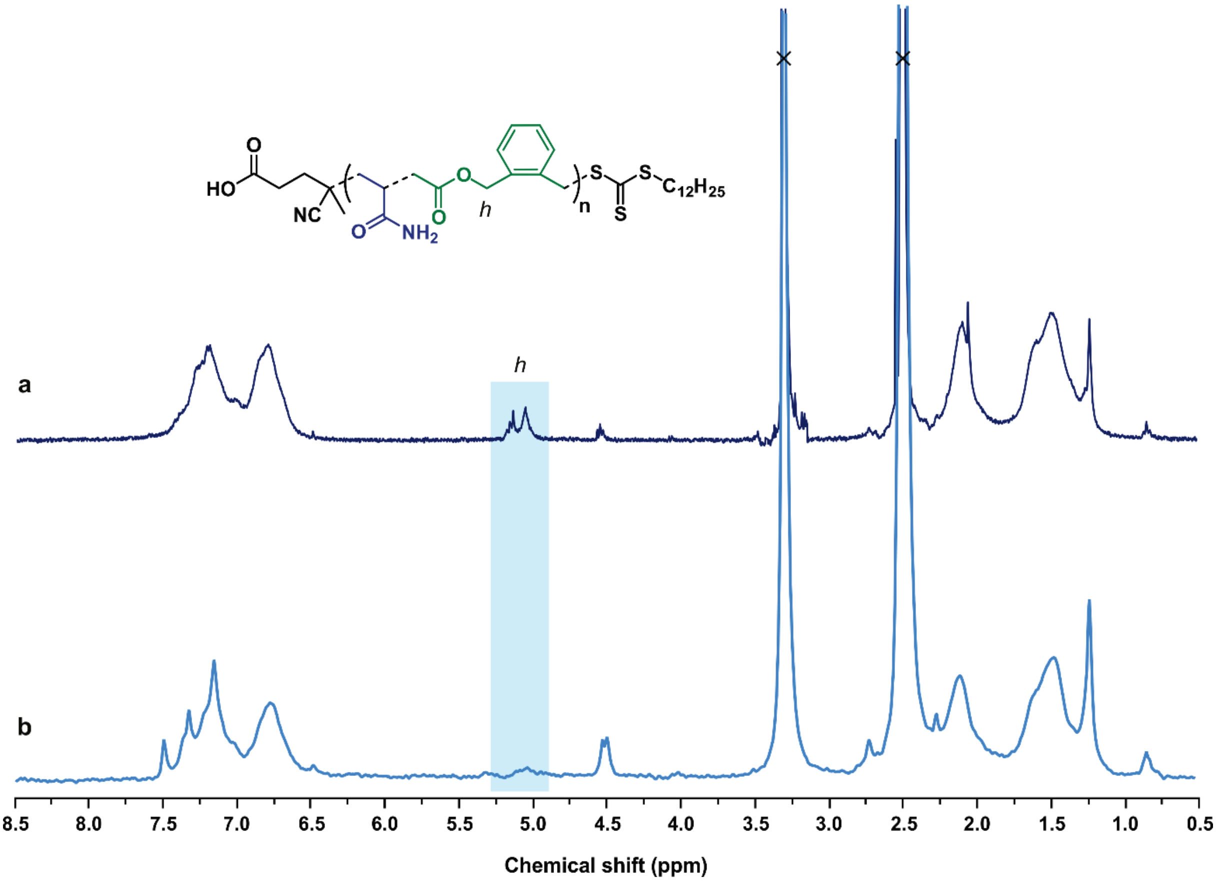

Supplementary Figure 18. ^{1}H NMR spectra (300 MHz, DMSO-d$_6$) in the 0.5–8.5 ppm region of P(AAm-*co*-BMDO) copolymer **P14**, **a** before and **b** after hydrolytic degradation under accelerated conditions for 1 h.

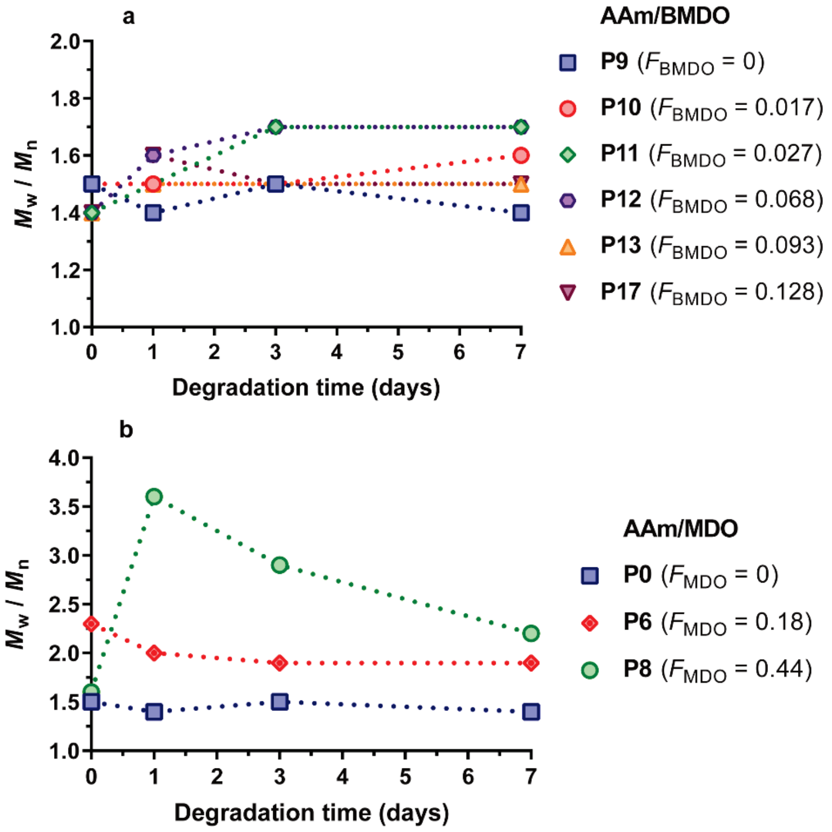

Supplementary Figure 19. Evolution with time of the dispersity of the copolymers during hydrolytic degradation under physiological conditions (PBS, pH 7.4, 37°C) of: **a** P(AAm-*co*-BMDO) **P9–P13** and **P17**, and **b** P(AAm-*co*-MDO) **P0**, **P6** and **P8**.

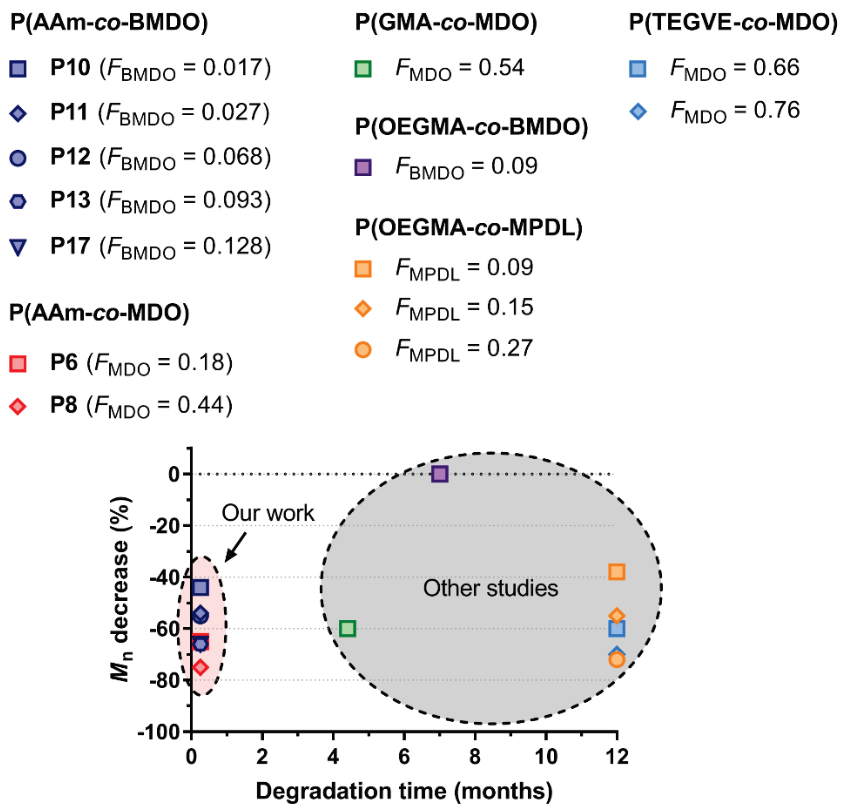

Supplementary Figure 20. Maximum decrease in number-average molar mass (M_n) after degradation of CKA-containing copolymers in physiological conditions (PBS, pH 7.4), depending on the nature of the copolymer and the molar fraction of CKA (refs: *Biomacromolecules* **2014**, *15*, 7, 2800, *Eur. Polym. J.* **2015**, *65*, 305, *Macromolecules* **2018**, *51*, 3, 724, *Biomacromolecules* **2019**, *20*, 1, 305). Note: all degradation experiments were performed at 37°C except for the purple square where the degradation experiment was performed at 4°C.

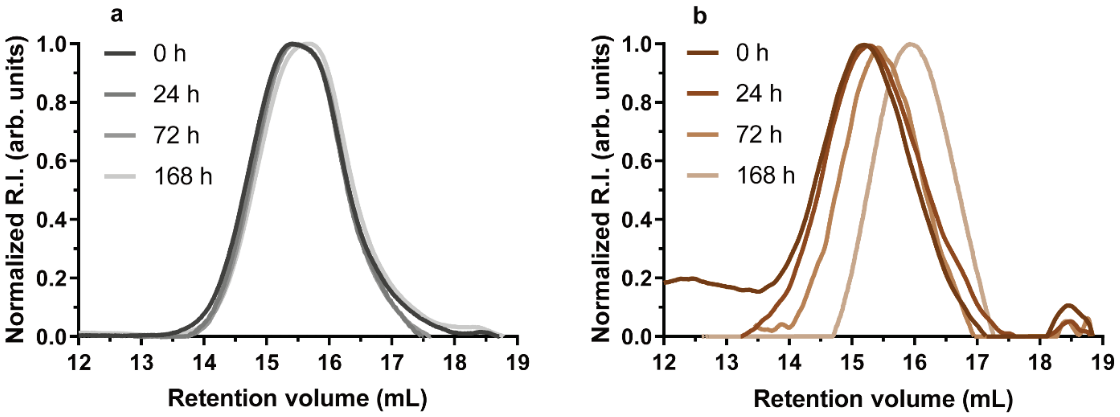

Supplementary Figure 21. Evolution of the SEC chromatograms at different time during hydrolytic degradation in PBS (pH 7.4, 37°C) of **a** PLA and **b** PLGA.

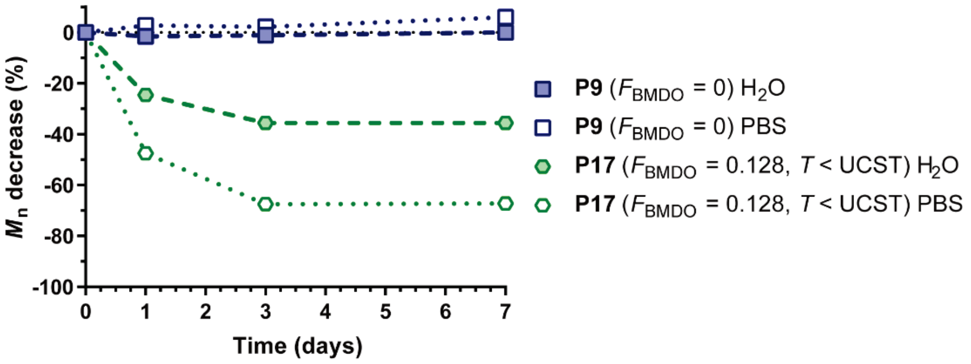

Supplementary Figure 22. Evolution of the number-average molar mass, M_n, with time during hydrolytic degradation of P(AAm-*co*-BMDO) copolymer **P17** (Table 2, **P17**) in PBS (pH 7.4, 37°C) and deionized water (pH 5.5) and comparison with P(AAm) **P9** (Table 2, **P9**).

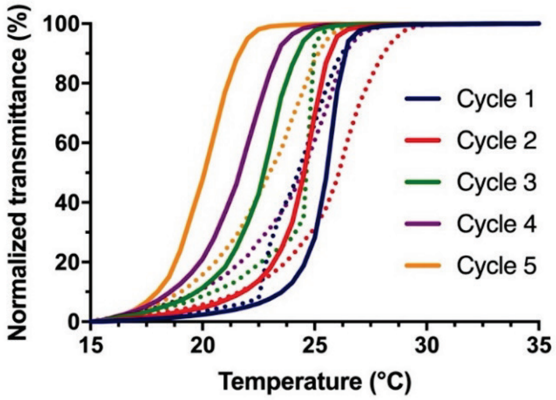

Supplementary Figure 23. Variation of the solution transmittance vs. temperature of P(AAm-*co*-BMDO) copolymer **P13** (Table 2) solution in water (10 mg.mL^{-1}) subjected to 5 consecutive cooling and heating cycles at 1°C.min^{-1} (over a total period of 7 h). Solid and dotted lines are for cooling and heating, respectively.

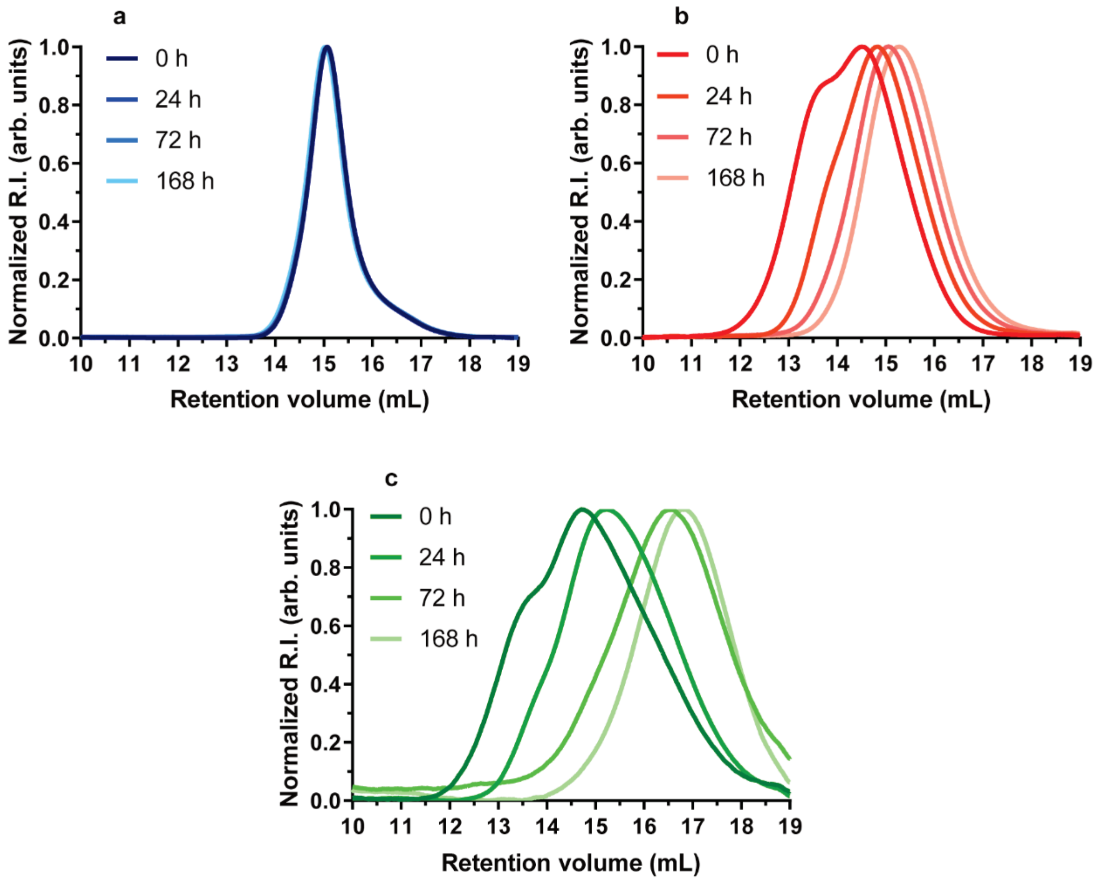

Supplementary Figure 24. Evolution of the SEC chromatograms at different time during hydrolytic degradation in PBS (pH 7.4, 37°C) of P(AAm-*co*-MDO) copolymers (Table 1, **P0**, **P6** and **P8**) as a function of F_{MDO}. **a P0** (F_{MDO} = 0, no T_{cp}); **b P6** (F_{MDO} = 0.18, no T_{cp}); **c P8** (F_{MDO} = 0.44, no T_{cp}).

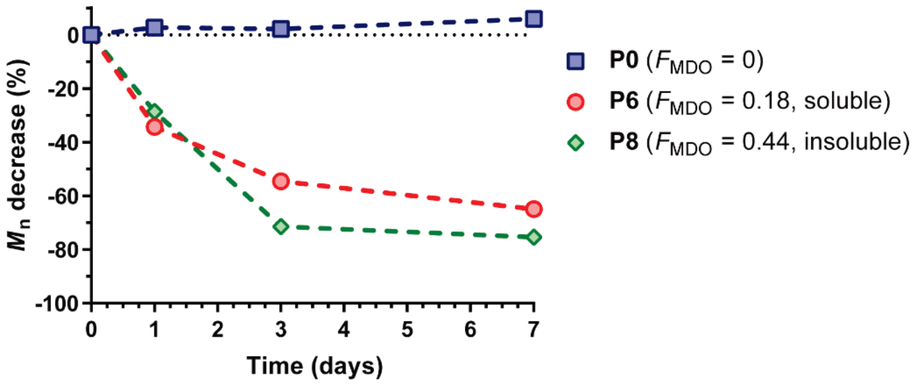

Supplementary Figure 25. Evolution of the number-average molar mass, M_n, with time during hydrolytic degradation in PBS (pH 7.4, 37°C) of P(AAm-*co*-MDO) copolymers (Table 1, **P6** and **P8**).

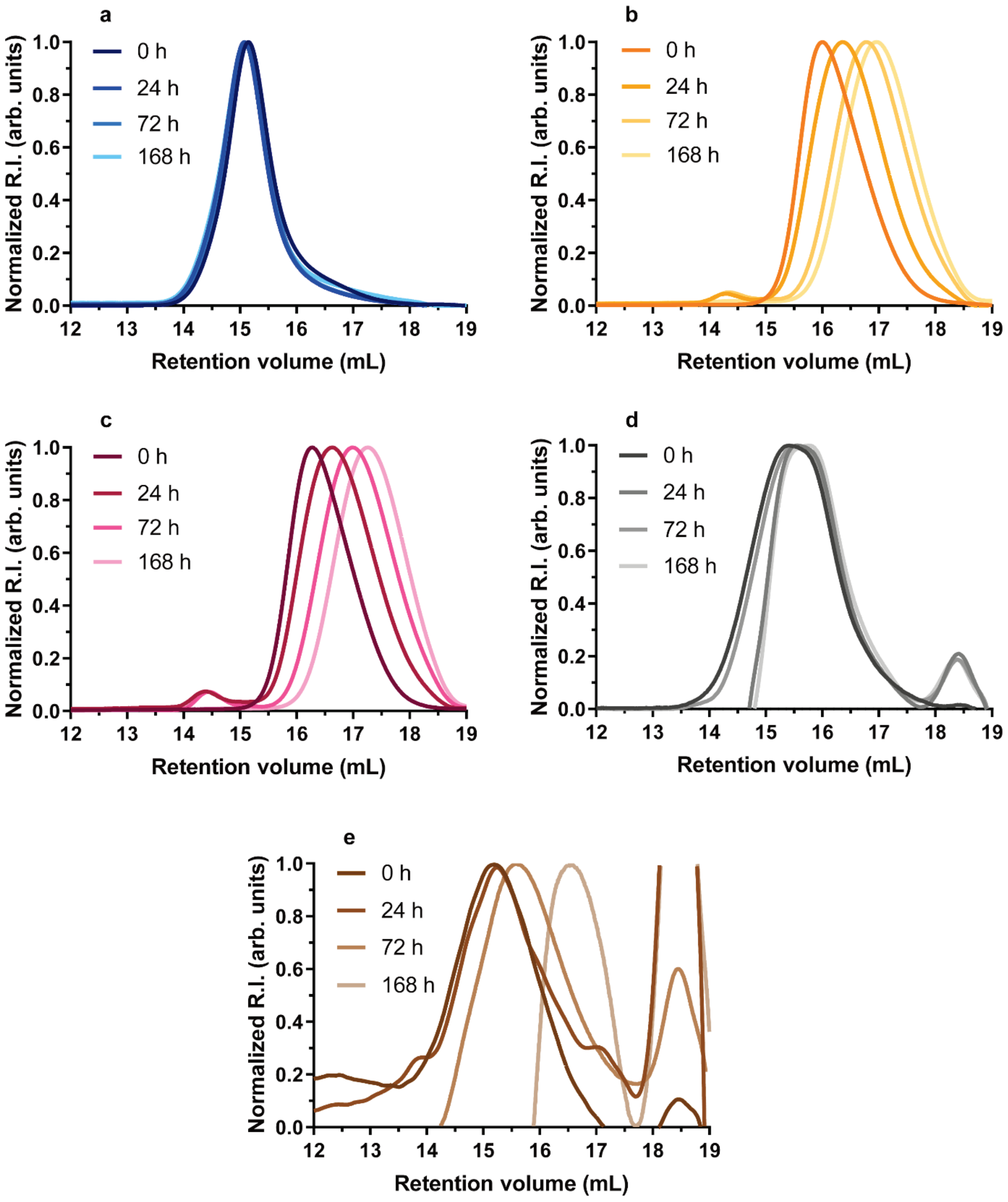

Supplementary Figure 26. Evolution of the SEC chromatograms at different time during enzymatic degradation (lipases from *Candida antartica*) in PBS (pH 7.4, 37°C) of P(AAm-*co*-BMDO) copolymers (Table 2, **P9**, **P13** and **P17**) as a function F_{BMDO}. **a P9** (F_{BMDO} = 0, no T_{cp}); **b P13** (F_{BMDO} = 0.093, T_{cp} = 25°C); **c P17** (F_{BMDO} = 0.128, T_{cp} = 52°C); **d** PLA; **e** PLGA.

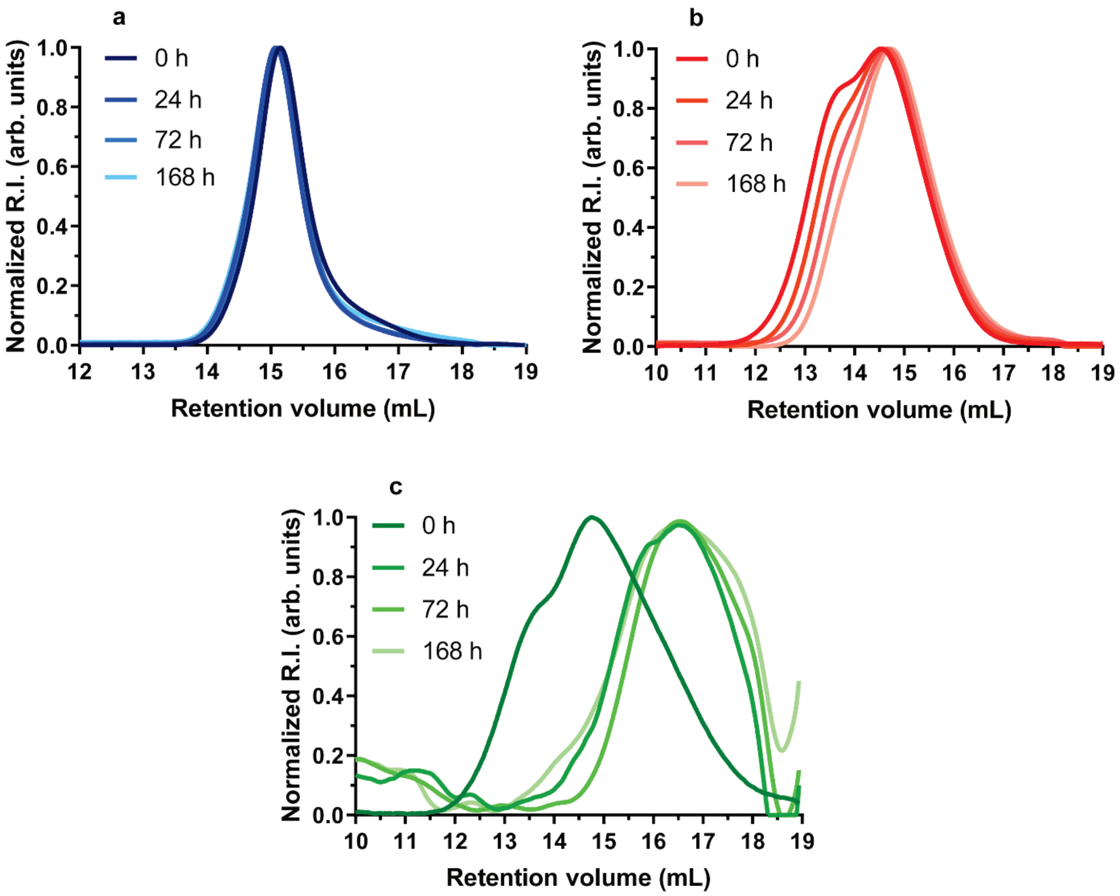

Supplementary Figure 27. Evolution of the SEC chromatograms at different time during enzymatic degradation (lipases from *Candida antartica*) in PBS (pH 7.4, 37°C) of P(AAm-*co*-MDO) copolymers (Table 1, **P0**, **P5** and **P8**) as a function of F_{MDO}. **a P0** (F_{MDO} = 0, no T_{cp}); **b P6** (F_{MDO} = 0.18, no T_{cp}); **c P8** (F_{MDO} = 0.44, no T_{cp}).

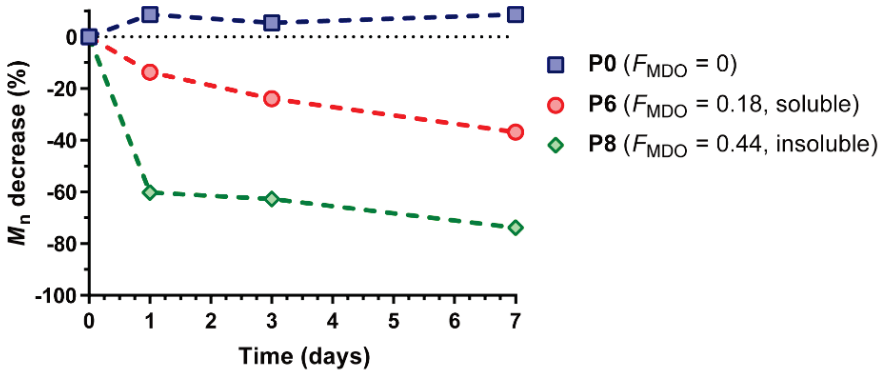

Supplementary Figure 28. Evolution of the number-average molar mass, M_n, with time during enzymatic degradation in PBS (pH 7.4, 37°C) of P(AAm-*co*-MDO) copolymers (Table 1, **P6** and **P8**).

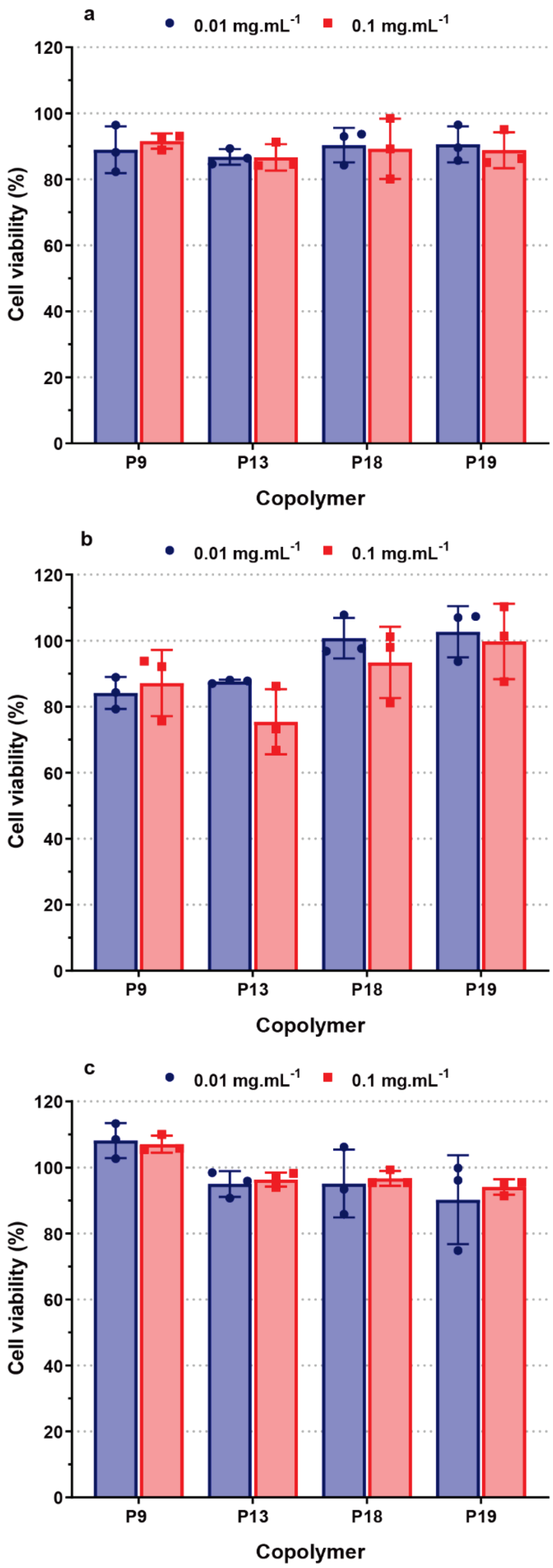

Supplementary Figure 29. Cell viability (MTT assay) after incubation of: **a** NIH/3T3 cells; **b** HUVEC cells and **c** J774.A1 cells with P(AAm-*co*-BMDO) copolymers as function of the BMDO content (**P9**, Table 2) and the M_n (**P13**, **P18** and **P19**, Supplementary Table 2) at 0.01 and 0.1 mg.mL^{-1}. Results were expressed as percentage of absorption of treated cells (n = 3 for each condition, error bars represent mean ± SD) in comparison with the values obtained from untreated control cells.

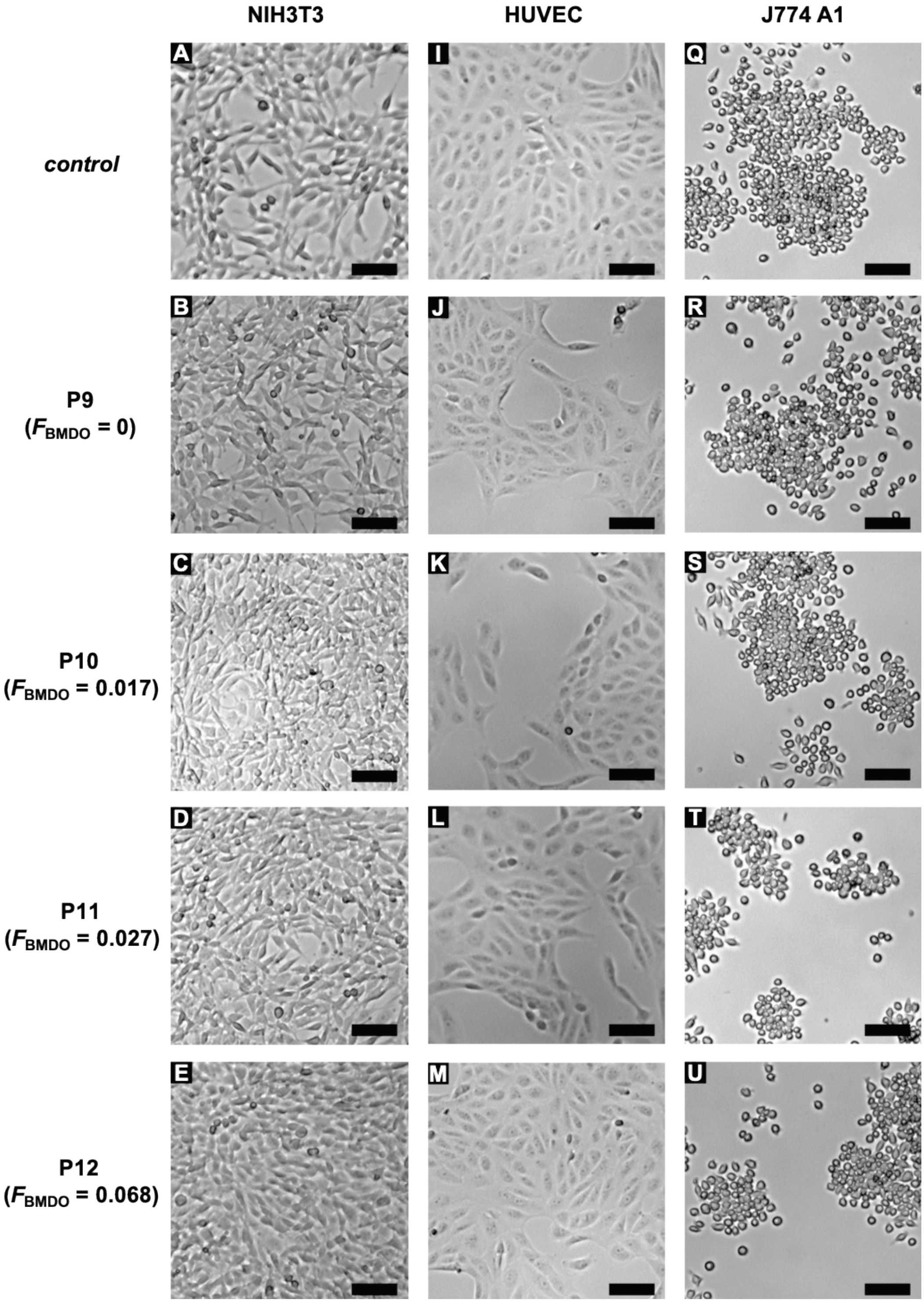
NIH3T3
HUVEC
J774 A1
control
P9
(F_{BMDO} = 0)
P10
(F_{BMDO} = 0.017)
P11
(F_{BMDO} = 0.027)
P12
(F_{BMDO} = 0.068)
A
B
C
D
E
I
J
K
L
M
Q
R
S
T
U

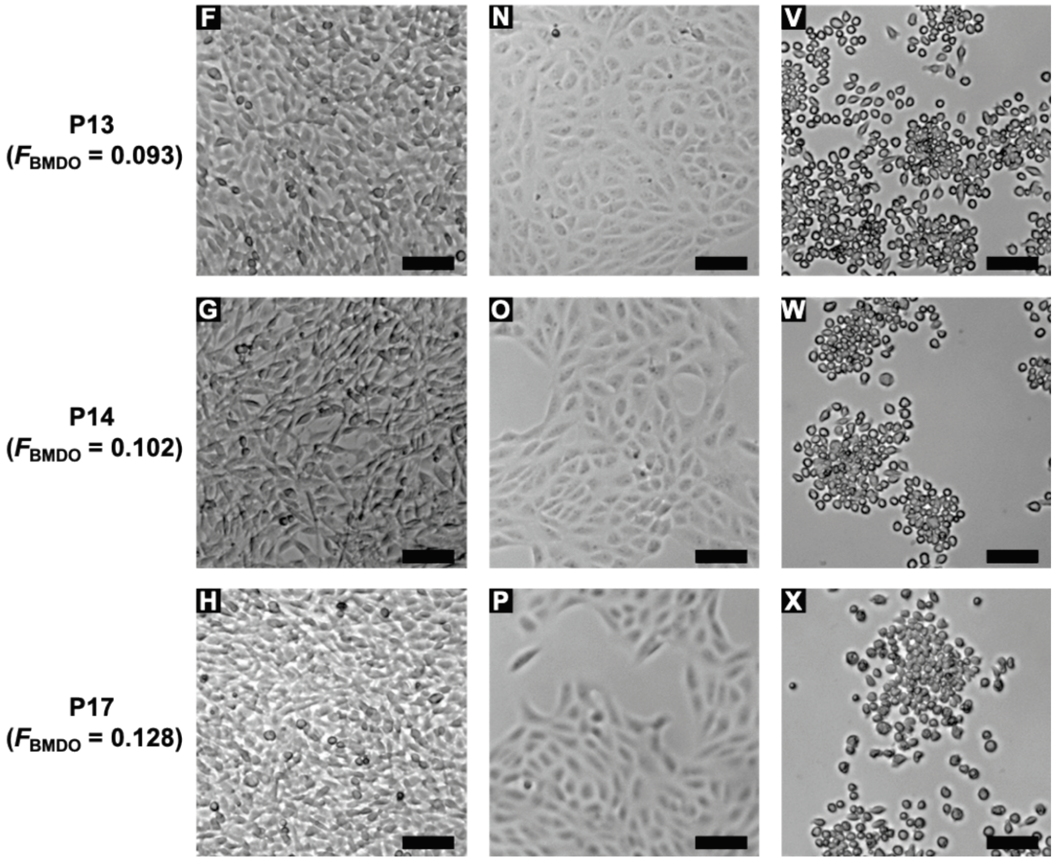

Supplementary Figure 30. Optical images of NIH/3T3 cells (first column, A-H), HUVEC cells (second column, G-P), and J774.A1 cells (third column, Q-X) taken by optical microscopy after treatment for 72 h with P(AAm-*co*-BMDO) copolymers (Table 2) (0.01 mg.mL^{-1}). Line 1: without treatment of copolymers; line 2: **P9**; line 3: **P10**; line 4: **P11**; line 5: **P12**; line 6: **P13**; line 7: **P14;** line 8: **P17**. Scale bar = 100 µm. These experiments were repeated three times with similar results.

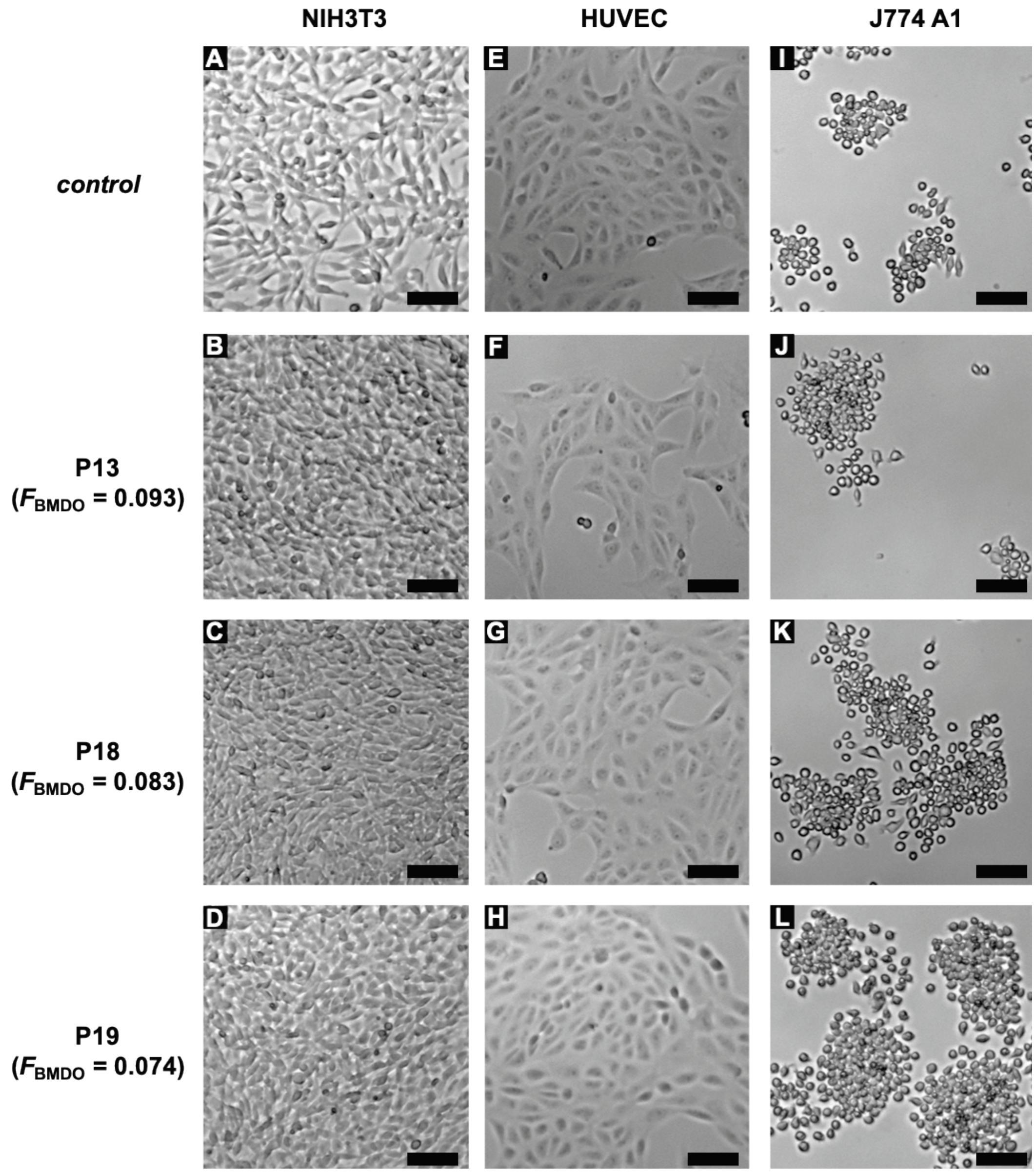

Supplementary Figure 31. Optical images of NIH/3T3 cells (first column, A-D), HUVEC cells (second column, E-H), and J774.A1 cells (third column, I-L) taken by optical microscopy after treatment for 72 h with P(AAm-*co*-BMDO) copolymers with $f_{BMDO,0}$ = 0.4 and different degrees of polymerization ($DP_{n, th}$) (Supplementary Table 2) (0.1 mg.mL^{-1}). Line 1: without treatment of copolymers; line 2: **P13**; line 3: **P18**; line 4: **P19**. Scale bar = 100 µm. These experiments were repeated three times with similar results.

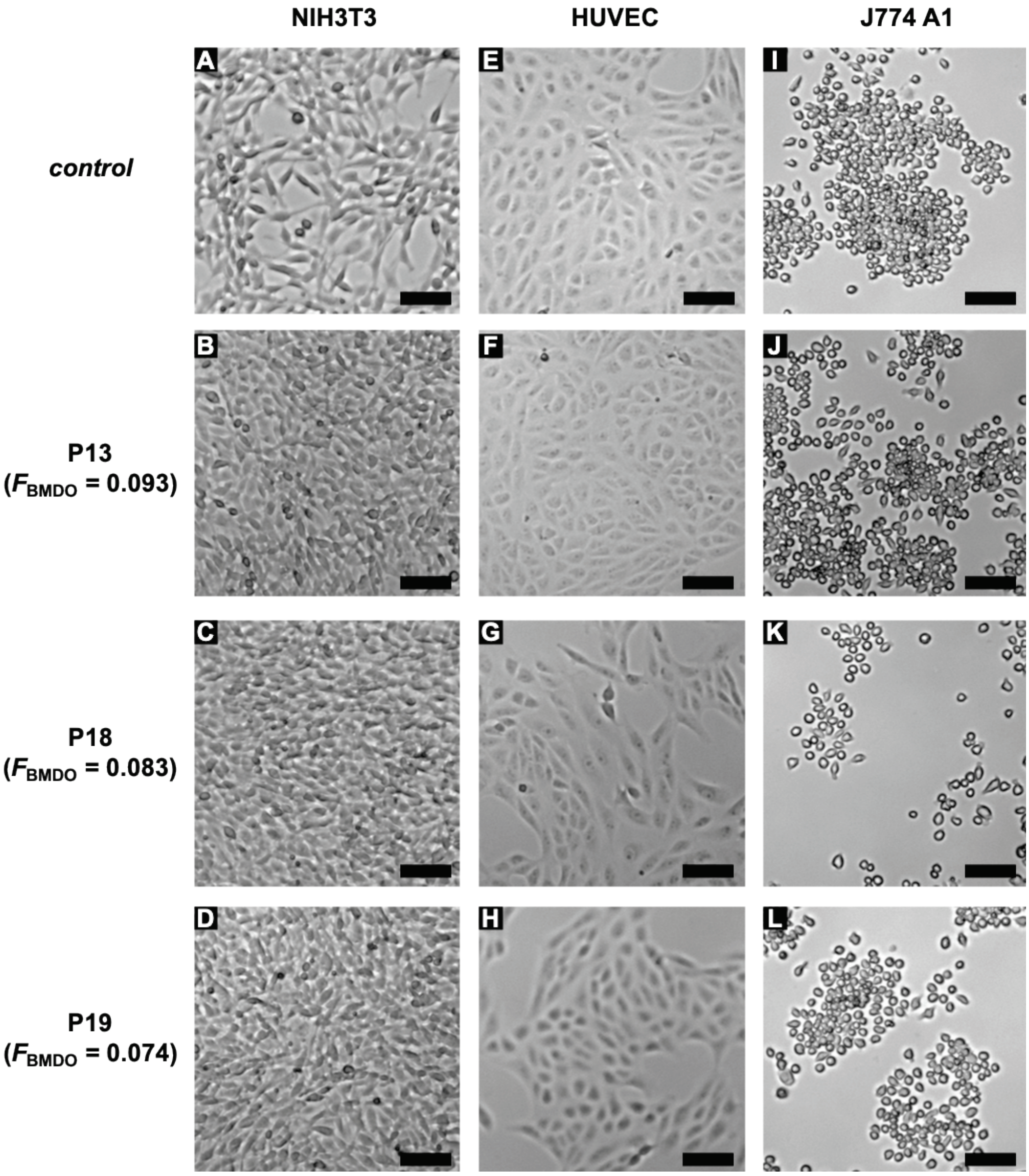

Supplementary Figure 32. Optical images of NIH/3T3 cells (first column, A-D), HUVEC cells (second column, E-H), and J774.A1 cells (third column, I-L) taken by optical microscopy after treatment for 72 h with P(AAm-*co*-BMDO) copolymers with $f_{BMDO,0}$ = 0.4 and different degrees of polymerization ($DP_{n,th}$) (Supplementary Table 2) (0.01 mg.mL^{-1}). Line 1: without treatment of copolymers; line 2: **P13**; line 3: **P18**; line 4: **P19**. Scale bar = 100 µm. These experiments were repeated three times with similar results.

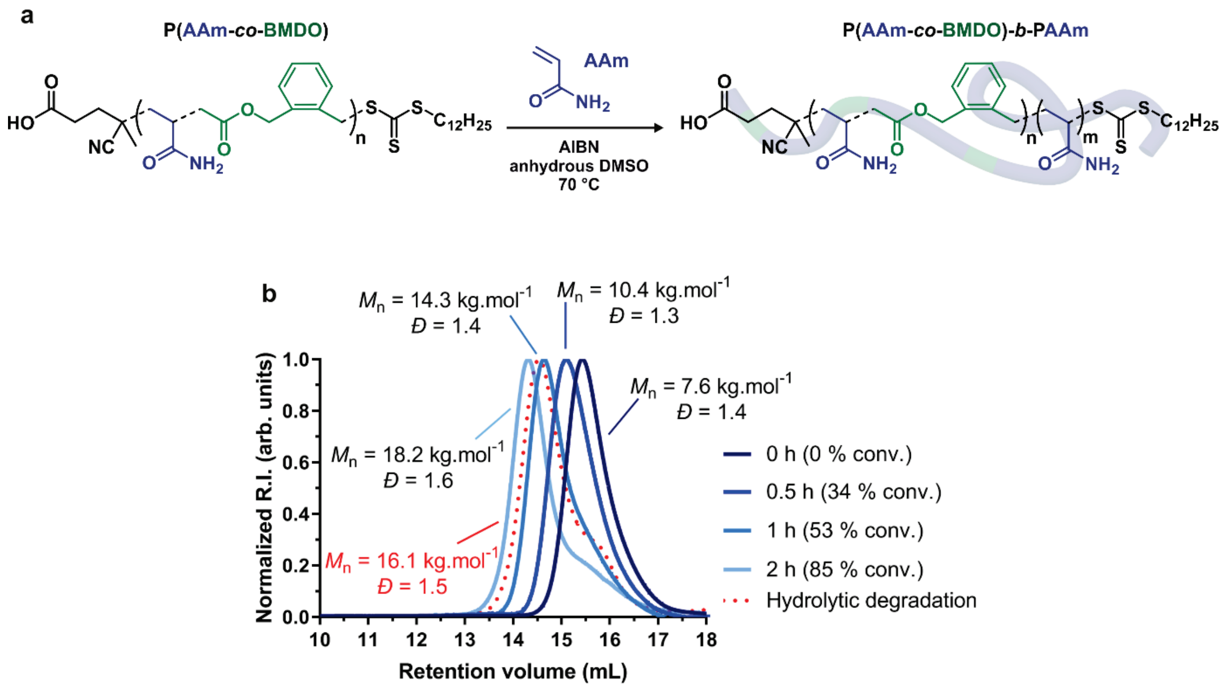

Supplementary Figure 33. a Structure of the P(AAm-*co*-BMDO)-*b*-PAAm diblock copolymer after chain extension of P(AAm-*co*-BMDO) copolymer with AAm via RAFT polymerization; **b** Evolution of the SEC chromatograms at different time/conversion during chain extension of P(AAm-*co*-BMDO) copolymer **P13** (Table 2) with AAm at 70 °C for 2 h at 0.8 M in anhydrous DMSO and after hydrolytic degradation (red dotted line).

Supplementary Table 4. Experimental Conditions and Macromolecular, Thermoresponsive, and Colloidal Characteristics of the Copolymer Nanoparticles Prepared by Nanoprecipitation of POEGMA-*b*-P(AAm-*co*-BMDO) and P(AAm-*co*-BMDO)-*b*-POEGMA Diblock Copolymers.

Entry	Macro-CTA	$M_{n,\,exp}$ first block ($g.mol^{-1}$)[a]	Feed	Conv (%)[b]	$M_{n,\,exp}$ diblock ($g.mol^{-1}$)[a]	$Đ$[a]	F_{BMDO}[c]	Open BMDO (%)[c]	T_{cp} UCST (°C)[d]		T_{cp} LCST (°C)[d]		D_z (nm)[e]	PSD[e]
									Cooling	Heating	Cooling	Heating		
**P20**	POEGMA	7,400	AAm/BMDO	55	8,800	1.9	0.064	86	12	17	73	73	188 ± 1	0.17 ± 0.02
**P21**	P(AAm-*co*-BMDO)	4,800	OEGMA	54	8,400	1.3	0.069	97	18	20	73	71	213 ± 2	0.10 ± 0.05

[a] Determined by SEC in DMSO with 100 mM LiBr using simple detection. [b] Determined by ^1H NMR by integrating the 2H of AAm (6.02–6.24 ppm) at t = 0 and 16 h. [c] Determined by ^1H NMR after precipitation by integrating the 2H (–NH_2) of AAm, the 4H (aromatic protons) of open and closed BMDO (6.5–7.5 ppm), the 2H of open BMDO (4.9–5.2 ppm) and the 4H of closed BMDO (4.5–4.8 ppm).[d] Determined from the maximum of the first derivative of the cooling/heating curves obtained by UV-vis temperature ramp (1°C.min^{-1}) at 10 mg.mL^{-1} in deionized water. [e] Intensity-average diameter and polydispersity measured by dynamic light scattering (DLS) as an average of three different measurements performed at 5°C (T < UCST)

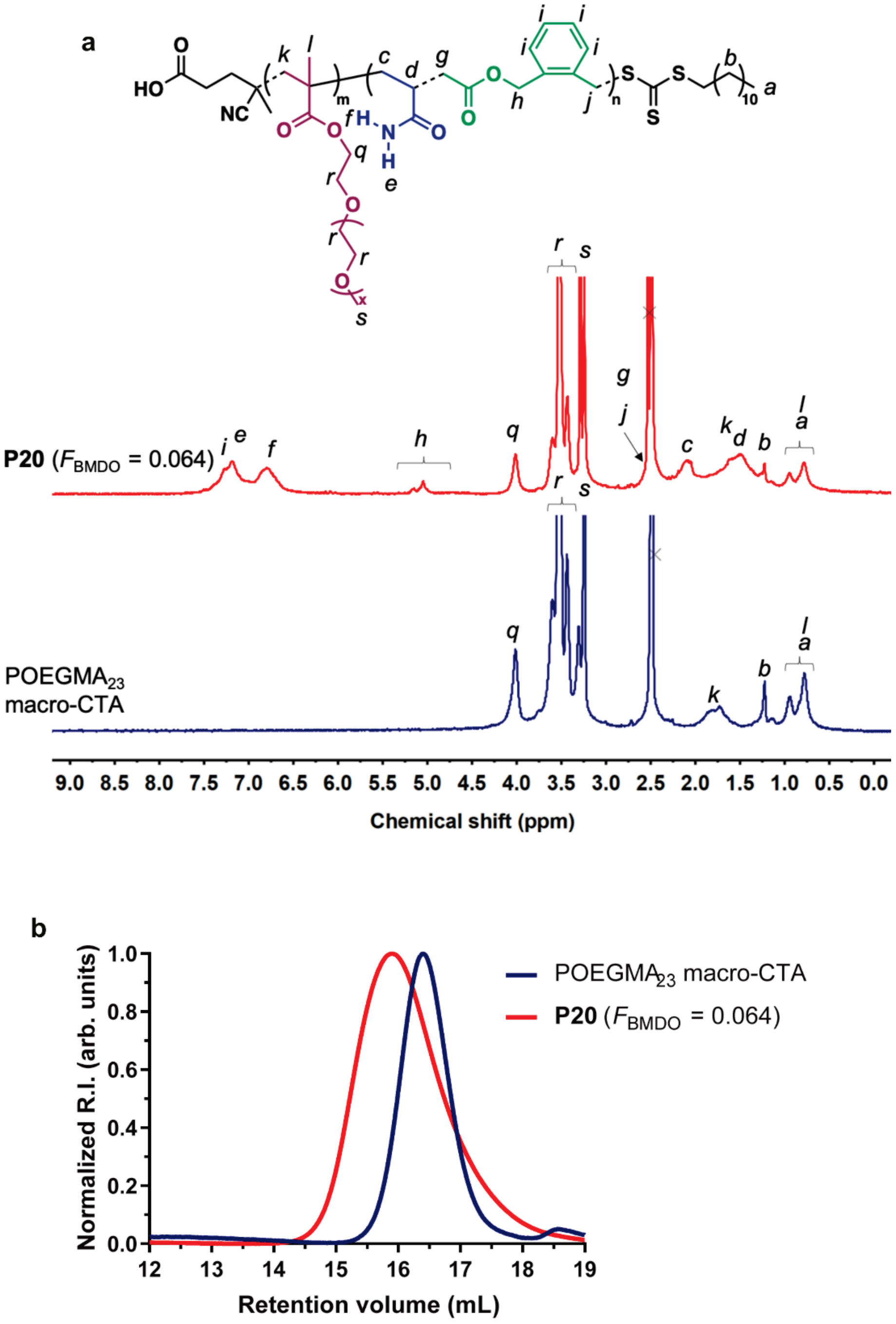

Supplementary Figure 34. a ^{1}H NMR spectra (300 MHz, DMSO-d_6) in the 0–9 ppm region of POEGMA$_{23}$ macro-CTA and POEGMA-*b*-P(AAm-*co*-BMDO) (Table 4, **P20**) copolymer. **b** Evolution of the SEC chromatograms during synthesis of amphiphilic diblock POEGMA-*b*-P(AAm-*co*-BMDO) (Supplementary Table 4, **P20**) showing a shift in retention volume.

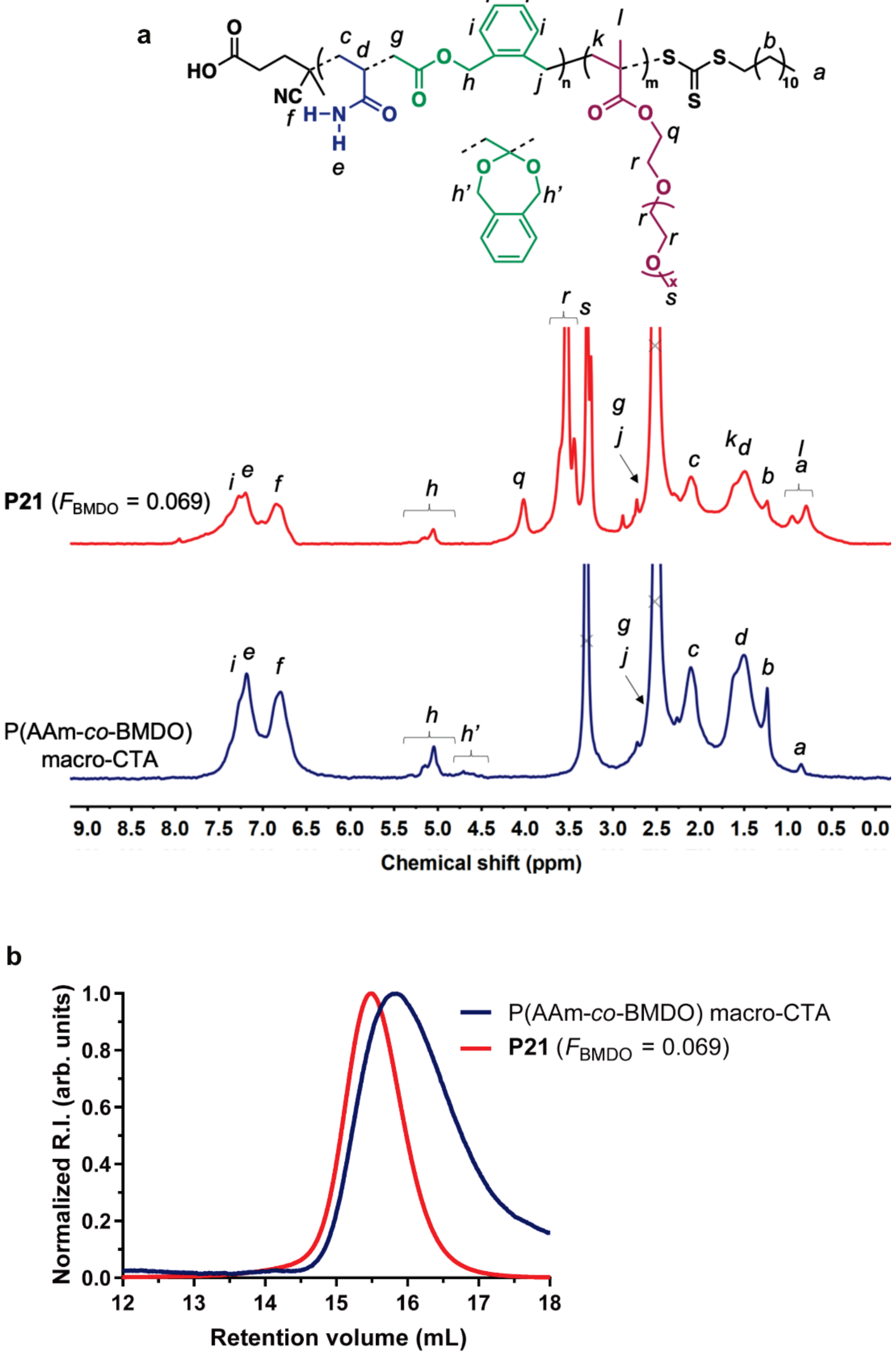

Supplementary Figure 35. a ^{1}H NMR spectra (300 MHz, DMSO-d$_6$) in the 0–9 ppm region of P(AAm-*co*-BMDO) macro-CTA and P(AAm-*co*-BMDO)-*b*-POEGMA (Table 4, **P21**) copolymer. **b** Evolution of the SEC chromatograms during synthesis of amphiphilic diblock P(AAm-*co*-BMDO)-*b*-POEGMA (Supplementary Table 4, **P21**) showing a shift in retention volume.

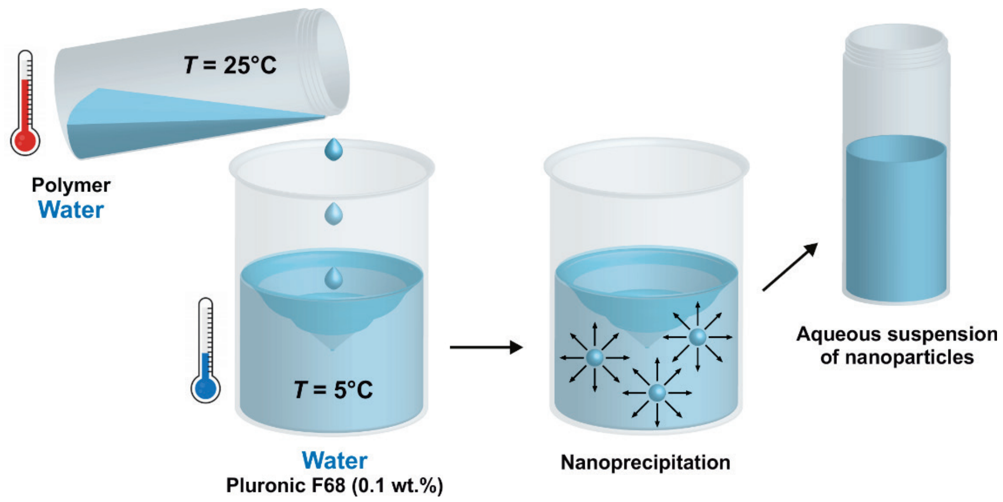

Supplementary Figure 36. Formulation of nanoparticle by an all-water nanoprecipitation process that consists in the dropwise addition of a polymer solubilized in water at T > UCST into an aqueous solution containing 0.1 wt. % of Pluronic F68 at 5°C (T < UCST) under stirring.

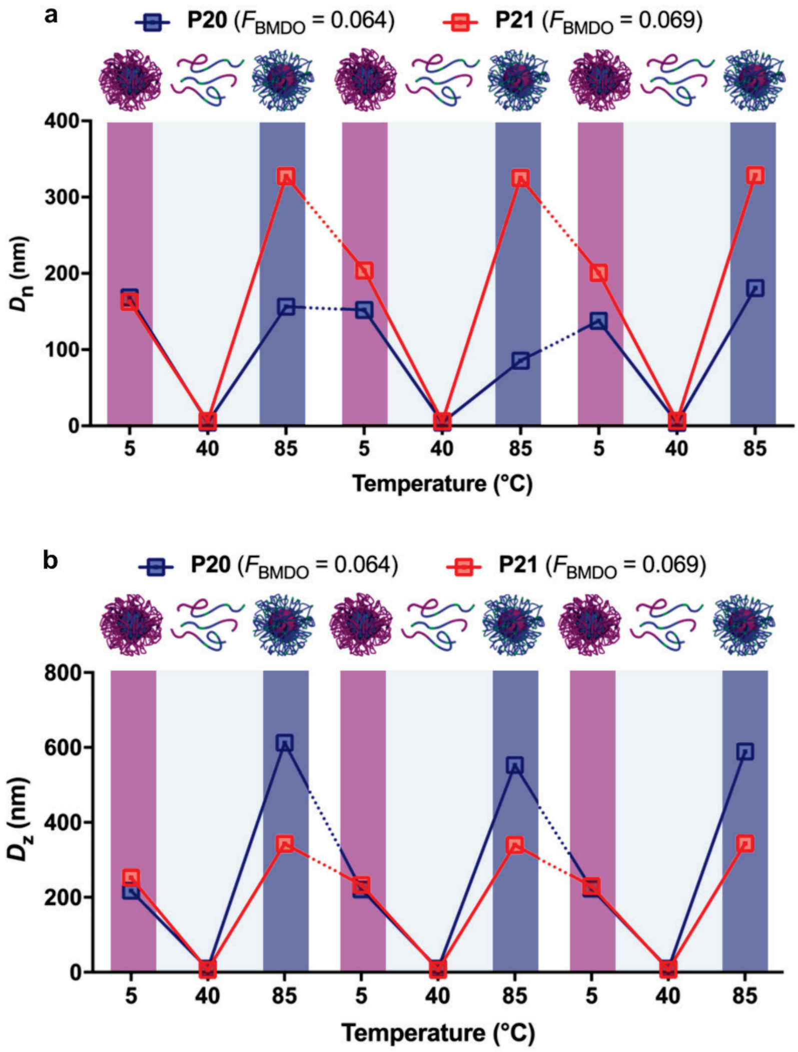

Supplementary Figure 37. Reversible change of the: **a** number-average diameter (D_n) and **b** intensity-average diameter (D_z) of the thermoresponsive POEGMA-*b*-P(AAm-*co*-BMDO) **P20** and P(AAm-*co*-BMDO)-*b*-POEGMA **P21** (Supplementary Table 4) diblock copolymers nanoparticles (1.67 mg.mL^{-1}) upon temperature shift between 5°C (insoluble state, T < UCST), 40°C (soluble state, UCST < T < LCST) and 85°C (insoluble state, T > LCST).

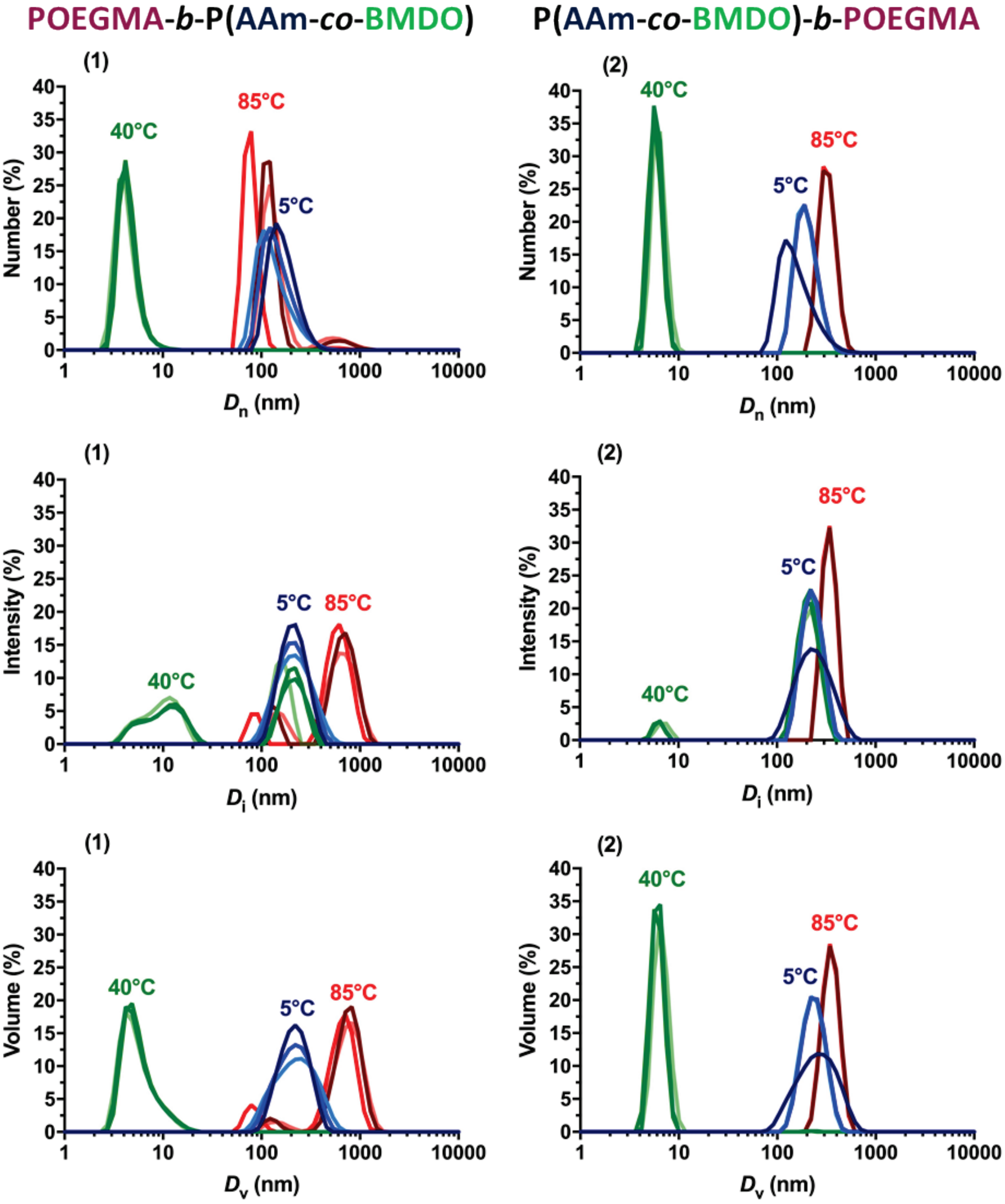

Supplementary Figure 38. Evolution of the number-average diameter (D_n, first line), intensity-average diameter (D_z, second line) and volume-average diameter (D_v, third line) of the doubly thermoresponsive P(AAm-*co*-BMDO)-*b*-POEGMA diblock copolymer nanoparticles **P20** (1) and **P21** (2) at 1.67 mg.mL^{-1} at T = 40°C, 5°C and 85°C.

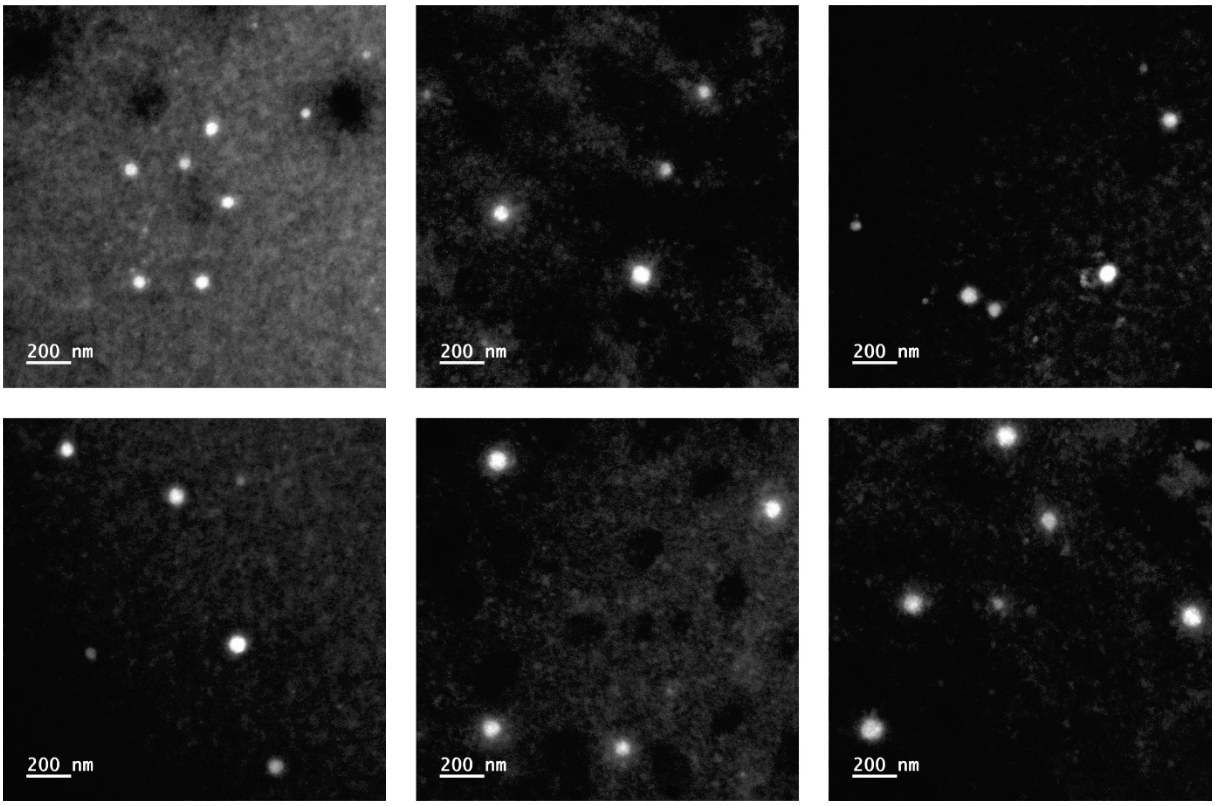

Supplementary Figure 39. TEM images with negative staining of nanoparticles **P20**. This experiment was performed once and 45 pictures were taken on different areas of the grid.

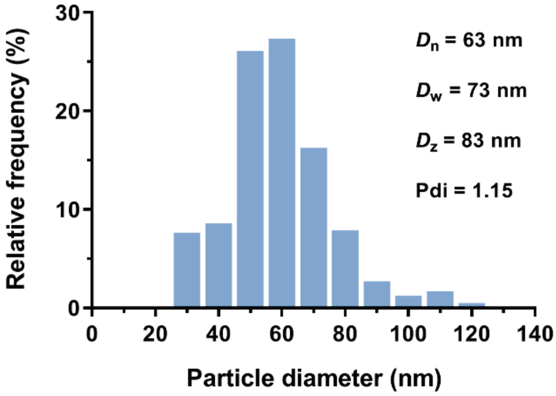

Supplementary Figure 40. Particle size distribution of nanoparticles **P20** from TEM images (n = 400) and calculation of the number-average (D_n), weight-average (D_w) and z-average (D_z) diameter, as well as the polydispersity index (Pdi), according to: $D_{\rm n} = \frac{\sum_i n_i \cdot D_i}{\sum_i n_i}$, $D_{\rm w} = \frac{\sum_i n_i \cdot D_i^4}{\sum_i n_i \cdot D_i^3}$, $D_{\rm z} = \frac{\sum_i n_i \cdot D_i^6}{\sum_i n_i \cdot D_i^5}$ and Pdi = D_w /D_n.

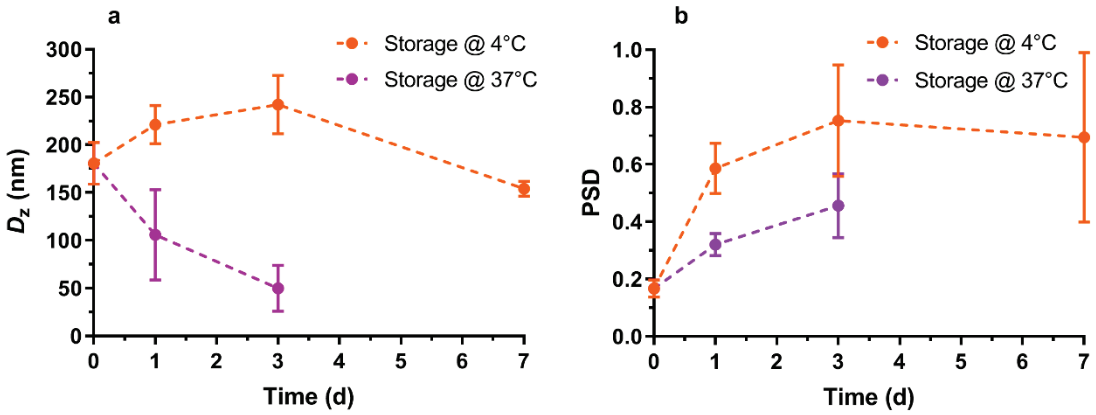

Supplementary Figure 41. Evolution of: **a** the intensity-average diameter (D_z) and **b** the particles size distribution (PSD) of POEGMA-*b*-P(AAm-*co*-BMDO) nanoparticles (**P20**) at 4°C and 37°C. The values are expressed as the means ± SD (n = 3).

Supplementary Table 5. Experimental Conditions and Macromolecular and Thermosensitive Properties of the of POEGMA-*b*-P(AAm-*co*-BMDO) Diblock Copolymer **P22**.

Macro-CTA	$M_{n,\,exp}$ first block ($g.mol^{-1}$)[a]	$Đ$[a]	$f_{BMDO,0}$	Feed	Conv (%)[b]	$M_{n,\,exp}$ diblock ($g.mol^{-1}$)[a]	$Đ$[a]	F_{BMDO}[c]	Open BMDO (%)[c]	T_{cp} UCST (°C)	
										Cooling[d]	Heating[e]
POEGMA	3,200	1.33	0.55	AAm/BMDO	79	7,700	1.55	0.126	89	40	42

[a] Determined by SEC in DMSO with 100 mM LiBr using simple detection. [b] Determined by ^{1}H NMR by integrating the 2H of AAm (6.02–6.24 ppm) at t = 0 and 16 h. [c] Determined by ^{1}H NMR after precipitation by integrating the 2H (–NH_2) of AAm, the 4H (aromatic protons) of open and closed BMDO (6.5–7.5 ppm), the 2H of open BMDO (4.9–5.2 ppm) and the 4H of closed BMDO (4.5–4.8 ppm).[d] Determined by DLS. [e] Determined from the maximum of the first derivative of the heating curve obtained by UV-vis temperature ramp ($1°C.min^{-1}$) at 10 $mg.mL^{-1}$ in deionized water.

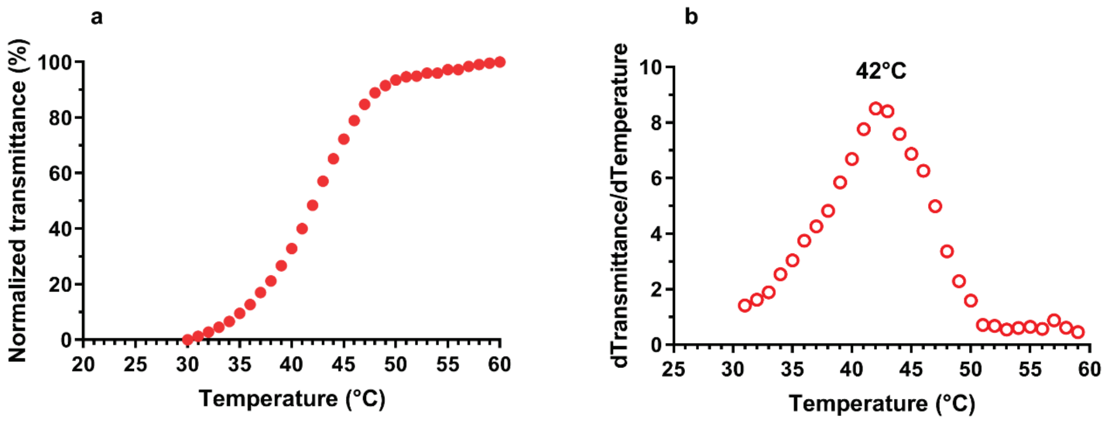

Supplementary Figure 42. a Variation of the solution transmittance vs. temperature of POEGMA-*b*-P(AAm-*co*-BMDO) copolymer **P22** solution in water (10 mg.mL^{-1}) upon heating at 1°C.min^{-1} and **b** of its first derivative.

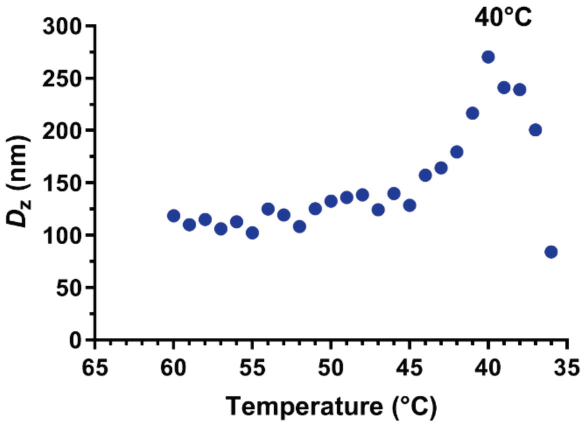

Supplementary Figure 43. Variation of the intensity average diameter (D_z) from DLS vs. temperature of POEGMA-*b*-P(AAm-*co*-BMDO) copolymer **P22** solution in water (10 mg.mL^{-1}) upon cooling.

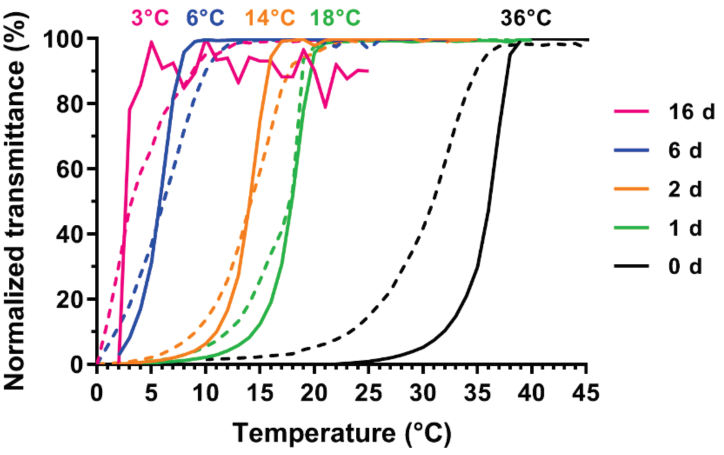

Supplementary Figure 44. Plot of solution transmittance versus temperature of POEGMA-*b*-P(AAm-*co*-BMDO) copolymer **P22** (Supplementary Table 5) solution in water (10 mg.mL^{-1}) subjected to cooling and heating cycles at 1°C.min^{-1} after 1, 2, 6 and 16 days. Solid and dotted lines are for cooling and heating, respectively. Note that T_{cp} at day 0 (36°C) is slightly lower than ~40°C due to partial degradation of the copolymer.
