# Supplementary material for: Vinyl copolymers with faster hydrolytic degradation than aliphatic polyesters and tunable upper critical solution temperatures
Source: Nat Commun. 2022 May 24;13:2873. doi: 10.1038/s41467-022-30220-y (PMC9130262; doi:10.1038/s41467-022-30220-y)
Supplement: Supplementary file 2 — Description of Additional Supplementary Files [file 41467_2022_30220_MOESM2_ESM.pdf]

Description of Additional Supplementary Files

Supplementary Movie 1. Time-lapse sequence (56 s, acceleration x6) of P(AAm-*co*-BMDO)-*b*-POEGMA **P20** diblock copolymer solution in water (10 mg.mL^{-1}) upon consecutive immersions into an ice water bath (2 °C) and a preheated oil bath (100°C).
