# Supplementary material for: Vinyl copolymers with faster hydrolytic degradation than aliphatic polyesters and tunable upper critical solution temperatures
Source: Nat Commun. 2022 May 24;13:2873. doi: 10.1038/s41467-022-30220-y (PMC9130262; doi:10.1038/s41467-022-30220-y)
Supplement: Supplementary file 4 — Reporting Summary [file 41467_2022_30220_MOESM4_ESM.pdf]

nature research

Corresponding author(s): Julien Nicolas

Last updated by author(s): 3/21/2022

Reporting Summary

Nature Research wishes to improve the reproducibility of the work that we publish. This form provides structure for consistency and transparency in reporting. For further information on Nature Research policies, see our Editorial Policies and the Editorial Policy Checklist.

Statistics

For all statistical analyses, confirm that the following items are present in the figure legend, table legend, main text, or Methods section.

n/a	Confirmed	
☐	☒	The exact sample size (n) for each experimental group/condition, given as a discrete number and unit of measurement
☐	☒	A statement on whether measurements were taken from distinct samples or whether the same sample was measured repeatedly
☒	☐	The statistical test(s) used AND whether they are one- or two-sided<br>*Only common tests should be described solely by name; describe more complex techniques in the Methods section.*
☒	☐	A description of all covariates tested
☒	☐	A description of any assumptions or corrections, such as tests of normality and adjustment for multiple comparisons
☐	☒	A full description of the statistical parameters including central tendency (e.g. means) or other basic estimates (e.g. regression coefficient) AND variation (e.g. standard deviation) or associated estimates of uncertainty (e.g. confidence intervals)
☒	☐	For null hypothesis testing, the test statistic (e.g. F, t, r) with confidence intervals, effect sizes, degrees of freedom and P value noted<br>*Give P values as exact values whenever suitable.*
☒	☐	For Bayesian analysis, information on the choice of priors and Markov chain Monte Carlo settings
☒	☐	For hierarchical and complex designs, identification of the appropriate level for tests and full reporting of outcomes
☒	☐	Estimates of effect sizes (e.g. Cohen's d, Pearson's r), indicating how they were calculated

Our web collection on statistics for biologists contains articles on many of the points above.

Software and code

Policy information about availability of computer code

Data collection	OmniSEC 4.0, Malvern-Zetasizer 7.12, Winlab Version:6.0.3.0730, Zen 2.6 software (blue edition), RS Image V1.9.2, ImageJ 1.53a
Data analysis	MestReNova 11.0.4, OmniSEC 4.0, Malvern-Zetasizer 7.12, Zen 2.6 software (blue edition), RS Image V1.9.2, Data Processor and Viewer (DPV) Version: 1.00.100.0010, iMovie 10.2.4, Image J 1.53a

For manuscripts utilizing custom algorithms or software that are central to the research but not yet described in published literature, software must be made available to editors and reviewers. We strongly encourage code deposition in a community repository (e.g. GitHub). See the Nature Research guidelines for submitting code & software for further information.

Data

Policy information about availability of data

All manuscripts must include a data availability statement. This statement should provide the following information, where applicable:

- Accession codes, unique identifiers, or web links for publicly available datasets
- A list of figures that have associated raw data
- A description of any restrictions on data availability

Data availability
Source data files are available for the data shown in Figures 3A, 4A, 4F, 7A, Supplementary Figures 29 and 40. All other data generated and analyzed during this study are included in this article and its Supplementary Information and are also available from the corresponding author upon request.

Field-specific reporting

Please select the one below that is the best fit for your research. If you are not sure, read the appropriate sections before making your selection.

☒ Life sciences ☐ Behavioural & social sciences ☐ Ecological, evolutionary & environmental sciences

For a reference copy of the document with all sections, see nature.com/documents/nr-reporting-summary-flat.pdf

Life sciences study design

All studies must disclose on these points even when the disclosure is negative.

Sample size	Sample size are provided in the experimental part and in the figure legends for each in vitro experiment (MTT assay). Reasonnable sample sizes were chosen to ensure they are sufficient for statistical comparison between different groups (usually n = 3). For MTT assay, 3 independant complete MTT experiments were conducted to afford statistically significant means and standard deviations, as routinely performed in the field.
Data exclusions	No data was excluded from the analyses
Replication	All MTT experiments were performed in triplicate to determine means and SD. All attemps at replication were successful. For other experiments, the number of replication is indicated in the legend of each figure, if needed.
Randomization	Not applicable considering there is no in vivo experiment
Blinding	Not applicable considering there is no in vivo experiment

Reporting for specific materials, systems and methods

We require information from authors about some types of materials, experimental systems and methods used in many studies. Here, indicate whether each material, system or method listed is relevant to your study. If you are not sure if a list item applies to your research, read the appropriate section before selecting a response.

Materials & experimental systems

n/a	Involved in the study	
☒	☐	Antibodies
☐	☒	Eukaryotic cell lines
☒	☐	Palaeontology and archaeology
☒	☐	Animals and other organisms
☒	☐	Human research participants
☒	☐	Clinical data
☒	☐	Dual use research of concern

Methods

n/a	Involved in the study	
☒	☐	ChIP-seq
☒	☐	Flow cytometry
☒	☐	MRI-based neuroimaging

Eukaryotic cell lines

Policy information about cell lines

Cell line source(s)	Human endothelial umbilical vein cells (HUVEC), embryonic murine fibroblast (NIH/3T3) and murine macrophage-monocyte cells (J774.A1) were purchased from American Type Culture Collection (ATCC) and maintained as recommended.
Authentication	Cell lines were used within 3 months after receipt or resuscitation of frozen aliquots thawed from liquid nitrogen. The provider assured the authentication of these cell lines by cytogenetic analysis. Cell lines were authenticated by morphological evaluation.
Mycoplasma contamination	The cell lines were tested mycoplasma every month. No contamination was found during this study.
Commonly misidentified lines (See ICLAC register)	No commonly misindentified cell lines were used in this study.
